# Supplementary material for: Validation of a Culturally Relevant Snakebite Envenomation Clinical Practice Guideline in Brazil
Source: Toxins (Basel). 2022 May 28;14(6):376. doi: 10.3390/toxins14060376 (PMC9229773; doi:10.3390/toxins14060376)
Supplement: Supplementary file 1 [file toxins-14-00376-s001.zip › File S1.pdf]

toxins

MDPI

SAVING

Snake Antivenom Immunoglobulins
NEED TO BE GUARANTEED

Guide to the treatment of **snakebites**

toxins

MDPI

2022

toxins

MDPI

DATA SHEET

Authors

Jacqueline de Almeida Gonçalves Sachett
Fundação de Dermatologia Tropical e Venereologia Alfredo da Matta (FUAM)
Universidade do Estado do Amazonas (UEA)

Fan Hui Wen
Instituto Butantã (IBU)

Wuelton Marcelo Monteiro
Fundação de Medicina Tropical Dr. Heitor Vieira Dourado (FMT-HVD)
Universidade do Estado do Amazonas (UEA)

Collaborators

Alícia Cacau Santos
Fundação de Medicina Tropical Dr. Heitor Vieira Dourado (FMT-HVD)
Universidade do Estado do Amazonas (UEA)

Altair Seabra Farias
Fundação de Medicina Tropical Dr. Heitor Vieira Dourado (FMT-HVD)
Universidade do Estado do Amazonas (UEA)

André Sachett
Fundação de Medicina Tropical Dr. Heitor Vieira Dourado (FMT-HVD)
Universidade do Estado do Amazonas (UEA)

Érica da Silva Carvalho
Fundação de Medicina Tropical Dr. Heitor Vieira Dourado (FMT-HVD)
Universidade do Estado do Amazonas (UEA)

Gisele dos Santos Rocha
Fundação de Medicina Tropical Dr. Heitor Vieira Dourado (FMT-HVD)
Universidade do Estado do Amazonas (UEA)

Joseir Saturnino Cristino
Fundação de Medicina Tropical Dr. Heitor Vieira Dourado (FMT-HVD)
Universidade do Estado do Amazonas (UEA)

Leonardo Marques
Fundação de Medicina Tropical Dr. Heitor Vieira Dourado (FMT-HVD)
Universidade do Estado do Amazonas (UEA)

Lisele Brasileiro
Fundação de Medicina Tropical Dr. Heitor Vieira Dourado (FMT-HVD)
Universidade do Estado do Amazonas (UEA)

Mena Bianca Ferreira
Fundação de Medicina Tropical Dr. Heitor Vieira Dourado (FMT-HVD)
Universidade do Estado do Amazonas (UEA)

Pedro Ferreira Bisneto
Universidade Federal do Amazonas (UFAM)

Vinícius Azevedo Machado
Universidade do Estado do Amazonas (UEA)

Yasmin Vieira Rocha
Fundação de Medicina Tropical Dr. Heitor Vieira Dourado (FMT-HVD)
Universidade do Estado do Amazonas (UEA)

Graphics and diagramming

João Arthur Alcântara
Fundação de Medicina Tropical Dr. Heitor Vieira Dourado (FMT-HVD)
Universidade do Estado do Amazonas (UEA)

Textual review

Vinicius Milhomem
Universidade Federal do Amazonas (UFAM)

Validation

Ageane Mota da Silva
Instituto Federal do Acre (IFAC)

Aline Cristiane Côrtes de Alencar
Fundação de Medicina Tropical Dr. Heitor Vieira Dourado (FMT-HVD)

Ceila Maria Sant'Ana Málaque
Instituto Butantã (IBU)
Instituto de Infectologia Emílio Ribas

Eliane Campos Alves
Instituto Federal do Amazonas (IFAM)

Elielza Guerreiro Menezes
Universidade do Estado do Amazonas (UEA)

Flavio Santos Dourado
Ministério da Saúde (MS)

Giliard Santos da Costa
Hemonúcleo de Cruzeiro do Sul (AC)

Gisele Torrente
Universidade do Estado do Amazonas (UEA)

Guilherme Augusto Pivoto João
Fundação de Medicina Tropical Dr. Heitor Vieira Dourado (FMT-HVD)

toxins

MDPI

Henry Maia Peixoto
Universidade de Brasília (UnB)

Iran Mendonça da Silva
Universidade do Estado do Amazonas (UEA)

Jaime Enrique Castro Valencia
Ministério da Saúde (MS)

Lúcia Regina Montebello Pereira
Ministério da Saúde (MS)

Márcia Almeida de Araújo Alexandre
Fundação de Medicina Tropical Dr. Heitor Vieira Dourado (FTM-HVD)

Maria Regina Fernandes de Oliveira
Universidade de Brasília (UnB)

Mariana Margarida Martinez Quiroga
Secretaria Municipal de Saúde de Santarém – PA (SEMSA)
Hospital Regional do Baixo Amazonas do Pará Dr. Waldemar Penna (HRBA)

Sâmella Silva de Oliveira
Hospital do Coração Francisca Mendes (HCFM)

Sandra Maria Deotti Carvalho
Ministério da Saúde (MS)
Secretaria de Estado da Saúde do Tocantins

Sílvio Cesar Pereira Fragoso
Fundação de Medicina Tropical Dr. Heitor Vieira Dourado (FTM-HVD)

Vanine de Lourdes Lima Fragoso
Fundação de Medicina Tropical Dr. Heitor Vieira Dourado (FTM-HVD)

Manaus, AM, 2022

toxins MDPI

PRESENTATION

For victims of snakebite to be successfully treated and to recover completely, they need to have access to antivenoms and other care procedures. This guide was created as a result of the concern of researchers and care professionals working in the field of clinical research and care of patients with snakebites in Brazil. Through their clinical and scientific experience, the researchers and clinicians identified that the problem was directly related to the work process of health professionals, who have little knowledge regarding the care management of these patients and feel insecure during their management. As a result, a group of specialists (two from the Dr. Heitor Vieira Dourado Tropical Medicine Foundation (FMT-HVD), a tertiary reference hospital for the treatment of snakebites in the Brazilian Amazon, and one from the Butantan Institute (IBU), the largest producer of antivenoms in the country, and which also treats cases of snakebite in the city of São Paulo, created the first version of a guide for the management of snakebites, and subsequently coordinated its content and semantic validation.

The Brazilian Ministry of Health has official guidelines with general recommendations for the management of snakebites and other animal envenomings. This guideline presents the epidemiology and general clinical aspects of envenomings of the four genera of snakes of medical importance in the country (*Bothrops, Lachesis, Crotalus* and *Micrurus*), as well as the available antivenoms and their dosages according to the severity of the case. The Brazilian guideline was created based on expert opinions who have experience with patients treated in hospitals, under medical supervision. The Ministry of Health guideline does not address pre-hospital care and first aid; storage, preparation and administration of antivenoms; wound treatment; auxiliary treatment of different local and systemic manifestations; referral to health services of greater complexity; discharge criteria; determination of coagulation time; and notification of cases to the epidemiological surveillance systems.

It should be noted that many of these procedures can be performed by the paramedic team. This guide also includes detailed information for the possible management of cases in basic health units, depending on local policies for the treatment of snakebite and future plans for decentralization of snakebite treatment in the Brazilian Amazon. The dosage of antivenoms is presented following the same recommendations as the Ministry of Health, since clinical studies have shown that the dosages of standard antivenoms are effective in reversing the systemic effects of envenomings.

Researchers working in the field of snakebites and experienced health professionals in the care of this condition were invited to participate as expert judges of this study, to validate the content of the guide. The judges were affiliated with institutions in the states of Acre, Amazonas, Tocantins, Federal District and São Paulo, thereby ensuring a culturally and geographically diverse sample.

After this stage, the suggestions of the judges were considered and a second version of the guide was generated, which was subjected to semantic evaluation by professionals of the basic health network of the municipalities of Careiro da Várzea, Ipixuna and Boa Vista do Ramos, in the state of Amazonas. After incorporating the considerations of the professionals, the final version of the guide was prepared.

The authors of this CPG thank the entire team responsible for the research and all the judges who participated in the content validation, as well as the health professionals who aided in the semantic validation of this guide. We are also grateful for the support of

the municipal health departments that allowed their professionals to receive training and participate in the validation process in Manaus. We thank the support of the president director of FMT-HVD, Dr. Marcus Vinicius de Farias Guerra, and the director of medical assistance of this institution, Dr. Antonio Magela, who facilitated the realization of all stages of the project. The study was approved by the Research Ethics Committee of the Amazonas State University (CAAE: 35855820.2.0000.5016). The authors also thank the funding granted by the Ministry of Health (733781/19-035), the Amazonas State Research Support Foundation (PRÓ-ESTADO - 011/2021, PCGP/FAPEAM - 010/2021 and CT&I ÁREAS PRIORITÁRIAS) and the Fogarty International Center of the National Institutes of Health (R21TW011944). The content of this guide is the sole responsibility of the authors and does not necessarily represent the official opinions of the sponsors.

This validated guide can be integrated into the curricula of health professionals at all levels in order to improve the quality of care in snakebites and thereby increase their knowledge and clinical competence. Finally, our findings support the use of this validated guide in various epidemiological and cultural contexts in Brazil.

Fan Hui Wen

Jacqueline Sachett

Wuelton Monteiro

toxins MDPI

SUMMARY

AO1	First aid	8
AO2	Diagnosis and clinical classification of the envenoming	12
AO3	Conducting the clotting time test	17
AO4	Preparation of the antivenom for administration	19
AO5	Antivenom treatment	23
AO6	Care of the bite site	29
AO7	Patient follow-up	33
AO8	Referral of the patient to a hospital	36
AO9	Evaluation of the complications of the snakebite	38
AO10	Hospital discharge and outpatient follow-up	41
AO11	Basic health unit discharge and follow-up	42
AO12	Filling out the notification form	43
AO13	Receiving and storing the antivenom	46

AO1 – First aid

When to suspect a bite/inclusion criteria:

On suspicion of any contact with a snake.

Procedure:

1. Assess patient responsiveness (call the patient's name);
- If not responsive, seek help (SAMU) and check the patient's pulse;
- In the absence of a pulse, initiate basic life support actions according to the protocols of the American Heart Association;
- During the cycles basic life support, check the pulse every 2 minutes;
- If responsive, continue the evaluation.

2. Assess the environment where the snakebite occurred and where the patient is;
- Check if the snake is still close to the patient and move it away;
- Do not try to capture the snake that caused the accident, as there is a risk of being bitten. However, if the patient has already killed the snake, they can take it (preferably in a closed bottle with alcohol) or take a clear photo for identification in the health unit, since its correct identification allows a more accurate diagnosis of the bite.
- Assess whether there is a risk of other injuries in the environment with other animals and/or objects.

3. Assess the site of the bite and any manifestations of the bite
- Verify the presence of a punctiform injury on the patient's skin or excoriation caused by the snakebite;
- Wash the area of the bite with clean water and soap, if possible;
- Observe the local and systemic signs and symptoms during transport, such as edema, pain intensity, skin coloration, presence of bleeding, among others;
- If possible, prevent the patient from moving; keep the affected limb in a comfortable position, preferably in an elevated position until arrival at the health unit.

4. Keep the patient hydrated
- If the patient is responsive, let them have as much water as they want, though with care to prevent them from vomiting.

5. Get the patient to the nearest healthcare facility as soon as possible.

Note:
- Never use a tourniquet or perform any other procedure at the site of the bite such as suction, incision, application of substances and homemade products, since, in addition to a lack of evidence of their effectiveness, they can cause further complications at the site of the injury;

- Never recommend to the patient to ingest homemade remedies: teas, tinctures or other preparations, as there is also no evidence for neutralization of the venom or other benefit to the patient.

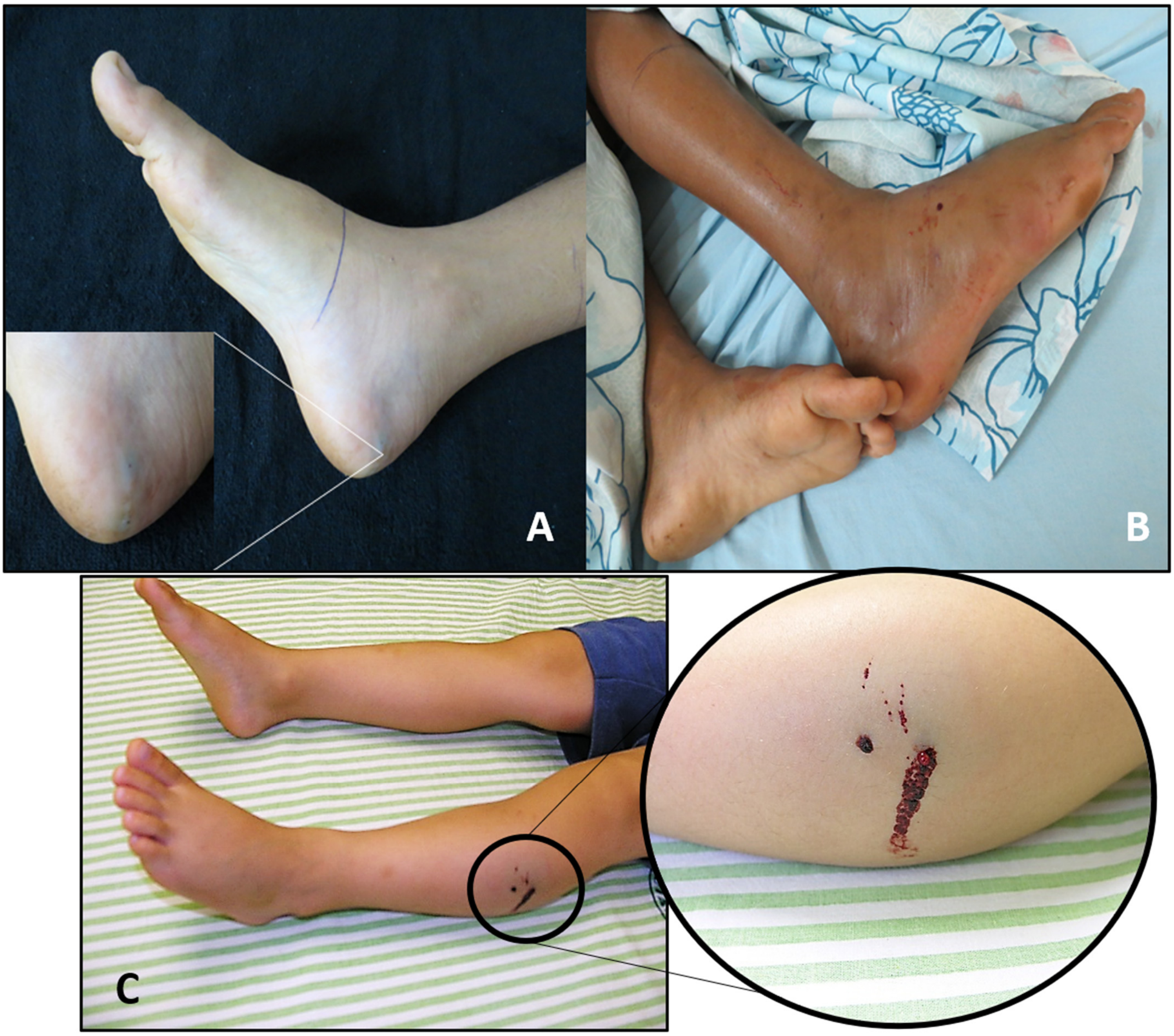

Figure 1 – (A) Punctiform lesion on the right heel; (B) Local signs with the back of the foot edematous and reddish, with bleeding at the site of the bite; (C) punctiform lesion on the left calf. *Images from the CEPCLAM/FMT-HVD files.*

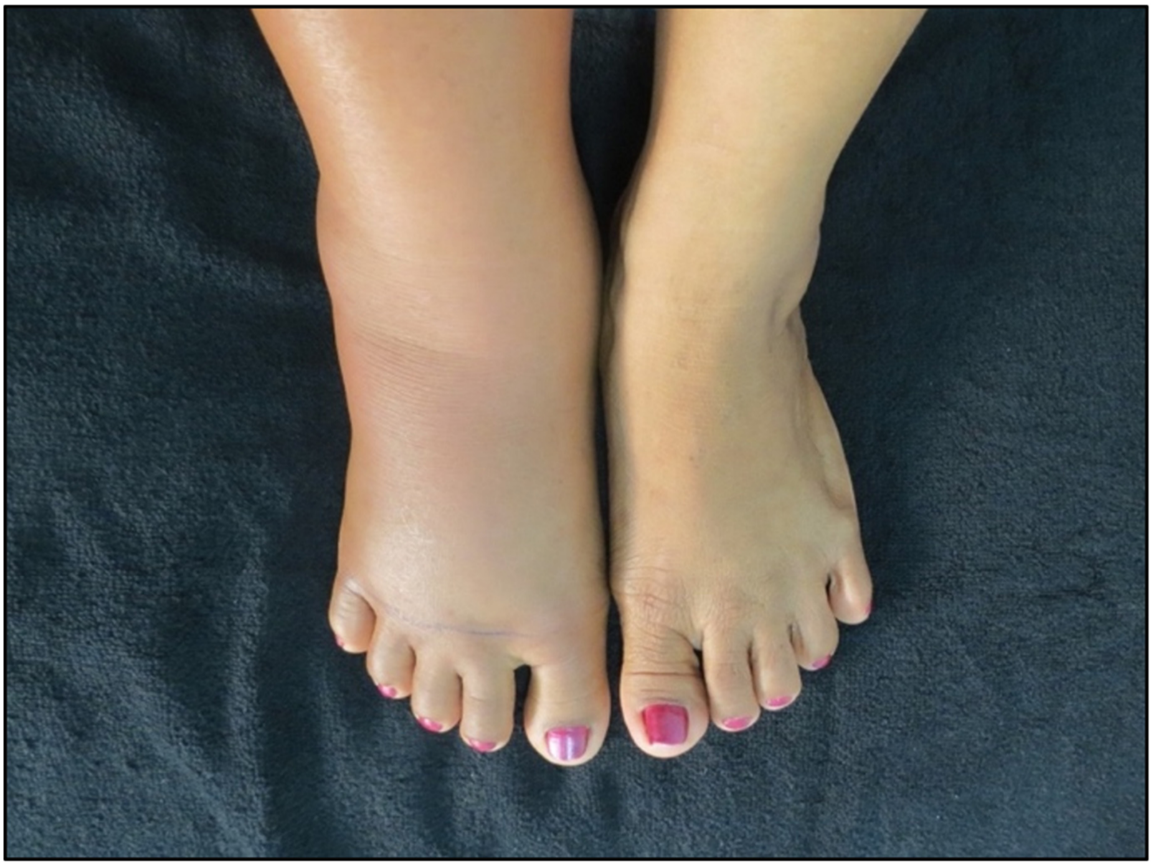

Figure 2 – Edema evident in the foot and leg, with slightly reddened skin, classic signs of a *Bothrops* bite. *Images from the CEPCLAM/FMT-HVD files*.

AO1

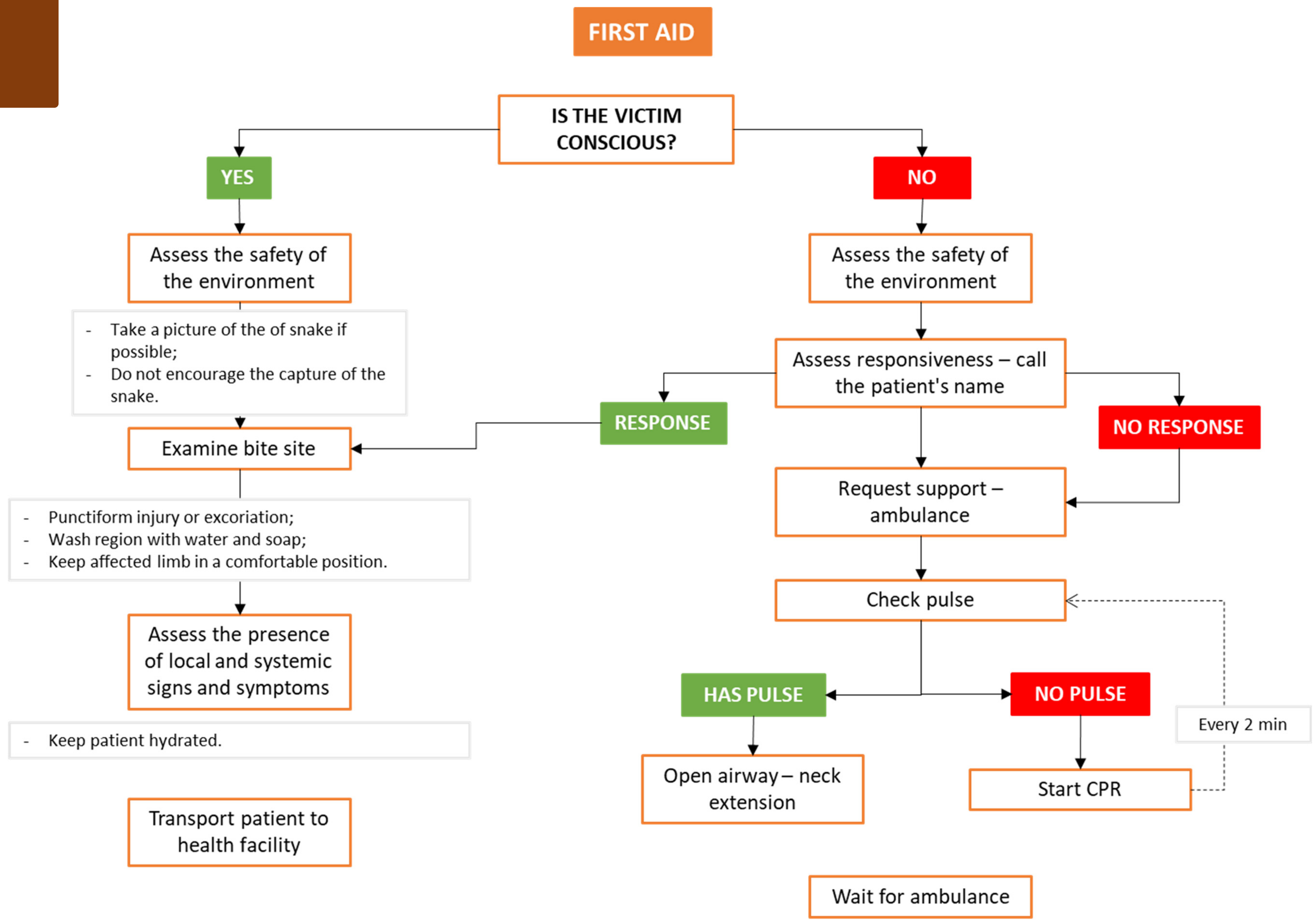

FIRST AID
IS THE VICTIM CONSCIOUS?
YES
NO
Assess the safety of the environment
- Take a picture of the of snake if possible;
- Do not encourage the capture of the snake.
Examine bite site
- Punctiform injury or excoriation;
- Wash region with water and soap;
- Keep affected limb in a comfortable position.
Assess the presence of local and systemic signs and symptoms
- Keep patient hydrated.
Transport patient to health facility
Assess the safety of the environment
Assess responsiveness – call the patient's name
RESPONSE
NO RESPONSE
Request support – ambulance
Check pulse
HAS PULSE
NO PULSE
Open airway – neck extension
Start CPR
Every 2 min
Wait for ambulance

AO2 – Diagnosis and clinical classification of the envenoming

The patient should be assessed for the presence of characteristic clinical signs and symptoms of envenoming, considering the epidemiology of the case and the description or identification of the causative agent.

Procedure:

- Evaluate signs and symptoms of envenoming according to clinical and epidemiological characteristics, as described below:

i. ***Bothrops* (common lancehead):** this corresponds to the snakebite with the biggest epidemiological burden in the country and is responsible for almost all cases in the state of Amazonas.

Bothrops envenoming is characterized by immediate **pain** at the site of the bite, and commonly presents with early onset of subsequent appearance of edema of varying intensity. Edema may be restricted to the site of the bite, but it is common to spread along the affected limb. **Bleeding** from the perforations caused by the bite is frequent. Ecchymoses may appear around the bite site or in other parts of the limb.

Bleeding may appear in preexisting skin injuries, or appear spontaneously, in the form of **gingivorrhagia, hematuria, conjunctival bleeding, epistaxis, hemoptysis and hematemesis**.

The patient's blood may be unclottable and this can be detected by clotting time test (CTT) (see AO3). CTT is not employed in the assessment of the severity of poisoning, but it is important that the test be performed at the time of admission for the evaluation of the patient evolution after antivenom administration.

Nausea, vomiting, sweating and headaches may occur.

Less common manifestations, observed in patients who are slow to receive treatment, are blisters on the affected limb with a liquid content that can vary in color, and which can be bloody; compartmental syndrome, when compression of the neurovascular bundle occurs due to the intensity of edema; secondary infection caused by contamination by bacteria present in the mouth of the snake or bacteria present in the patient's skin and even due to application of homemade substances, and skin and subcutaneous necrosis. **Oliguria may also occur.**

a) **Mild:**	The most common form of envenoming, which is characterized by mild or absence of **local pain** and **edema**, **discrete or absence of hemorrhagic manifestations. Normal or altered CTT.**
b) **Moderate:**	Characterized by **pain and evident edema that goes beyond the injured anatomical segment. Moderate envenomings may or may not be accompanied by local or systemic**

	**hemorrhagic alterations. Normal or altered CTT.**
c) **Severe:**	Characterized by **intense and extensive local edema**, which can affect the entire injured limb, usually accompanied by **severe pain and, eventually, the presence of blisters**. As a result of edema, signs of **compartment syndrome** may appear. Systemic manifestations, such as **arterial hypotension, shock, oligoanuria or intense hemorrhages**, define the case as severe, regardless of the local condition. **Normal or altered CTT.**

ii. ***Lachesis*** **(bushmaster):** because these are snakes that are encountered in closed forest areas, where their population density is low, bites by *Lachesis* are very rare.

The clinical picture is very similar to that described for bothrops envenomings, except for the possibility of the occurrence of **vagal syndrome (arterial hypotension, dizziness, darkening of vision, bradycardia, abdominal cramps and diarrhea).**

a) **Moderate:**	Local manifestations present – there may be bleeding without vagal manifestations. **Normal or altered CTT.**
b) **Severe:**	Intense local manifestations, heavy bleeding and/or vagal manifestations. **Normal or altered CTT.**

iii. ***Crotalus*** **(rattlesnake):** does not occur wide geographical coverage in the state of Amazonas, except for, predominantly, in the municipality of Humaitá. *Crotalus* only live in regions of open, stony fields and in areas of the Cerrado biome.

Local manifestations are less significant, differing from *Bothrops* and *Lachesis* snakebites. In this, **there is no pain**, or it can be of low intensity. There is **local or regional paresthesia**, which can persist for a variable amount of time, and may be accompanied by **mild edema** or erythema at the site of the bite.

Neurological manifestations arise within the first hours after the bite, and are evidenced by paralysis of muscle groups, with cranio-caudal progression, starting, in general, with single or bilateral **eyelid ptosis**, **alterations in pupillary diameter**, **inability to move the eyeball** (ophthalmoplegia), and there may be difficulty in accommodation (**blurred vision**) and/or **double vision** (diplopia). **The sagging of the muscles of the face can cause sialorrhea and difficulty swallowing, and may also present alteration of smell and taste**. Possible **generalized muscle pain** (myalgia) may appear due to injury of skeletal muscles. In addition, consequent release of myoglobin pigment in the blood and urine may appear together with urine of reddish color or a darker shade, and may even be brown (myoglobinuria).

The progression of muscle weakness can compromise the muscles of the rib cage and lead the patient to acute respiratory failure.

Alterations in clotting time test (CTT), with or without bleeding, occur less frequently.

a) **Mild:**	Characterized by the presence of **discrete neuroparalytic signs and symptoms, without myalgia, alterations in urine color or oliguria. Normal or altered CTT.**
b) **Moderate:**	Characterized by the presence of **evident neuroparalytic signs and symptoms, mild myalgia and urine may present altered coloration. Normal or altered CTT.**
c) **Severe:**	**Neurotoxic signs and symptoms** are **evident and intense, myalgia is intense, urine is dark, and there may be oliguria or anuria. Normal or altered CTT.**

iv. ***Micrurus* (coral snakes):** rarely registered.

Slight local pain, usually accompanied by **paresthesia**. Characterized by **progressive muscle paralysis**, **palpebral ptosis**, and other neurological signs, such as **blurred vision, ophthalmoplegia,** and **paralysis of facial muscles**. Associated with these manifestations, difficulties may arise in maintaining an upright position, **localized or generalized myalgia**, and difficulty swallowing. **Flaccid paralysis of the respiratory muscles** compromises ventilation, and there may be evolution to **acute respiratory failure and apnea**.

All ***cases of coral snakebite*** with clinical manifestations **must be considered potentially severe due to the risk of respiratory failure.**

- Dry bites: occur when a venomous snake does not inject poison at the time of the bite. In these cases, the **marks of the fangs** can be observed, but **without local or systemic signs of envenoming**, and **PATIENTS DO NOT NEED ANTIVENOM**.
- If the patient arrives for care without signs of envenoming, they must remain under observation for at least 6 hours after the bite. It is recommended that CTT be performed as soon as the patient is admitted and at the end of the observation period. If there are no clinical manifestations or changes in CTT, the patient may be discharged with the diagnosis of a dry bite.

Note:

- Envenomings present local and systemic conditions and the final clinical classification will be based on the worst condition of the two. Example: *Bothrops* snakebite patient with mild edema (**mild local condition**) and hematuria (**moderate systemic condition**) should have its final classification as **MODERATE**.

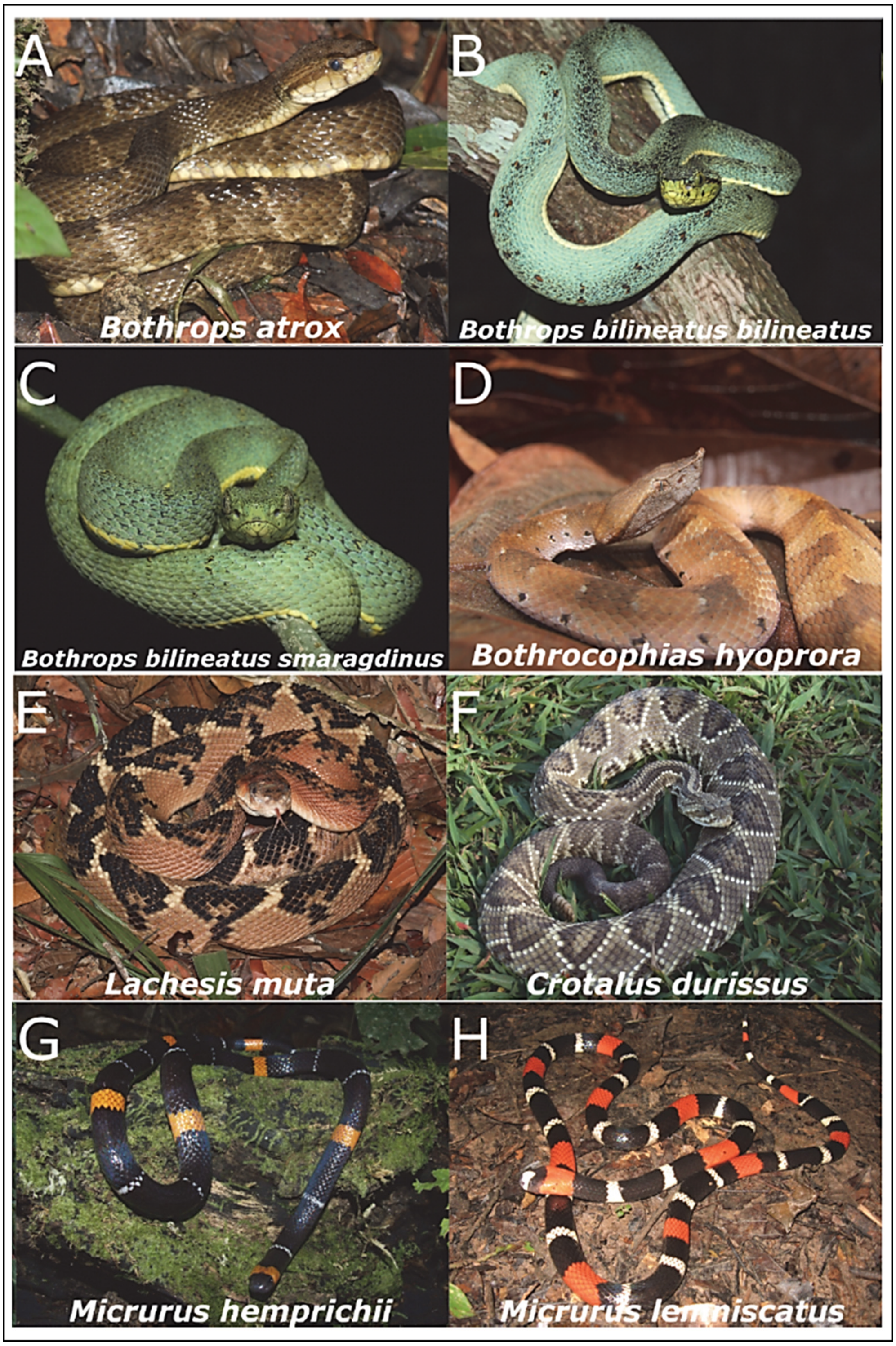

Figure 3 – Snake species involved in bites in the Brazilian Amazon. The images show the eight main snake species responsible for bites in the Brazilian Amazon region (A – H). *Bothrops Atrox* (A) is responsible for most snakebites recorded in the Brazilian Amazon region (80%-90%), followed by *Lachesis muta* (E). *Source: Hui Wen F, Monteiro WM, Moura da Silva AM, et al. (2015) Snakebites and Scorpion Stings*

in the Brazilian Amazon: Identifying Research Priorities for a Largely Neglected Problem. PLoS Negl Trop Dis 9(5): e0003701. doi:10.1371/journal.pntd.0003701.

DIAGNOSIS AND CLASSIFICATION

SIGNS AND SYMPTOMS

Bothrops – Lancehead

Mild
- Pain;
- Edema;
- Discreet bleeding;
- CTT normal or altered.

Moderate
- Pain;
- Evident edema (surpasses bitten segment);
- Local or systemic bleeding;
- CTT normal or altered.

Severe
- Intense pain;
- Intense or extensive local edema;
- Blisters;
- Compartiment syndrome;
- Arterial hypotension/shock;
- Oliguria/anuria;
- Intense bleeding;
- CTT normal or altered.

Lachesis – Bushmaster

Moderate
- Local manifestations present;
- Bleeding without vagal manifestations;
- CTT normal or altered.

Severe
- Intense local manifestations;
- Intense bleeding;
- Vagal manifestations.

Crotalus – Rattlesnake

Mild
- Mild neuroparalytic signs and symptoms;
- No myalgia;
- Oliguria/anuria;
- CTT normal or altered.

Moderate
- Obvious neuroparalytic signs and symptoms;
- Discreet myalgia;
- Urine with altered coloration;
- CTT normal or altered.

Severe
- Intense and evident neurotoxic signs and symptoms;
- Intense myalgia;
- Dark urine (with or without oliguria);
- CTT normal or altered.

Micrurus – True coral

Severe
- Pain;
- Paresthesia;
- Progressive muscle paralysis;
- Palpebral ptosis;
- Clouded vision;
- Ophthalmoplegia;
- Paralysis of facial muscles.

AO3 – Conducting the clotting time test (CTT)

The patient should be evaluated for the presence of alterations in coagulation time in the presence or absence of the characteristic clinical signs and symptoms of envenoming, considering the epidemiology of the case and the description or identification of the causative agent. For example, in mild cases of *Bothrops* bites, only the coagulation time may be altered.

Procedure:

1. Perform venipuncture with a plastic syringe and collect 4 ml of blood from the patient;
2. Add the 2 ml of blood in two equal diameter glass tubes (13x100 mm), down the wall of each tube;
3. Start the timer as you begin to pour the blood;
4. Put the glass tubes immediately into a water bath at 37 ºC;
5. After the 5th minute, and every 1 minute afterwards, perform the reading always using one of the glass tubes, by gently tilting it to 90 degrees to observe the coagulation of the blood. Keep the second glass tube at rest in the water bath, since it will be used as a control;
6. For this method, the values are:

Time taken for clot formation	Interpretation of the result
Up to 9 minutes	CTT **normal**
From 10 to 30 minutes	CTT **prolonged**
Over 30 minutes	CTT **unclottable**

7. After administration of antivenom, CTT examination should be performed after 12 hours.

Note: The coagulation time is **NOT** an examination to confirm envenoming, but an examination to assist in the evolution of the coagulation abnormality that *Bothrops* and *Lachesis* venom can cause. The patient with normal or altered coagulation time will always have the envenoming confirmed according to the clinical criterion.

- Complementary Laboratory Tests

When feasible, some laboratory tests should be performed at the time of the patient's admittance into the health unit and during the follow-up care (follow-up of the case), to aid the care as well as identification of possible injuries from the snakebite:

- Blood test;
- Urea and creatinine;
- Creatine Kinase (CK);
- Urinalysis;

- C-reactive protein.

CONDUCTING THE CLOTTING TIME EXAM - CT

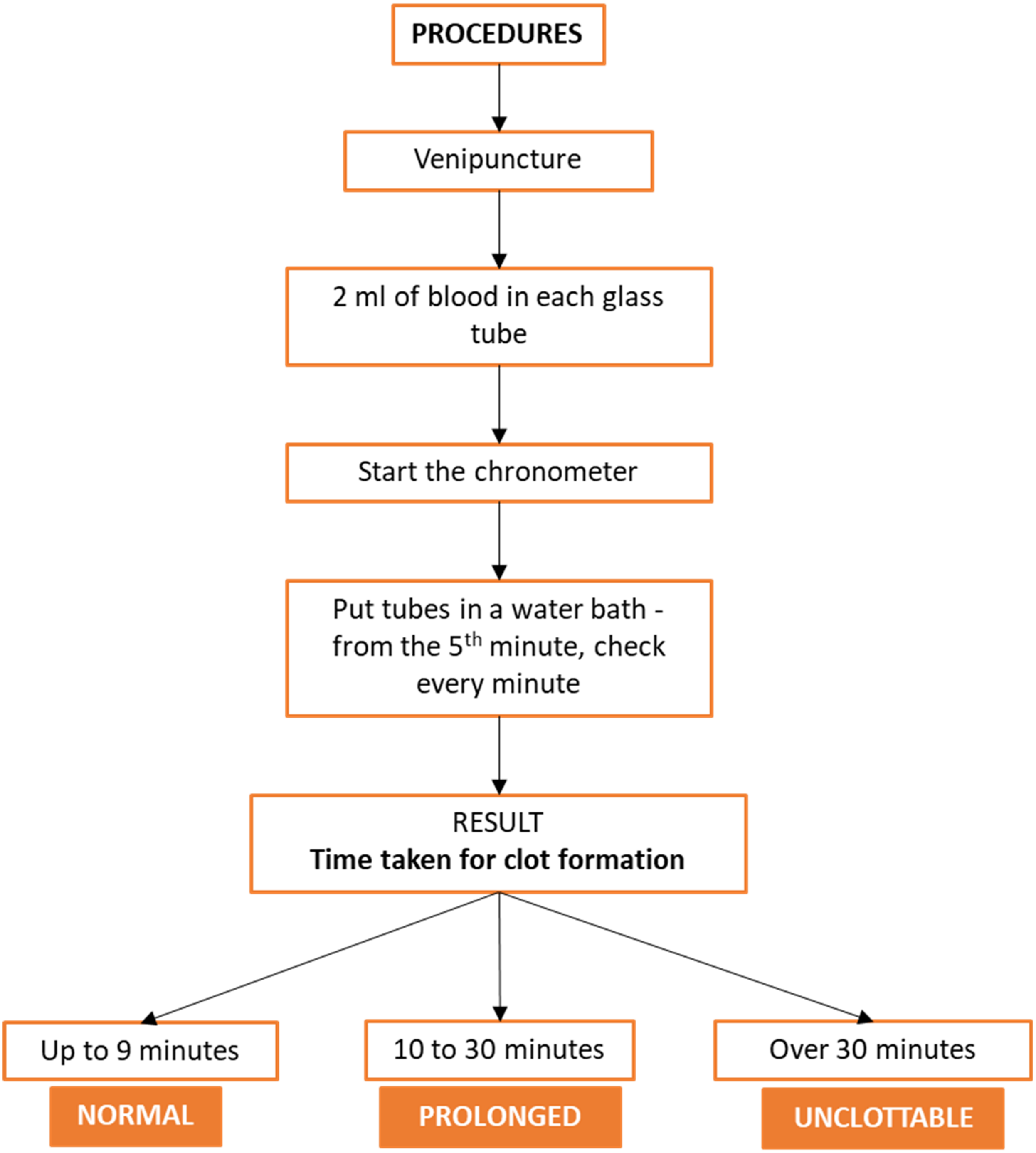

AO4 – Preparation of the antivenom for administration

The preparation of antivenom should be performed at the time of administration, i.e., after 30 min of pre-serotherapy, as described in AO5.

Procedure:

1. Check that the antivenom is stored under refrigeration between 2 ºC and 8 ºC or follow the guidelines on the package leaflet, where it contains the optimal storage temperature.

2. The antivenom should not be or have been frozen. See Figure 4 – the antivenom vial.

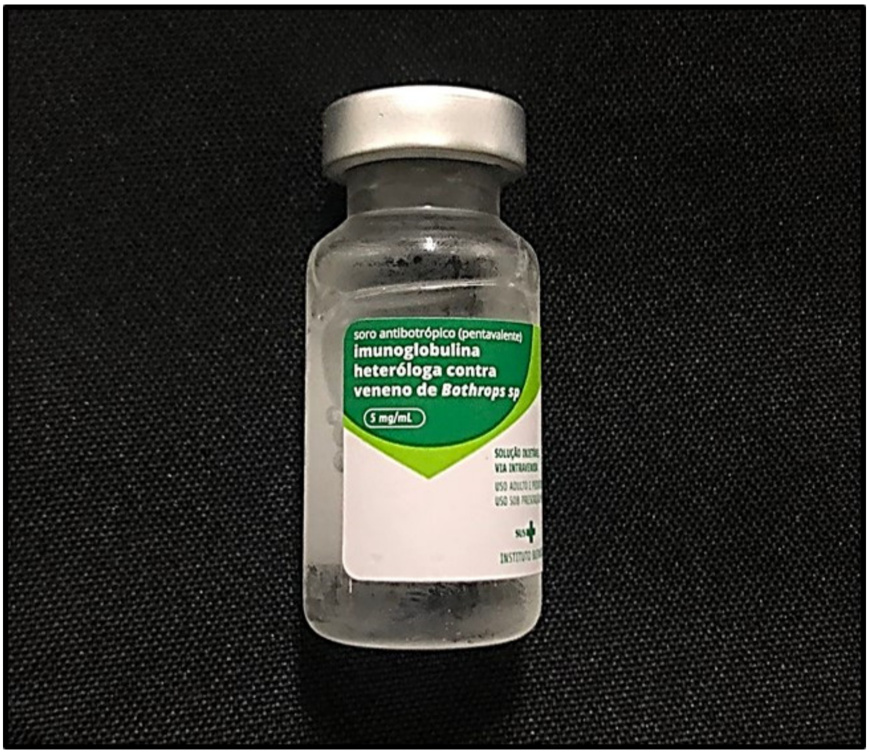

Figure 4 – Antivenom vial. *Images from the CEPCLAM/FMT-HVD files.*

3. Wash hands and put on surgical gloves.

4. Assemble the necessary material for the preparation of the antivenom: syringe, needle, cotton wool balls and antiseptic (alcohol 70%).

5. The batch number, date of manufacture, manufacturer's name and validity should be checked on the package (label and vial) and should be noted in the patient record. Do not use drugs with an expired shelf life.

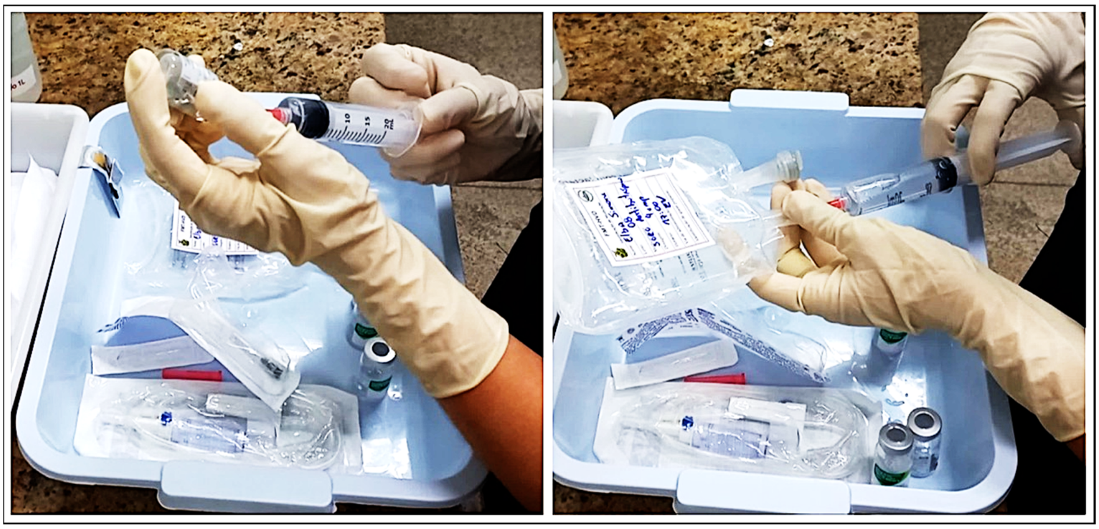

Figure 5 – Filling procedure of empty vials with antivenom for administration. *Images from the CEPCLAM/FMT-HVD files.*

6. Open an vial of glycosate or physiological solution and empty it so that only 100 ml of solution remains inside; open all vials of the prescribed antivenom and with the aid of a syringe, aspirate and place the antivenom inside a vial of antivenom containing the 100 ml of glycosate or physiological solution (Figure 5), according to the final volume of the antivenom with the solution. In Figure 6, note the materials that will be used.

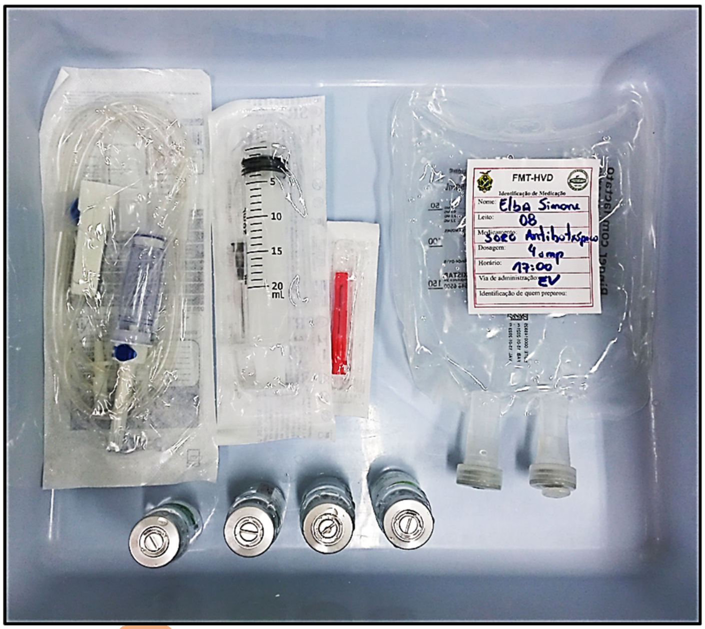

Figure 6 – Materials used for the administration of antivenom. *Images from the CEPCLAM/FMT-HVD files.*

7. After opening the vials, the antivenom should be administered immediately. Antivenom is a clear, colourless or slightly yellowish solution that should not contain lumps or particles. It should not be used if there is clouding (decreased transparency) or the presence of foreign material. See Figure 7 for the correct appearance of the antivenom.

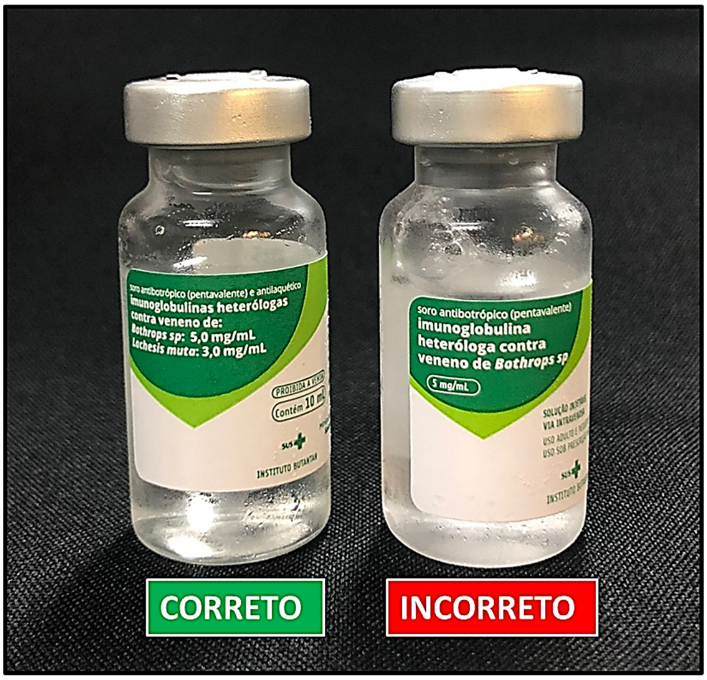

Figure 7 – Aspect of the antivenom.
Images from the CEPCLAM/FMT-HVD files.

8. If it is within the expiry date and you notice any change in appearance, consult the pharmacist to find out if it can be used.

9. The antivenom will be administered following the doses indicated by the doctor, **<u>in a slow infusion, between 20 and 60 minutes intravenously</u>** (IV) and the antivenom, will be **diluted or not in saline 0.9%.**

10. Antivenom can be administered at any time, even after meals or drinking alcoholic beverages, but it requires stricter care in these patients due to the risk of complications related to vomiting (aspirations).

11. The use of other medicines simultaneously with the antivenom does not compromise the treatment effects.

Note:

In case of expired or doubtful medication, follow the protocol of the National Immu[illegible] Program of the Secretary of Health.

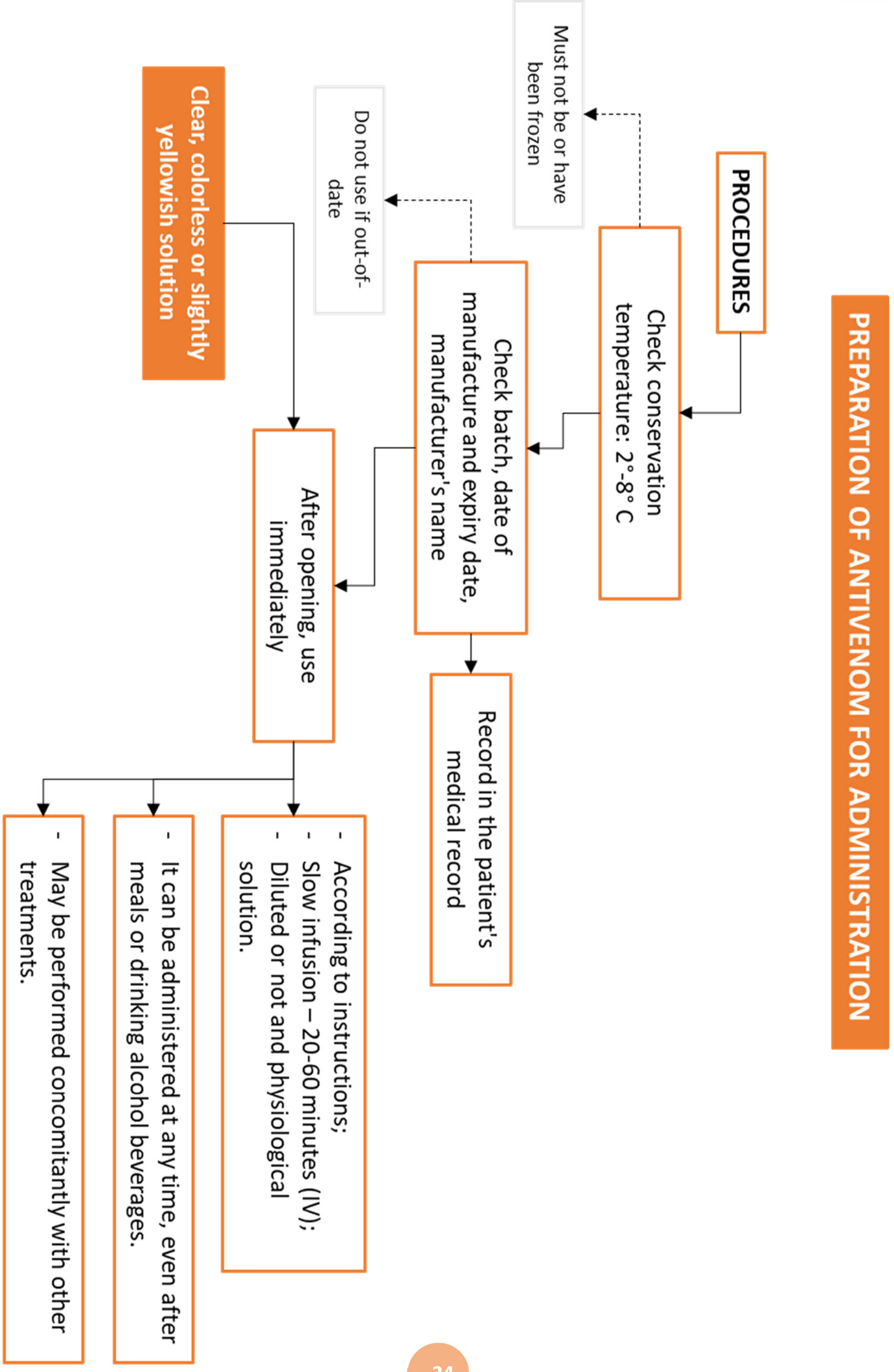

AO5 – Specific antivenom treatment

The choice of the type and dosage of antivenom is made according to the diagnosis and severity of the clinical picture, as previously described in AO2.

Procedure

1. **<u>Pre-serotherapy</u>**

- Obtain the patient's history of allergic conditions of any nature. If a history of allergies is present, increased attention should be required at the time of administration of the antivenom.
- Pre-serotherapy medication can be performed with some type of H1 receptor antagonist such as dexchlorpheniramine, loratadine and diphenhydramine.
- When oral drugs (dexchlorpheniramine and loratadine) are prescribed to prevent early adverse reactions, pre-serotherapy should be performed 30 minutes before antivenom administration.
- In the case of intravenous diphenhydramine, pre-serotherapy can be performed 5 to 10 minutes before antivenom administration.

Class	Medication	Dose
H1 receptor antagonists	Dexchlorpheniramine	0.05 mg/kg PO (children and adults)
	Loratadine	• Adult – 0.05 mg/kg PO;<br>• Children from 2 to 6 years – 5 mg PO (single dose);<br>• Children over 6 years – 10 mg PO (single dose);<br>• Children under 2 years – take dexchlorpheniramine
	Diphenhydramine	• Adult – 25 to 50 mg in adults (1 mL vial);<br>• Children over 2 years – 5 mg/kg/24 hours

- Despite the current lack of scientific evidence for pre-serotherapy, some services still combine H1 receptor antagonists with corticosteroids, such as hydrocortisone.

Class	Medication	Dose
Corticosteroid	Hydrocortisone	• Adults 300 mg;<br>• Children (or adults under 50 kg) 5 mg/kg

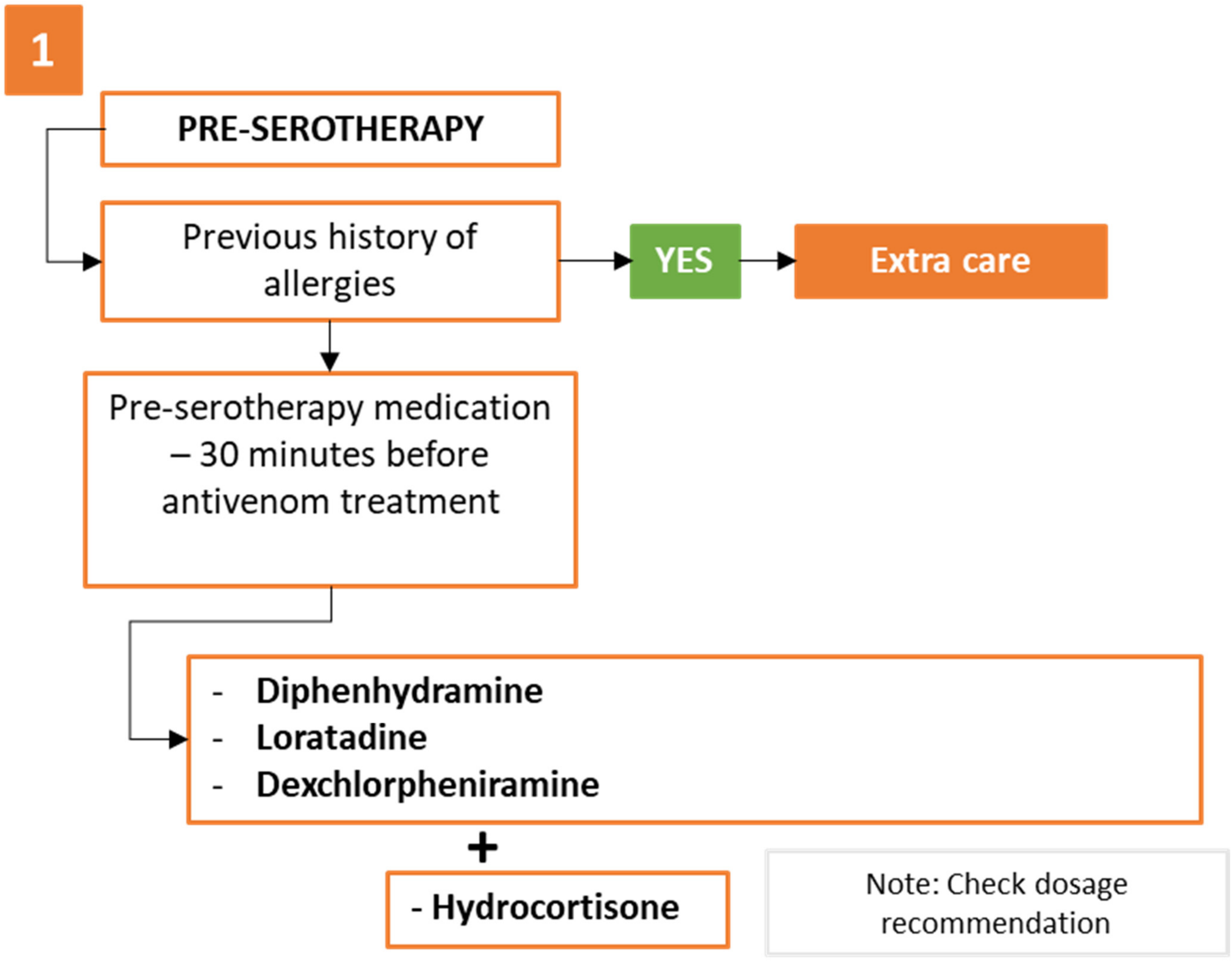
1
PRE-SEROTHERAPY
Previous history of allergies
YES
Extra care
Pre-serotherapy medication – 30 minutes before antivenom treatment
- Diphenhydramine
- Loratadine
- Dexchlorpheniramine
+
- Hydrocortisone
Note: Check dosage recommendation

2. <u>**Antivenom treatment**</u>

The following are the doses of antivenoms that should be administered for each type of envenoming.

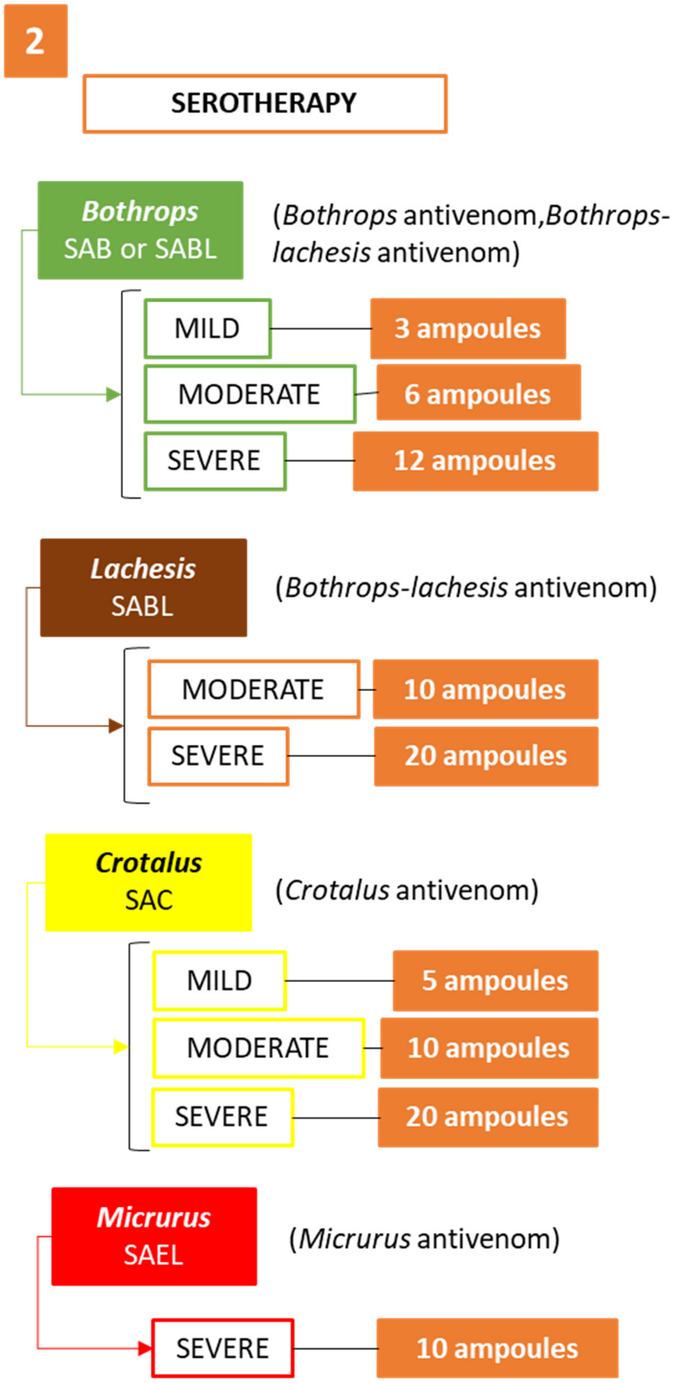

Note:

- The definition of the number of vials of antivenom to be administered is given solely according to the severity of the envenoming, and is irrespective of the site of the bite, sex, weight or age of the patient.

3. Administration of antivenom

For the prevention of adverse reactions, some specific care must be taken. The basic procedures for carrying out these activities are as follows:

a. Wash hands with soap and water.
b. Organize all material: syringe, needle and other materials necessary for the preventive treatment of anaphylaxis: adrenaline 1: 1,000, antihistamine, corticosteroid, oxygen, dextrose and saline solution, serum drip kit and material for ventilatory assistance.
c. Place intravenous access in the forearm and infuse 5% saline or dextrose, ensuring adequate venous access and maintenance of drip, even after administration of antivenom.
d. Control vital signs before starting antivenom administration.
e. Before administration of the antivenom, check: correct antivenom; correct dose; correct route; correct time; correct patient.
g. Start the drip slowly (15 to 20 drops per minute) of the prescribed antivenom in "y" with the saline or 5% dextrose previously placed in the patient's forearm.
h. The supervision of the nurse and the health team should be constant during the application of the antivenom with measurement of vital signs every 15 minutes, at least during the first hour.
i. The patient must have access via a thick vein. At the time of administration, they require bed rest with constant monitoring of signs for adverse reactions (pruritus, hoarseness, cutaneous rash, suffocation, increase in body temperature), and should be evaluated every 15 minutes, at least for the first hour.
j. Continuously observe the patient, for approximately two hours after administration of the antivenom. Venous access should be maintained with saline infusions, control of the drip, monitor vital signs and observe the coloration and temperature of the extremities (lips, fingers). Observe possible signs of restlessness, presence of pruritus, urticaria, conjunctival congestion, hoarseness, laryngeal stridor, dry cough, breathing noises, pallor, sweating, difficulty breathing, drop in blood pressure, weak pulse, among others.
k. In case of any adverse reaction, immediately stop the antivenom. Do not disregard the remaining antivenom unless contamination is suspected. Continue the drip of 5% saline or dextrose and start treatment with the recommended drugs:

1) Adrenaline: starting dose of 0.5 mg IM for adults, and 0.01 mg/kg weight for children, which can be repeated every 5-10 minutes if the reaction persists or if symptoms worsen. It is the first choice medication for treating allergic reactions.

2) Bronchodilator: if there are signs of wheezing, administer inhaled short-acting β2 agonist such as salbutamol or terbutaline, ideally by nebulization.

3) Antihistamine (anti-H1 Blocker): dextrochlorpheniramine maleate (adults 10 mg; children 0.2 mg/kg by IV injection).

4) Corticosteroides Hidrocortisona IV (adultos 100 mg, crianças 2 mg/kg).

l. As soon as the patient improves, restart the antivenom drip.
m. The patient should be kept under observation by the healthcare professional for at least 24 hours after antivenom administration.
n. Record the administration of the antivenom in the patient's medical record, recording: the amount and category of antivenom, time of administration, the visual appearance of the injury site, the duration of administration and the patient's tolerance to the procedure. Write down any adverse reaction.

Administration of concomitant treatments

Analgesia
- Assess pain intensity using the numerical scale.
- The analgesic should be prescribed immediately upon admission of the patient. In the first 24 hours, administration is usually systematic and should subsequently be evaluated for administration in the case of reports of pain.
- Oral or parenteral dipyrone, according to the pain scale and rest with limb elevated and in a comfortable position are measures that relieve the painful condition, until the edema regresses, except for compartment syndrome (in the first hours) or secondary infection (later, usually 48 hours after the bite). In the absence of dipyrone, other analgesics such as paracetamol or ibuprofen can be used.
- In the case of pain refractory to the analgesic treatments suggested above, the use of tramadol is recommended.

Note:
- Nonsteroidal anti-inflammatory drugs, similar to aspirin and diclofenac, are not recommended, due to the risk of kidney injury.
- Remember that the faster the patient receives the antivenom, the lower the chances of complications and sequelae from the envenoming.
- Concomitant treatment can be introduced according to the needs of the patient and the evaluation of the health professional that is attending the patient.
- There is no evidence that the use of antibiotics as an early therapy can prevent the emergence of a secondary infection.
- In the case of the administration of the antivenom in a primary care service that does not have a structure for monitoring the patient, refer the patient to the nearest hospital unit 1 hour after of the end of the administration of the antivenom.

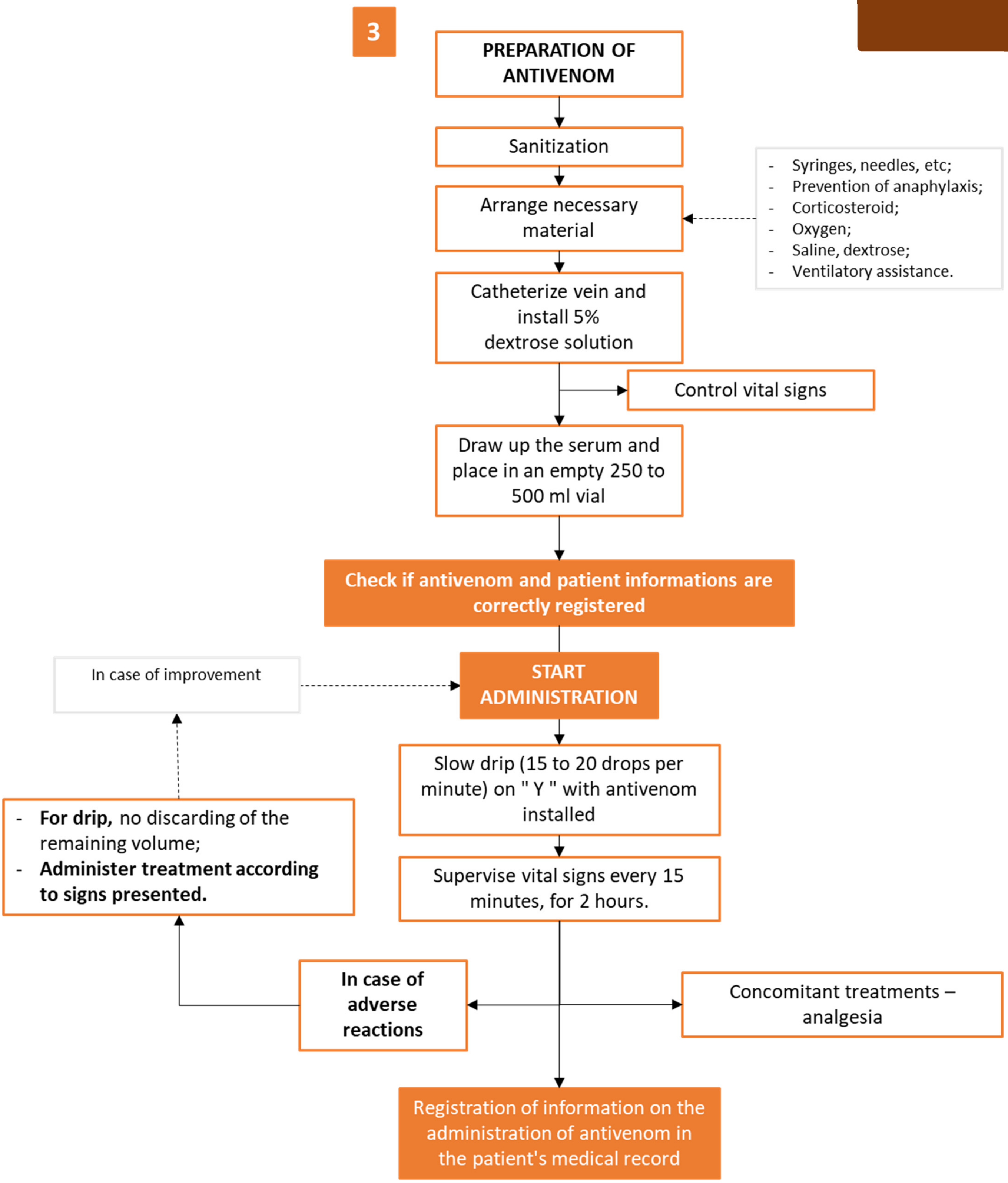
3
PREPARATION OF ANTIVENOM
Sanitization
Arrange necessary material
- Syringes, needles, etc;
- Prevention of anaphylaxis;
- Corticosteroid;
- Oxygen;
- Saline, dextrose;
- Ventilatory assistance.
Catheterize vein and install 5% dextrose solution
Control vital signs
Draw up the serum and place in an empty 250 to 500 ml vial
Check if antivenom and patient informations are correctly registered
In case of improvement
START ADMINISTRATION
Slow drip (15 to 20 drops per minute) on " Y " with antivenom installed
- For drip, no discarding of the remaining volume;
- Administer treatment according to signs presented.
Supervise vital signs every 15 minutes, for 2 hours.
In case of adverse reactions
Concomitant treatments – analgesia
Registration of information on the administration of antivenom in the patient's medical record

AO6 – Care of the bite site

The bite site should be evaluated daily to choose the best treatment.

Procedure:

1- Carry out cleaning of the bite site with saline, and in cases of bleeding, cover with sterile gauze and a loose bandage. If the lesion does not present with bleeding or exudate, no covering is required;

2- Evaluate the punctiform lesion and adjacent areas for the intensity of phlogistic signs (heat, flushing and pain);

3- Observe the evolution of the edema in the first hours to assess the need for reclassification of severity;

4- To assess the extent of the swelling, count the segments on the affected limb (segments of the upper limbs: hand, ½ distal of the forearm, and ½ proximal of the forearm, ½ distal of the arm, ½ proximal of the arm - total of 5 segments, and the segments of the lower limbs: feet, ½ distal of the leg, ½ proximal of the leg, ½ distal of the thigh, ½ proximal of the thigh). In cases of bites to other body parts, this classification does not apply;

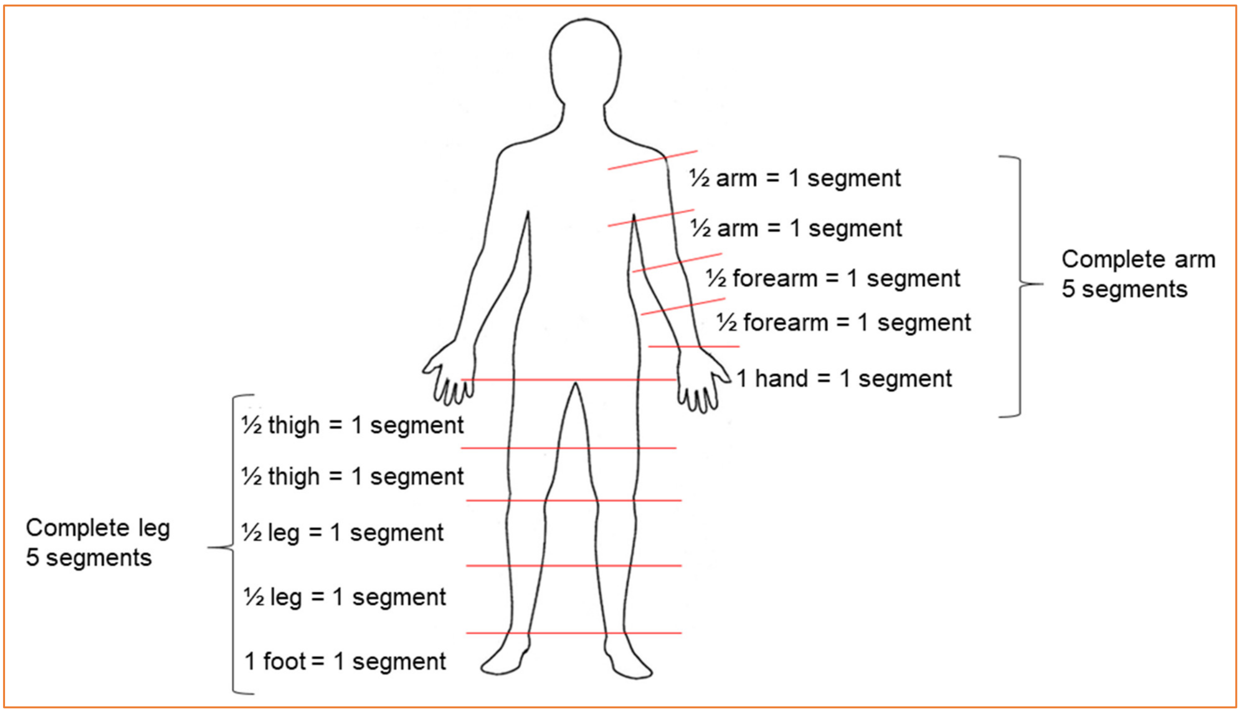

5- In cases of bites that occurred in places other than the lower and upper limbs, for example, to the trunk or head, the circumference should be measured at the site of the bite and the evolution should be compared with the measurements taken in previous days to observe improvement or worsening of edema due to the differences in centimeters;

6- Check daily for **signs of local severity** such as the **appearance of blisters** (Figures 8A and 8B). In these cases, the contents of the blisters may still contain poison that continues to act in a proteolytic way (causing tissue injury). These must be aspirated to remove the contents. After emptying the blister, cover the bite with gauze and perform daily dressings until complete healing of the injury;

7- Check daily for **signs of local bleeding**, such as ecchymosis in the affected limb or in regions more distal from the bite. Pay attention to the formation of accentuated blood blisters under the skin that can form blood clots and even cause infection. In these cases, these localized blood blisters should be aspirated or debrided. Cover the site with gauze and perform daily dressings until complete healing of the lesion (Figures 8E and 8F);

8- Check daily for the appearance of **signs of compartment syndrome** (Figure 3H) such as hardened edema in the entire limb, presenting intense, persistent and disproportionate pain. No response to the use of analgesics, causing a decrease or absence of distal pulses, cyanotic or bluish coloration of the skin. In these cases, there is a need to rapidly refer the patient for fasciotomy, since the compression of blood circulation in the limb can lead to tissue death and, subsequent amputation;

9- Check daily for **signs of infection** such as persistent localized pain, localized flushing, tissue fluctuation (softened tissue) and purulent discharge (Figures 8C and 8D). The appearance of fever may be indicative of secondary infection; performing ultrasound may be useful, when possible, to differentiate envenoming from secondary infection;

10- Check daily for **signs of necrosis** such as darkened coloration at the bite site or in nearby regions, decreased blood perfusion at the site (ischemia), mild to intense fetid odor, persistent localized severe pain (Figure 8G).

Note:
- Signs of complications at the site of the injury should be identified early in an attempt to avoid, or reduce the risk of functional loss of the affected limb or serious systemic repercussions that can lead to death.

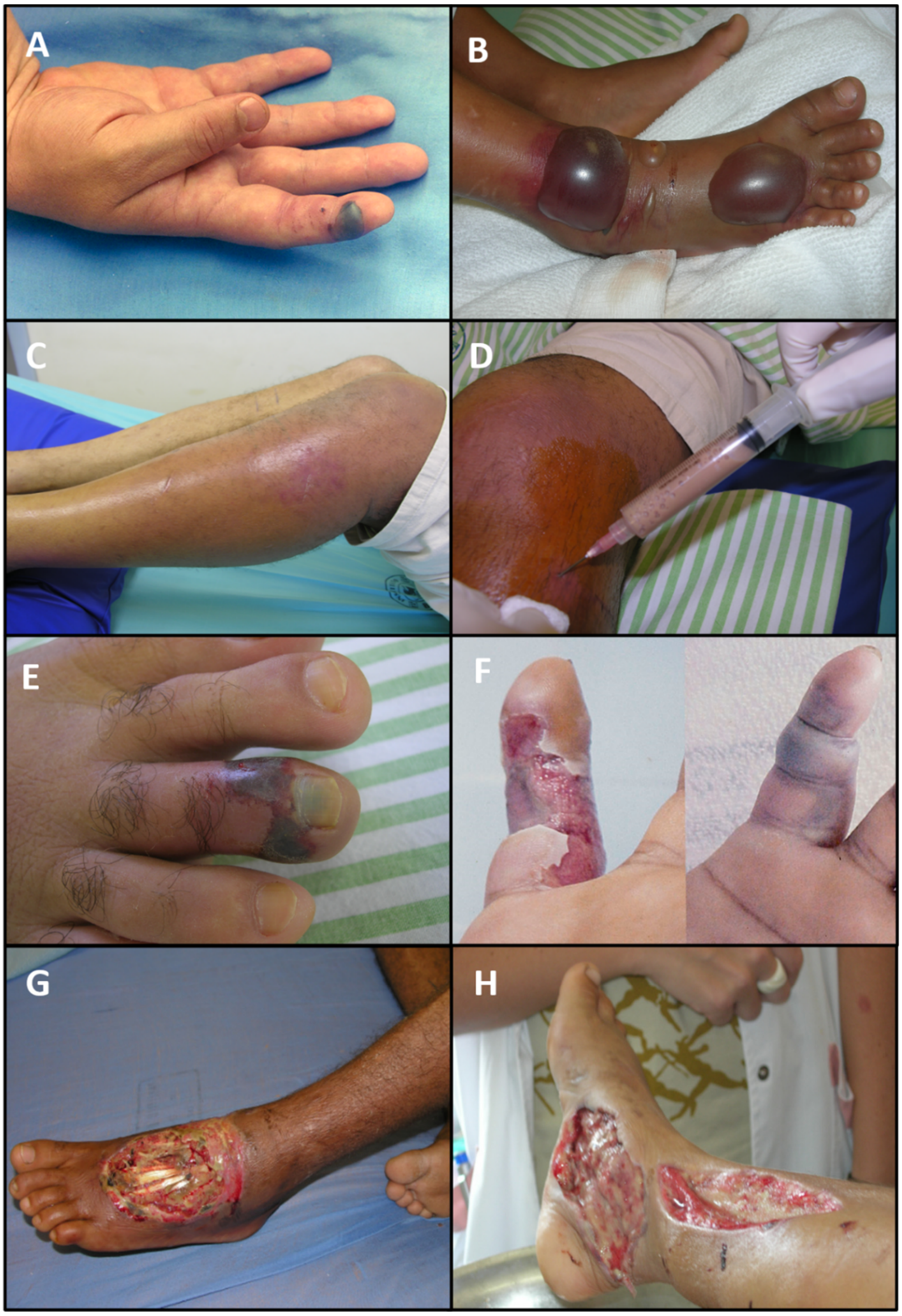

Figure 8 – (A) Blood blister on the index finger; (B) blood blister on the back of the right foot and ankle; (C) (D) secondary infection with cellulitis and abscess formation. (E) blood blister in second right pododactyl; (F) debridement in right little finger with granulation area and start of formation of

necrosis; (G) (H) formation of ulcer with fibrin and exposure of musculature after se[illegible] infection. *Images from the CEPCLAM/FMT-HVD files.*

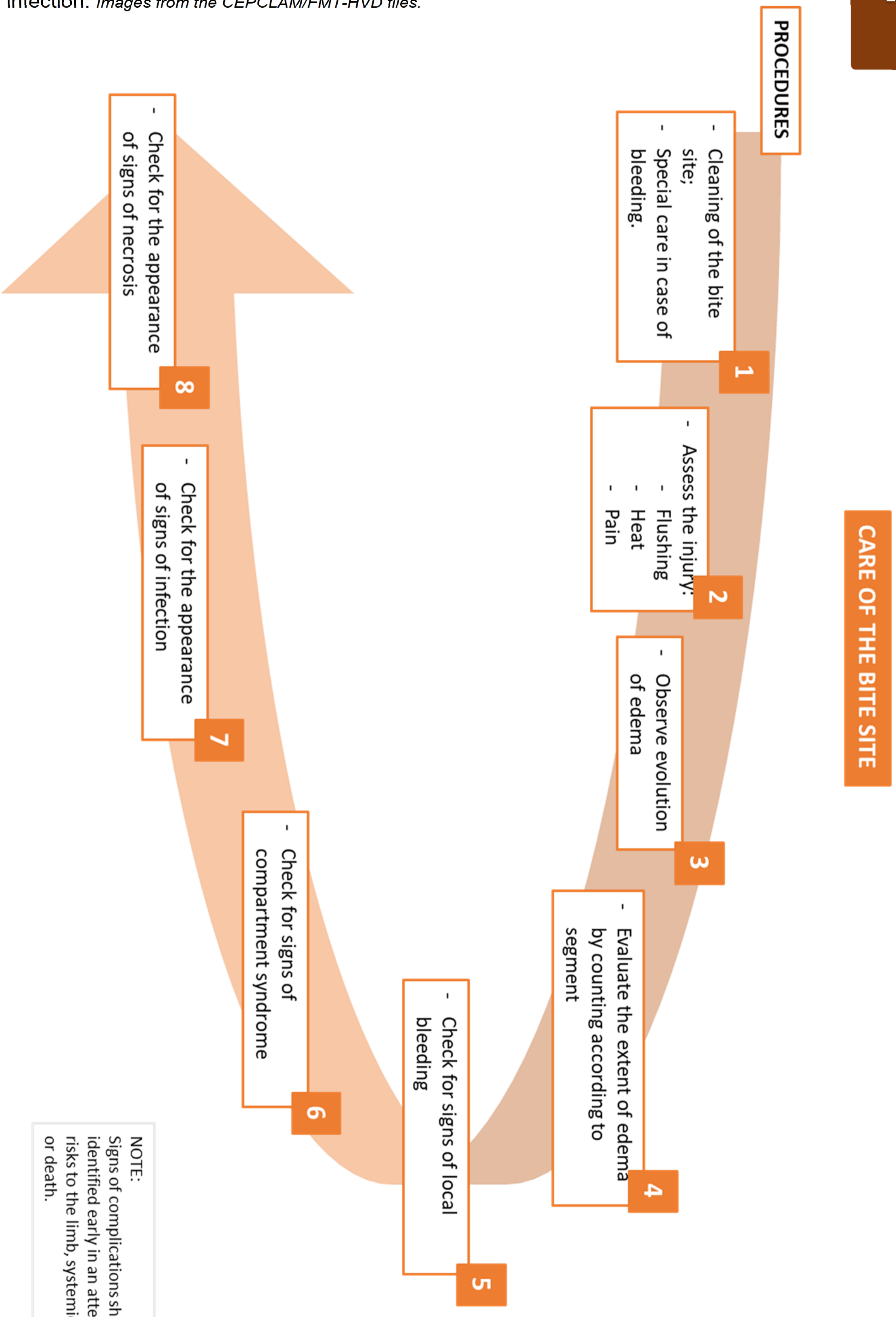

NOTE:
Signs of complications should be identified early in an attempt to reduce risks to the limb, systemic repercussions or death.

AO7 – Patient follow-up

The patient should be evaluated daily to identify improvement of their condition or discuss new procedures.

Procedure:

1. Initial assessment (first 12 hours)
   - After antivenom administration, the evaluation should be regarding the improvement of bleeding and coagulopathy and, eventually, the emergence of an early adverse reaction that is usually observed up to the first 2 hours.
   - Perform the CTT to accompany the evolution of coagulopathy every 12 hours after serotherapy until normalization, which should occur within 24 hours after administration of the antivenom;
   - Evaluate local clinical signs according to protocol AO1;
   - Continuously evaluate systemic signs of severity for bleeding, such as hematemesis, enterorrhagia, otorragia, macroscopic hematuria and, using the Glasgow Coma Scale for possible cerebral hemorrhage. It is expected that after the administration of antivenom, hemorrhagic manifestations will cease within a few hours.

2. Reclassification of the envenoming or extra antivenom doses
   - Criteria for re-classification:
     - Patient with clinical worsening of local symptomatology (rapid progression of edema) or systemic (persistence or appearance of hemorrhage, hypotension, shock) in the first 12 hours after administration of the antivenom;
     - Patient with unclottable blood after 12 hours: A patient that presents with prolonged CTT after 12 hours is in the process of clinical improvement and must be accompanied with a new CTT test in 24 to verify normalization of the test, or, if not, be reclassified.

   In such cases, the patient should be reclassified as to the severity of the snakebite and receive a new dosage of antivenom in the following situations:
   - The patient initially diagnosed as **MILD** may be reclassified to **MODERATE** and receive an additional 3 (three) vials of antivenom (total of 6 vials). It is expected that the patient will have improvement in coagulopathy in the first 24 hours after the first administration of the antivenom.
   - The patient initially diagnosed as **MILD** may be reclassified as **SEVERE** and receive an additional 9 (nine) vials of antivenom (total 12 vials) and be referred to the reference unit as quickly as possible.
   - Likewise, initially **MODERATE** cases can be reclassified as **SEVERE** and receive an additional 6 (six) vials of antivenom (total 12 vials) and be referred to the referral hospital as quickly as possible;

- In the case of the patient initially classified as **SEVERE**, the patient should receive the recommended dose according to the severity and be immediately referred to the referral hospital, and monitored by a health professional.

Note:

- At each additional administration of antivenom, the need for the patient to receive pre-serotherapy 30 minutes prior to the AO5 protocol should be evaluated. In cases where the initial serotherapy was performed a few hours before or when the risk due to the severity outweighs the risk of reaction, the possibility of administering the antivenom even without new premedication should be considered. On the other hand, if the patient has had a moderate or severe allergic reaction in the initial serotherapy, further administration should be considered with caution and premedication instituted.

PATIENT FOLLOW-UP

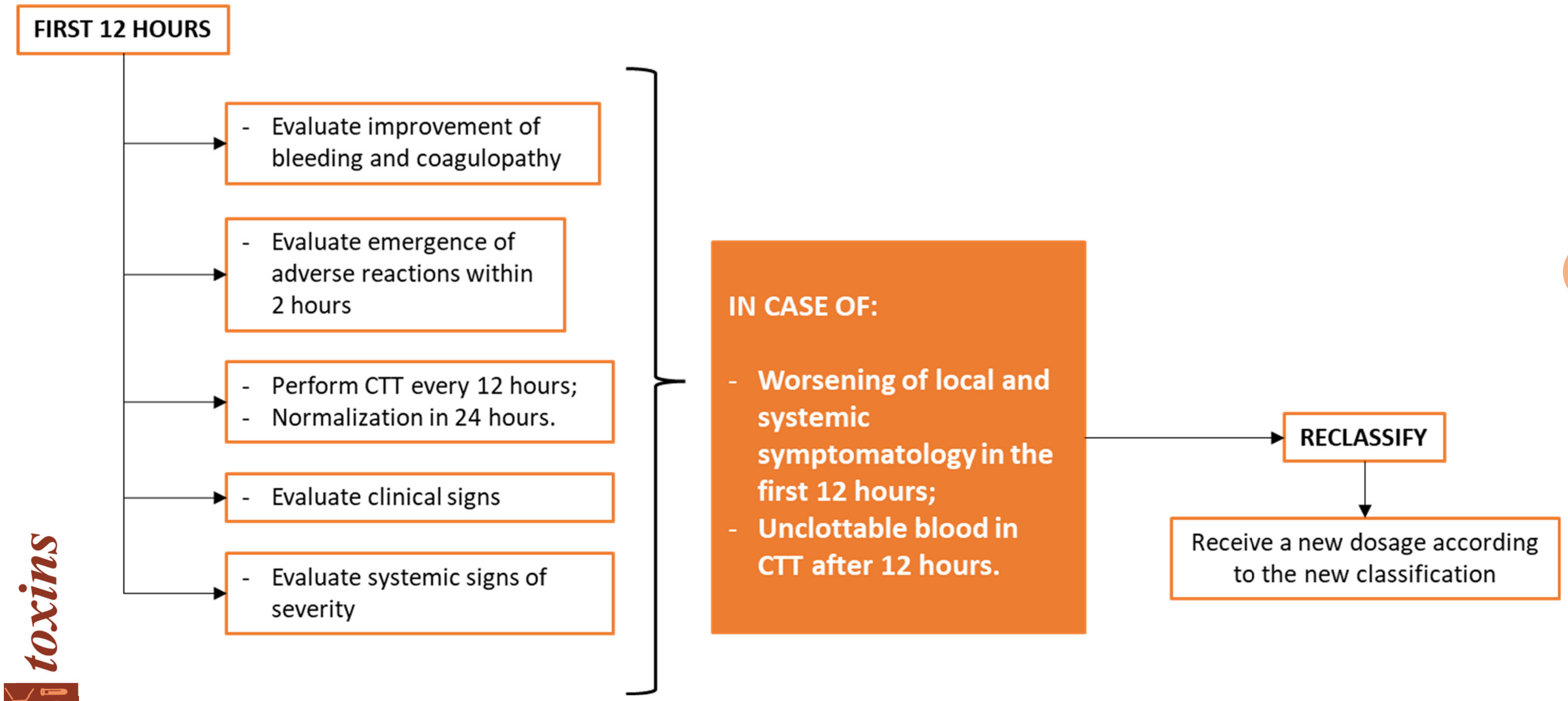

AO8 – Referral of the patient to a referral hospital

The patient should be constantly evaluated for identification of worsening of the condition or discussion of new procedures. In case of any sign of worsening, the patient should be immediately referred to the nearest referral hospital.

Procedure:

Referral criteria for referral hospital units are listed in the table below:

**Accident classified or reclassified as severe** (see AO2 and AO7)	- When the patient has severe edema suggestive of compartment syndrome;<br>- When there are indications of systemic bleeding that will compromise body volume and systems regulation;<br>- Development of acute kidney injury;<br>- Onset of coma with suspected hemorrhagic stroke.
**Development of signs of local severity** (see AO9)	- Signs of necrosis requiring amputation and debridement;<br>- Abscess formation with low antibiotic response for treatment of secondary infection.
**Adverse reaction to antivenom** (see AO4 and AO5)	Early adverse reaction: when any sign of immediate reaction occurs due to antivenom administration, such as pruritus, hoarseness, cutaneous rash, suffocation report, increased body temperature;<br>- Stop the infusion immediately and administer the drugs indicated in AO4 (adrenaline, bronchodilator, antihistamine and corticosteroids).

Note:

Referrals **MUST** follow the Regulation System (SISTER or SISREG).

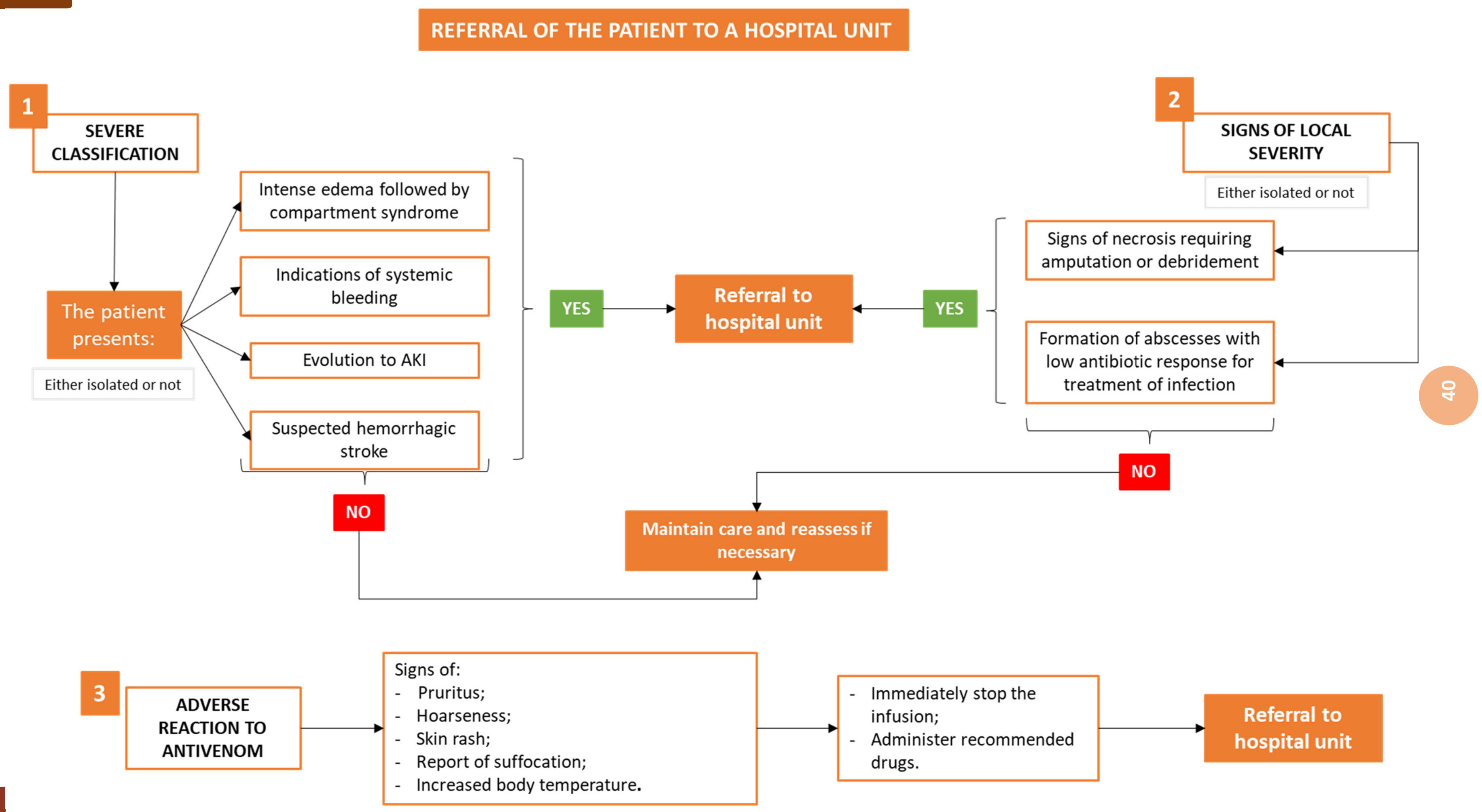
REFERRAL OF THE PATIENT TO A HOSPITAL UNIT
1
SEVERE CLASSIFICATION
The patient presents:
Either isolated or not
Intense edema followed by compartment syndrome
Indications of systemic bleeding
Evolution to AKI
Suspected hemorrhagic stroke
YES
Referral to hospital unit
YES
2
SIGNS OF LOCAL SEVERITY
Either isolated or not
Signs of necrosis requiring amputation or debridement
Formation of abscesses with low antibiotic response for treatment of infection
NO
NO
Maintain care and reassess if necessary
3
ADVERSE REACTION TO ANTIVENOM
Signs of:
- Pruritus;
- Hoarseness;
- Skin rash;
- Report of suffocation;
- Increased body temperature.
- Immediately stop the infusion;
- Administer recommended drugs.
Referral to hospital unit

AO9 – Evaluation of the complications of the snakebite

The patient should be evaluated daily to identify improvement of the condition or discuss new procedures. Complications usually show signs and symptoms starting 48 hours after envenoming.

Procedure:

1. The patient should be evaluated daily after the critical moment (first 48 hours) with focus on the appearance of complications arising from the snakebite with envenom.

2. Evaluation of the most prevalent envenoming complications, which are secondary infection, risk of functional loss of the affected limb due to extensive necrosis and acute kidney injury.

3. Clinical and laboratory evaluation is essential for early detection of complications and can reduce the damage to the patient.

4. Clinical Evaluation:

   - Acute kidney injury: consider AKIN classification criteria for acute kidney injury diagnosis and classification:

Stage	Serum Creatinine	Diuresis
Stage 1	0.3 mg/dl increase or 150-200% increase from baseline (1.5 to 2 times)	< 0.5 ml/Kg/h for 6 hours
Stage 2	> 200-300% increase from baseline (>2-3 times)	< 0.5 ml/Kg/h for > 12 hours
Stage 3	> 300% increase in baseline (> 3-fold or serum creatinine ≥ 4.0 mg/dl with an acute increase of at least 0.5 mg/dl)	< 0.3 ml/Kg/h for 24 hours or anuria for 12 hours

Only one of the criteria (Cr or diuresis) can be used for inclusion in the stage. Patients requiring dialysis are considered stage 3, regardless of the stage they were in at the beginning of dialysis therapy.

- **Secondary Infection:** local signs of severe and persistent pain, localized redness, cellulitis and abscess. In abscesses, antibiotic prescription and drainage of secretion should be performed. For systemic signs, watch out for fever, nausea, vomiting, lowering of the level of consciousness and sepsis. For antibiotic therapy, some of the options are recommended:

Drug	Dosage
Clindamycin	IV or PO
Amoxicillin + clavulanic acid	PO
Ceftriaxone + metronidazole	IV

5. Laboratory Evaluation:

Complication	Laboratory examination
Acute kidney injury	Creatinine<br>Urea
Secondary infection	Leukogram with differential

- Cases with a diagnosis of secondary infection, extensive necrosis requiring surgical procedure or acute kidney injury should be referred to the reference unit.

EVALUATION OF THE COMPLICATIONS OF THE SNAKEBITE

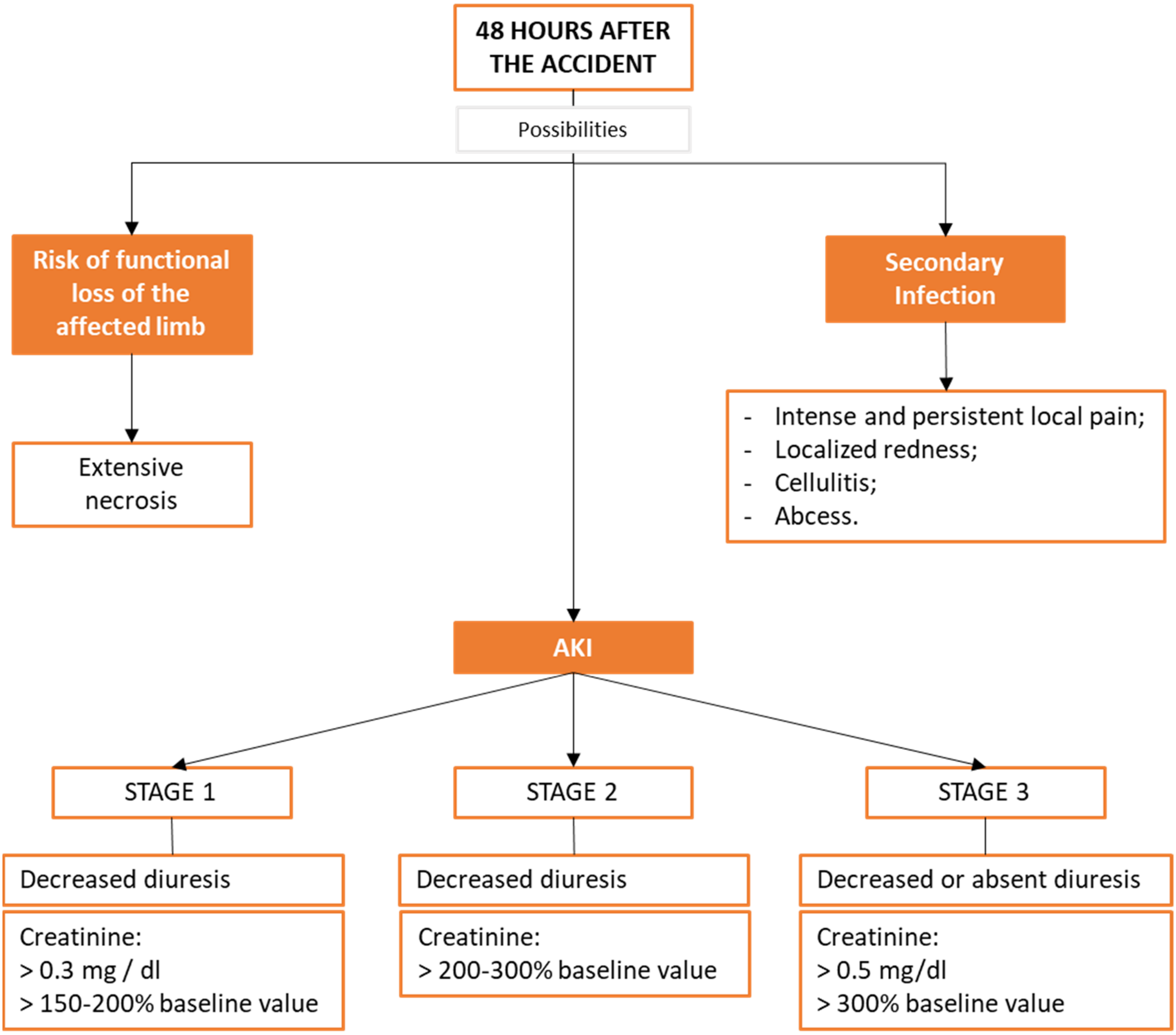

AO10 – Hospital discharge and outpatient follow-up

The patient should be evaluated daily to identify improvement of the condition, and definition of hospital discharge.

Procedure:

1. Post-discharge outpatient follow-up: at least 1 evaluation after hospital discharge should be scheduled with an interval of 7 (seven) days and should evaluate signs and symptoms at the site of the bite, such as necrosis, ulcer and infection, delayed reaction of antivenom (nodules, pruritus), report of decreased urinary volume, bleeding in skin and mucosa.

2. If necessary, perform physiotherapeutic follow-up for better restoration of mobility in the affected limb.

3. In the event that the lesion is open at the time of discharge, the patient should be directed to seek the nearest basic health unit for dressings and daily evaluations for wound care needs.

AO11 – Basic health unit discharge and follow-up

The patient should be evaluated daily to identify improvement of the condition and definition of discharge from the basic health unit.

Procedure:

1. Criteria for discharge from the basic health unit:
    a. Absence of signs of inflammation (pain, heat, flushing and edema), which allow the patient to walk independently.
    b. Patient does not present with signs raising suspicion of secondary infection such as the onset of cellulitis or abscess (see AO9).
    c. Patient no longer needs continuous medication such as pain relievers, antibiotics, etc.

AO12 – Filling out the notification form

The Venomous Animal Bite Notification form shall be completed in all cases of snakebites, venomous or not, with or without the use of antivenom.

Procedure:

1. Part 1 – sociodemographic data: fill in as much information as possible to identify patient characteristics

Federal Republic of Brazil
Ministry of Health

SINAN
INFORMATION SYSTEM OF NOTIFIABLE DISEASES
REPORT FORM
ACCIDENTS CAUSED BY VENOMOUS ANIMALS

Nº

CONFIRMED CASE: patient with clinical evidence of envenomation, specific to each type of animal, regardless of whether the animal that caused the accident was identified or not. There is no need to fill out the form for suspected cases.

General data

1 Type of notification — 2 - Individual

2 Problem/disease — ACCIDENTS CAUSED BY VENOMOUS ANIMALS — CODE (CID10) X 29

3 Notification date

4 State

5 Notifying municipality — Code (IBGE)

6 Health Uint (or other notifying source) — Code

7 Date of first symptoms

Individual data

8 Patient's name

9 Date of birth

10 (or)Age — 1 – time 2 – day 3 – month 4 - year

11 Sex — M = Male F = Female I = Ignored

12 Pregnancy — 1- 1st quarter 2- 2nd quarter - 3 - 3rd quarter 4 - gestational age ignored 5 - not pregnant 6 - not applicable 9 - ignored

13 Race/color — 1 – white 2 – black - 3 – yellow 4 – mixed 5 – indigenous 9- ignored

14 Education — 0 – illiterate 1 – 1st to 4th grade incomplete 2 4th grade complete 3 – 5th to 8th grade incomplete 4 - 8th grade complete 5 - High school incomplete 6 – High school complete 7 – University incomplete 8 – University complete 9 – ignored 10 – does not apply

15 SUS Health card number

16 Mother's name

Residency data

17 State

18 City/town of residence — Code (IBGE)

19 District

20 Neighborhood

21 Street/Ave — Code

22 Number

23 Complementary info (apt. house,...)

24 Geo field 1

25 Geo field 2

26 Reference point

27 ZIP

28 (XX) Telephone

29 Zone — 1 – urban 2 – Rural 3- periurban 9 – ignored

30 Country (if living outside Brazil)

2. Part 2 – identification of the bite: check in the patient's medical record for all the information recorded for the completion of this part. In cases where the professional has contact with the patient, the information in the form can be completed by interviewing and complementing the record in the medical record. Field number 46 should be registered if the snake is identified or the signs and symptoms are compatible with the type of snake.

Complementary Case Data

Epidemiological background

31 Investigation date
32 Occupation
33 Date of the accident
34 State
35 Municipality where accident occurred:
Code (IBGE)
36 Location where accident occurred:
37 Zone of occurrence
1- urban 2 – Rural 3 – periurban 9 - ignored
38 Time elapsed between accident and care
1) 0-1 h 2) 1-3 h 3) 3-6 h 4) 6-12 h 5) 12-24 h 6) 24 h + 9) ignored
39 Bite site
01- head 02- arm 03-forearm 04- hand 05- finger 06- trunk 07- thigh 08- leg 09- foot 10- toe 99- ignored

Clinical data

40 Local manifestations
1 - yes 2 - no 9 - ignored
41 If local manifestations exist, specify: 1 - yes 2 - no 9 - ignored
Pain Edema Bruising Necrosis Others (specify)
42 Systemic manifestations
1 - yes
2 - no
9 - ignored
43 If systemic manifestations exist, specify: 1 - yes 2 - no 9 - ignored
neuroparalytic (palpebral ptosis, blurred vision)
myolytic / hemolytic (myagalia, anemia, dark urine)
bleeding (gingivorrhagia, other bleeding)
Renal (oliguria/anuria
Vagal (vomiting, diarrhea)
Others (specify)
44 Clotting time
1 - Normal
2 - Altered
9 – Not done

Accident data

45 Type of accident
1 – snake 2 – spider 3 – scorpion 4 – lizard 5 – bee
6 – others ____________ 9 - ignored
46 Snake - type
1 – *Bothrops* 2 – *Crotalus* 3 – *Micurus* 4 – *Lachesis* 5 – non-venomous snake 9 - ignored
47 Spider type
1 – Phoneutria 2 – *Loxosceles* 3 – Latrodectus 4 – Other spider 9 – Ignored
48 Caterpillar type
1 – Lonomia 2 – Other caterpillar 9 – Ignored

3. Part 3 – Complete this part with as much information as possible and pay attention to the registration of the type of antivenom and the number of vials used. The evolution of the case should be recorded, as well as the complications that occurred with the patient.

Treatment

49 Case classification
1 – Mild 2 – Moderate 3 – Severe - 9 - Ignored
50 Serotherapy 1 - yes 2 - no 9 - ignored
51 If yes to serotherapy, how many ampoules?
Anti-*Bothrops* (SAB)
Anti-*Bothrops-Lachesis* (SABL)
Anti-*Bothrops-Crotalus* (SABC)
Anti-*Crotalus* (SAC)
Anti-*Micurus* (SAM)
Anti-scorpion (SAEs)
Anti-aracnid (SAAr)
Anti-*Loxosceles* (SALox)
Anti-*Lonomia* (SALon)
52 Local complications
1 - yes 2 - no 9 - ignored
53 Do local complications exist? 1 - yes 2 - no 9 - ignored
Secundary infection
Extensive necrosis
Compartment syndrome
Functional deficit
Amputation
54 Systemic complications
1 - yes 2 - no 9 - ignored
55 Do systemic complications exist? 1 - yes 2 - no 9 - ignored
Renal failure
Respiratory failure/acute pulmonary edema
Septicemia
Shock

Conclusion

56 Work-related accident?
1 - yes
2 - no
9 - ignored
57 Case evolution
1 – Cure 2- Death caused by venomous
3 – Death due to other causes 9 - Ignored
58 Date of death
59 Closing date

4. Part 4 – use this part of the form for a quick consultation of the effects of the venom on local and systemic signs.

Venomous animal accidents: clinical manifestations, classification and serotherapy

	Type	Clinical Manifestations	Serum type	No. of ampoules
SNAKES	*Bothrops*	Mild: pain, local edema and discreet bruising	SAB	2 - 4
		Moderate: evident pain, edema and bruising, discreet hemorrhagic manifestations		4 - 8
		Severe: severe and extensive pain and edema, blisters, heavy bleeding, oligonuria, hypotension		12
	Crotalus	Mild: palpebral ptosis. mild blurred vision of late appearance, no changes in urine color, mild or absent myalgia	SAC	5
		Moderate: palpebral ptosis, discreet blurred vision of early appearance, dark urine color, discreet myalgia		10
		Severe: palpebral ptosis, evident and intense blurred vision, intense and generalized myalgia, dark urine, oliguria or anuria		20
	Lachesis	Moderate: pain, edema, blisters and discreet hemorrhage	SABL	10
		Severe: pain, edema, blisters, hemorrhage, abdominal cramps, diarroea, bradycardia, hypotension		20
	Micurus	Severe: pain or mild parethesia, palpebral ptosis, blurred vision	SAEL	10
SCORPIONS	Scorpiones	Mild: pain, erythema and local paresthesia	SAEsc or SAA	---
		Moderate: sweating, nausea, occasional vomiting, tachycardia, agitation and mild high blood pressure		2 - 3
		Severe: profuse and incoherent vomiting, profuse sweating, prostration, bradycardia, acute pulmonary edema and shock		4 - 6
SPIDERS	*Loxosceles*	Mild: uncharacteristic lesion with no identified spider	SAA or SALox	---
		Moderate: suggestive lesion with bruising, pallor, erythema, and local endurate edema, headache, fever exanthema		5
		Severe: characteristic lesion, intravascular hemolysis		10
	Phoneutria	Mild: local pain	SAA	---
		Moderate: occasional sweating, occasional vomiting, agitation, arterial hypertension		2 - 4
		Severe: profuse sweating, frequent vomiting, priapism, acute pulmonary edema, arterial hypotension		5 - 10
CATERPILLAR		Mild: pain, erythema, regional adenomegaly, normal coagulation, no bleeding	SALon	---
		Moderate: alterations in coagulation, bleeding in skin and/or mucous membranes		5
		Severe: alteration in coagulation, bleeding in viscera, renal failure		10

Complementary information and observations

Note all information that could be considered importantbut are not on the form (ex: other clinical data, lab data, results of other exams and necroscopy, etc.)

Investigator			
	Municipality/Health Unit		Health Unit Code
	Name	Role	Signature

Venomous Animal SINAN Net SVS 19/01/2006

Note:

Even bites that did not require antivenom require the filling in of the notification form to assist in the national database and characterization of the types of snakes that cause bites in Brazil.

AO13 – Receiving and storing the antivenom

The receipt and storage of antivenom must be carefully carried out and recorded to ensure adequate treatment in places that have a cold chain network, i.e., in health establishments that dispense immunobiologicals such as antivenoms.

Procedure:

1. Receive and verify the integrity of antivenom packages and vials, as well as record the number received and the physical condition (integrity of the vial and internal liquid without precipitates/lumps) of the antivenom;
2. Check the temperature used in the transport and the temperature of the place where it will be stored (2 to 8 ^{o}C);
3. Plan the periodic ordering of antivenoms according to the demand as a result of local use;
4. Relocate antivenoms to other services when the quantity stored exceeds the local need or when the expiry date is close and there is no prospect of prior use;
5. Control access by unauthorized persons to storage areas so as not to interfere with storage quality;
6. Carry out a daily routine of recording and controlling temperature and/or humidity in the distribution, reception and inspection rooms and storage and control room, as well as internally in the storage refrigeration equipment;
7. Have a contingency plan for all refrigeration equipment in cases of lack of electricity or situations that may present potential risk;
8. Perform routine training of human resources for control and manipulation of antivenoms;
9. Properly dispose of antivenom residues according to the guidance by your distribution center.

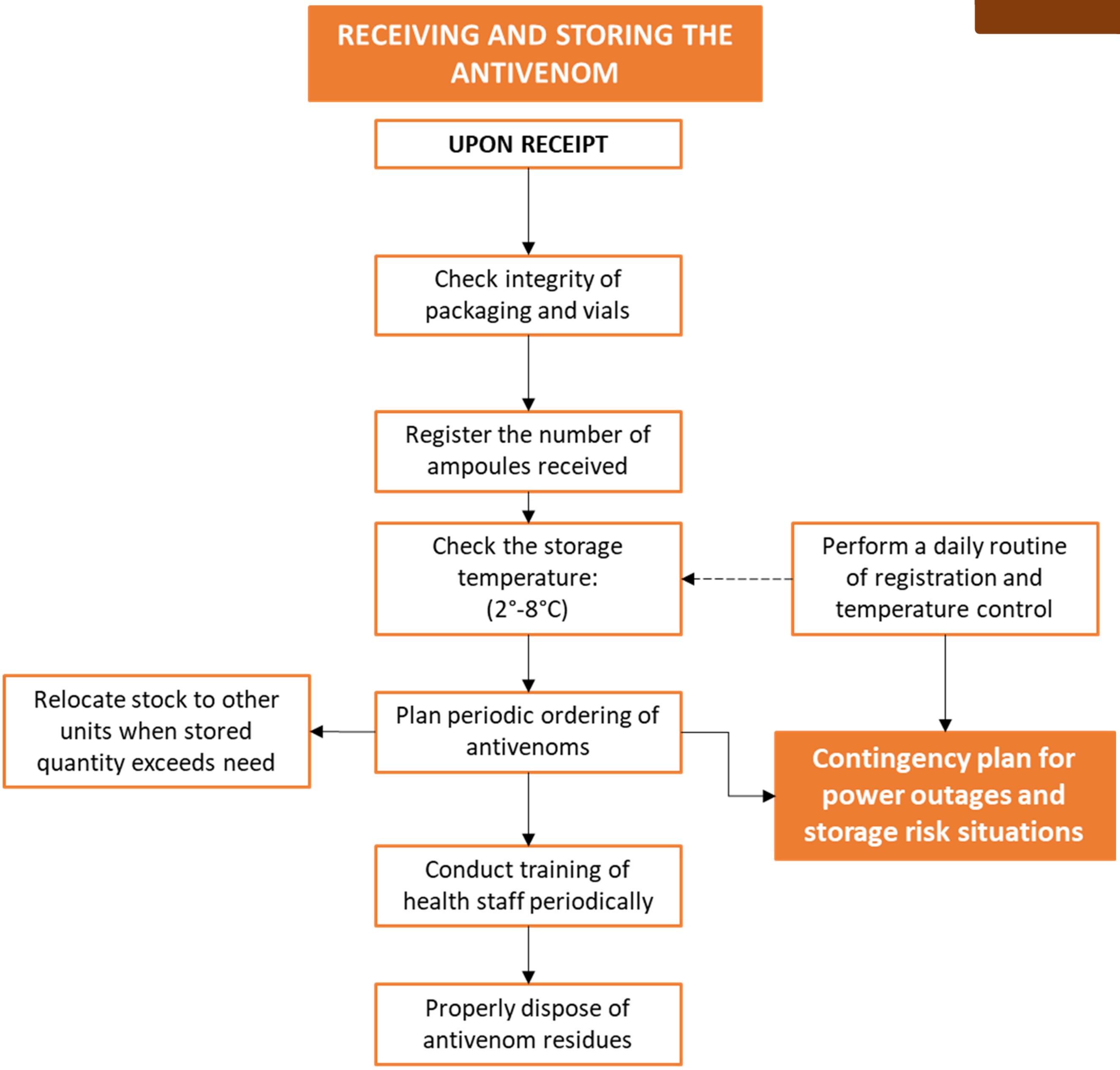
RECEIVING AND STORING THE ANTIVENOM
UPON RECEIPT
Check integrity of packaging and vials
Register the number of ampoules received
Check the storage temperature: (2°-8°C)
Perform a daily routine of registration and temperature control
Relocate stock to other units when stored quantity exceeds need
Plan periodic ordering of antivenoms
Contingency plan for power outages and storage risk situations
Conduct training of health staff periodically
Properly dispose of antivenom residues

Bibliographic references

AFONSO C; AZEVEDO GAM; ALVES P. Prevenção e tratamento de feridas: da evidência à prática. 1ª ed. Prior Velho: HARTMANN Portugal, 2014.

AHA. Adult Basic Life Support. 2020 International Consensus on Cardiopulmonary Resuscitation and Emergency Cardiovascular Care Science with Treatment Recommendations. Circulation. 2020;142:S41-S91.

ALBUQUERQUE PLMM, PAIVA JHHGL, MARTINS AMC, MENESES GC, SILVA GB, BUCKLEY N, ET AL. Clinical assessment and pathophysiology of *Bothrops* venom-related acute kidney injury: a scoping review. J Venom Anim Toxins Incl Trop Dis. 2020;26:e20190076.

BRASIL. MINISTÉRIO DA SAÚDE. Secretaria de Vigilância em Saúde. Coordenação-Geral de Desenvolvimento da Epidemiologia em Serviços. Capítulo 11 - Animais Peçonhentos. Ministério da Saúde: Brasília. Brasília. 5º ed. 2021.

BRASIL. MINISTÉRIO DA SAÚDE. Secretaria de Vigilância em Saúde. Departamento de Vigilância das Doenças Transmissíveis. Manual de Normas e Procedimentos para Vacinação. Ministério da Saúde: Brasília. Brasília, 2014.

BRASIL. MINISTÉRIO DA SAÚDE. Fundação Nacional de Saúde. Manual de Rede de Frio. 3. ed. Ministério da Saúde: Brasília. Brasília, 2001.

CARDOSO JLC; FRANÇA FOS; FAN HW; MALAQUE CMS; HADDAD JRV. Animais Peçonhentos no Brasil. Biologia, Clínica e Terapêutica dos Acidentes. 1. ed. São Paulo: Sarvier/Fapesp, 2003. v. 1. 468p .

CRISTINO JS; SALAZAR GM; MACHADO VA; HONORATO E; FARIAS AS; VISSOCI JRN; et al. A painful journey to antivenom: The therapeutic itinerary of snakebite patients in the Brazilian Amazon (The QUALISnake Study). PLoS Negl Trop Dis. 2021;15:e0009245.

FAN HW, MARCOPITO LF, CARDOSO JL, FRANÇA FO, MALAQUE CM, FERRARI RA, THEAKSTON RD, WARRELL DA. Sequential randomised and double blind trial of promethazine prophylaxis against early anaphylactic reactions to antivenom for *Bothrops* snake bites. BMJ. 1999;318:1451-2.

GIMENES SNC; SACHETT JAG; COLOMBINI M; FREITAS-DE-SOUSA LA; IBIAPINA HNS; COSTA AG; et al. Observation of *Bothrops atrox* Snake Envenoming Blister Formation from Five Patients: Pathophysiological Insights. Toxins. 2021;13:800.

INSTITUTO BUTANTAN. Soro antibotrópico (pentavalente) e anticrotálico. 2017. Disponível: http://butantan.gov.br/assets/pdf/soros_vacinas/soros/Bula-Soro-Antibotropico-Crotalico-Instituto-Butantan-Paciente-Consulta-Remedios.pdf.

JORGE MT; CARDOSO JL; CASTRO SC; RIBEIRO L; FRANÇA FO; ALMEIDA ME, et al. A randomized 'blinded' comparison of two doses of antivenom in the treatment of *Bothrops* envenoming in São Paulo, Brazil. Trans R Soc Trop Med Hyg. 1995;89:111-4.

JORGE MT; MALAQUE C; RIBEIRO LA; FAN HW; CARDOSO JL; NISHIOKA SA; et al. Failure of chloramphenicol prophylaxis to reduce the frequency of abscess formation as a complication of envenoming by *Bothrops* snakes in Brazil: a double-blind randomized controlled trial. Trans R Soc Trop Med Hyg. 2004;98:529-34.

JORGE MT, RIBEIRO LA, O'CONNELL JL. Prognostic factors for amputation in the case of envenoming by snakes of the *Bothrops* genus (Viperidae). Ann Trop Med Parasitol. 1999;93:401-8.

KELLUM JA; LAMEIRE N; ASPELIN P; BARSOUM RS; BURDMANN EA; GOLDSTEIN SL; et al. KDIGO Clinical Practice Guideline for Acute Kidney Injury. Kidney Int Suppl. 2012;2:124-38.

MONTEIRO WM; CONTRERAS-BERNAL JC; BISNETO PF; SACHETT J; MENDONÇA DA SILVA I; LACERDA M; et al. *Bothrops atrox*, the most important snake involved in human envenomings in the amazon: How venomics contributes to the knowledge of snake biology and clinical toxinology. Toxicon: X. 2020;1:100037.

OLIVEIRA SS; ALVES EC; SANTOS AS; NASCIMENTO EF; PEREIRA JPT; DA SILVA IM; et al. Snakebites in the Amazon: recovery from hemostatic disorders after Brazilian antivenom therapy. Clin Toxicol. 2019; 58:266-74.

OLIVEIRA S; ALVES E; SANTOS A; PEREIRA J; SARRAFF L; NASCIMENTO E; et al. Factors Associated with Systemic Bleeding in *Bothrops* Envenomation in a Tertiary Hospital in the Brazilian Amazon. Toxins. 2019;11:22.

PARDAL PP; SOUZA SM; MONTEIRO MR; FAN HW; CARDOSO JL; FRANÇA FO; et al. Clinical trial of two antivenoms for the treatment of *Bothrops* and *Lachesis* bites in the north eastern Amazon region of Brazil. Trans R Soc Trop Med Hyg. 2004;98:28-42.

POTTER PA; PERRY AG; ELKIN MK. Procedimentos e intervenções de enfermagem. 5ª ed. Rio de Janeiro: Elsevier, 2013.

PUCCA MB; KNUDSEN C; OLIVEIRA IS; RIMBAULT C; CERNI F; WEN FH; et al. Current Knowledge on Snake Dry Bites. Toxins. 2020;12:668.

RIBEIRO LA; JORGE MT; LEBRÃO ML. Prognostic factors for local necrosis in *Bothrops jararaca* (Brazilian pit viper) bites. Trans R Soc Trop Med Hyg. 2001;95:630-4.

SACHETT JAG; SILVA IM; ALVES EC; OLIVEIRA SS; SAMPAIO VS; VALE FF; et al. Poor efficacy of preemptive amoxicillin clavulanate for preventing secondary infection from *Bothrops* snakebites in the Brazilian Amazon: A randomized controlled clinical trial. PLoS Neg Trop Dis. 2017;11:e0005745.

SOUSA JDB; MARQUES HO; FAN HW; OLIVEIRA SS; LACERDA MVG; SACHETT JAG; et al. Accuracy of the Lee-White Clotting Time Performed in the Hospital Routine to Detect Coagulopathy in *Bothrops atrox* Envenomation. Am J Trop Med Hyg. 2018; 81:1547.

SOUZA AS; SACHETT JAG; ALCÂNTARA JA; FREIRE M; ALECRIM MGC; LACERDA M; et al. Snakebites as cause of deaths in the Western Brazilian Amazon: Why and who dies? Toxicon. 2018; 145:15-24.

STEVENS DL; BISNO AL; CHAMBERS HF; DELLINGER EP; GOLDSTEIN EJC; GORBACH SL; et al. Practice guidelines for the diagnosis and management of skin and soft tissue infections: 2014 update by the infectious diseases society of America. Clin Infect Dis. 2014;59:1-43.

toxins

MDPI

SAVING

Snake Antivenom Immunoglobulins

NEED TO BE GUARANTEED

FMT-HVD

UEA UNIVERSIDADE DO ESTADO DO AMAZONAS

MINISTÉRIO DA SAÚDE

Instituto de Pesquisa Clínica Carlos Borborema

CEPCLAM Centro de Pesquisa Clínica em Envenenamento por Animais

INSTITUTO BUTANTAN A serviço da vida

FVS FUNDAÇÃO DE VIGILÂNCIA EM SAÚDE DO AMAZONAS
