# Supplementary material for: Validation of a Culturally Relevant Snakebite Envenomation Clinical Practice Guideline in Brazil
Source: Toxins (Basel). 2022 May 28;14(6):376. doi: 10.3390/toxins14060376 (PMC9229773; doi:10.3390/toxins14060376)
Supplement: Supplementary file 1 [file toxins-14-00376-s001.zip › File S2.pdf]

toxins

MDPI

SAVING

Snake Antivenom Immunoglobulins

NEED TO BE GUARANTEED

Guia para o tratamento dos **acidentes ofídicos**

2022

toxins

MDPI

FICHA TÉCNICA

Autores

Jacqueline de Almeida Gonçalves Sachett
Fundação de Dermatologia Tropical e Venereologia Alfredo da Matta (FUAM)
Universidade do Estado do Amazonas (UEA)

Fan Hui Wen
Instituto Butantã (IBU)

Wuelton Marcelo Monteiro
Fundação de Medicina Tropical Dr. Heitor Vieira Dourado (FMT-HVD)
Universidade do Estado do Amazonas (UEA)

Colaboradores

Alícia Cacau Santos
Fundação de Medicina Tropical Dr. Heitor Vieira Dourado (FMT-HVD)
Universidade do Estado do Amazonas (UEA)

Altair Seabra Farias
Fundação de Medicina Tropical Dr. Heitor Vieira Dourado (FMT-HVD)
Universidade do Estado do Amazonas (UEA)

André Sachett
Fundação de Medicina Tropical Dr. Heitor Vieira Dourado (FMT-HVD)
Universidade do Estado do Amazonas (UEA)

Érica da Silva Carvalho
Fundação de Medicina Tropical Dr. Heitor Vieira Dourado (FMT-HVD)
Universidade do Estado do Amazonas (UEA)

Gisele dos Santos Rocha
Fundação de Medicina Tropical Dr. Heitor Vieira Dourado (FMT-HVD)
Universidade do Estado do Amazonas (UEA)

Joseir Saturnino Cristino
Fundação de Medicina Tropical Dr. Heitor Vieira Dourado (FMT-HVD)
Universidade do Estado do Amazonas (UEA)

Leonardo Marques
Fundação de Medicina Tropical Dr. Heitor Vieira Dourado (FMT-HVD)
Universidade do Estado do Amazonas (UEA)

Lisele Brasileiro
Fundação de Medicina Tropical Dr. Heitor Vieira Dourado (FMT-HVD)
Universidade do Estado do Amazonas (UEA)

Mena Bianca Ferreira
Fundação de Medicina Tropical Dr. Heitor Vieira Dourado (FMT-HVD)
Universidade do Estado do Amazonas (UEA)

Pedro Ferreira Bisneto
Universidade Federal do Amazonas (UFAM)

Vinícius Azevedo Machado
Universidade do Estado do Amazonas (UEA)

Yasmin Vieira Rocha
Fundação de Medicina Tropical Dr. Heitor Vieira Dourado (FMT-HVD)
Universidade do Estado do Amazonas (UEA)

Produção gráfica e diagramação

João Arthur Alcântara
Fundação de Medicina Tropical Dr. Heitor Vieira Dourado (FMT-HVD)
Universidade do Estado do Amazonas (UEA)

Revisão textual

Vinicius Milhomem
Universidade Federal do Amazonas (UFAM)

Validação

Ageane Mota da Silva
Instituto Federal do Acre (IFAC)

Aline Cristiane Côrtes de Alencar
Fundação de Medicina Tropical Dr. Heitor Vieira Dourado (FMT-HVD)

Ceila Maria Sant'Ana Málaque
Instituto Butantã (IBU)
Instituto de Infectologia Emílio Ribas

Eliane Campos Alves
Instituto Federal do Amazonas (IFAM)

Elielza Guerreiro Menezes
Universidade do Estado do Amazonas (UEA)

Flavio Santos Dourado
Ministério da Saúde (MS)

Giliard Santos da Costa
Hemonúcleo de Cruzeiro do Sul (AC)

Gisele Torrente
Universidade do Estado do Amazonas (UEA)

Guilherme Augusto Pivoto João
Fundação de Medicina Tropical Dr. Heitor Vieira Dourado (FMT-HVD)

toxins MDPI

Henry Maia Peixoto
Universidade de Brasília (UnB)

Iran Mendonça da Silva
Universidade do Estado do Amazonas (UEA)

Jaime Enrique Castro Valencia
Ministério da Saúde (MS)

Lúcia Regina Montebello Pereira
Ministério da Saúde (MS)

Márcia Almeida de Araújo Alexandre
Fundação de Medicina Tropical Dr. Heitor Vieira Dourado (FMT-HVD)

Maria Regina Fernandes de Oliveira
Universidade de Brasília (UnB)

Mariana Margarida Martinez Quiroga
Secretaria Municipal de Saúde de Santarém – PA (SEMSA)
Hospital Regional do Baixo Amazonas do Pará Dr. Waldemar Penna (HRBA)

Sâmella Silva de Oliveira
Hospital do Coração Francisca Mendes (HCFM)

Sandra Maria Deotti Carvalho
Ministério da Saúde (MS)
Secretaria de Estado da Saúde do Tocantins

Sílvio Cesar Pereira Fragoso
Fundação de Medicina Tropical Dr. Heitor Vieira Dourado (FMT-HVD)

Vanine de Lourdes Lima Fragoso
Fundação de Medicina Tropical Dr. Heitor Vieira Dourado (FMT-HVD)

Manaus/AM, 2022

Sachett, Jacqueline de Almeida Gonçalves
Guia para o tratamento dos acidentes ofídicos [livro eletrônico] / Jacqueline de Almeida Gonçalves Sachett, Fan Hui Wen, Wuelton Marcelo Monteiro. -- Manaus, AM : Ed. do Autor, 2022.
PDF.

ISBN 978-65-00-44339-4

1. Cobras venenosas - Brasil 2. Cobras venenosas - Brasil - Identitficação 3. Serpentes 4. Venenos - Efeitos fisiológicos 5. Venenos de origem animal I. Wen, Fan Hui. II. Monteiro, Wuelton Marcelo. III. Título.

22-109628 CDD-597.96

Índices para catálogo sistemático:

1. Cobras : Zoologia 597.96

Aline Graziele Benitez - Bibliotecária - CRB-1/3129

toxins MDPI

APRESENTAÇÃO

Para que as vítimas de envenenamentos ofídicos sejam tratadas com sucesso e se recuperem completamente, elas precisam ter acesso aos antivenenos e a outros procedimentos de cuidado. Este guia foi criado a partir da inquietude de pesquisadores e profissionais da assistência que atuam no campo da pesquisa clínica e cuidado de pacientes com envenenamentos ofídicos no Brasil. O problema identificado, durante a experiência clínica e científica, estava diretamente relacionado ao processo de trabalho dos profissionais de saúde, que têm escassos conhecimentos para a gestão do atendimento a estes pacientes e se sentem inseguros no durante seu manejo. Nesse sentido, um grupo de especialistas (dois da *Fundação de Medicina Tropical Dr. Heitor Vieira Dourado* – FMT-HVD, um hospital terciário de referência para o tratamento de envenenamentos ofídicos na Amazônia Brasileira, e um do *Instituto Butantan* - IBU, o maior produtor de antivenenos no país, que também trata casos de ofidismo na cidade de São Paulo) foi responsável pela criação da primeira versão de um guia para o manejo dos envenenamentos ofídicos, coordenando posteriormente sua validação de conteúdo e semântica.

O Ministério da Saúde do Brasil possui uma diretriz oficial com recomendações gerais para o manejo dos envenenamentos ofídicos e por outros animais peçonhentos. Esta diretriz apresenta os aspectos epidemiológicos e clínicos gerais dos envenenamentos pelos quatro gêneros de serpentes de importância médica no país (*Bothrops*, *Lachesis*, *Crotalus* e *Micrurus*), bem como os antivenenos disponíveis e suas dosagens de acordo com a gravidade do caso. A diretriz brasileira foi criada com base na opinião de especialistas considerando a experiência com pacientes atendidos em hospitais, sob supervisão médica. A diretriz não aborda atendimento pré-hospitalar e primeiros socorros; armazenamento, preparação e administração dos antivenenos; tratamento das feridas; tratamento auxiliar de diferentes manifestações locais e sistêmicas; encaminhamento para serviços de saúde de maior complexidade; critérios de alta; determinação do tempo de coagulação; e notificação dos casos aos sistemas de vigilância epidemiológica.

Ressalta-se que muitos destes procedimentos podem ser realizados pela equipe paramédica. Neste guia, também estão incluídas informações detalhadas sobre o manejo dos casos em unidades básicas de saúde, dependendo das políticas locais para tratamento de acidentes ofídicos e planos futuros para descentralização do tratamento antiofídico na Amazônia brasileira. A posologia dos antivenenos foi apresentada seguindo as mesmas recomendações do Ministério da Saúde, uma vez que estudos clínicos demonstraram que as dosagens dos antivenenos padrão são eficazes em reverter os efeitos sistêmicos do envenenamento.

Pesquisadores atuando na linha de envenenamentos ofídicos e profissionais de saúde experientes no atendimento deste agravo foram convidados a participar como juízes especialistas deste estudo, a fim de realizar a validação de conteúdo do guia. Os juízes estavam afiliados a instituições do Acre, Amazonas, Tocantins, Distrito Federal e São Paulo, garantindo uma amostra cultural e geograficamente diversificada.

Após essa etapa, foram consideradas as sugestões dos juízes e assim foi gerada a segunda versão do guia, que seguiu para a avaliação semântica por profissionais da rede básica de saúde dos municípios Careiro da Várzea, Ipixuna e Boa Vista do Ramo, no

Estado do Amazonas. Após as considerações dos profissionais, foi elaborada a versão final do guia.

Os responsáveis por este estudo agradecem a toda a equipe responsável pela pesquisa e a todos os juízes que participaram da validação de conteúdo e profissionais de saúde que permitiram a validação semântica deste guia. Destaca-se o apoio das secretarias de saúde dos municípios que liberaram seus profissionais a fim de receber treinamento e participarem do processo de validação em Manaus. Agradecemos ao apoio do Diretor Presidente da FMT-HVD, Dr. Marcus Vinitius de Farias Guerra, e ao Diretor de Assistência Médica desta instituição, Dr. Antônio Magela, que facilitaram a realização de todas as etapas do projeto. O estudo foi aprovado pelo Comitê de Ética em Pesquisa da Universidade do Estado do Amazonas (CAAE: 35855820.2.0000.5016). os autores agradecem ainda o financiamento concedido pelo Ministério da Saúde (733781/19-035), pela Fundação de Amparo à Pesquisa do Estado do Amazonas (PRÓ-ESTADO - 011/2021, PCGP/FAPEAM - 010/2021 e CT&I ÁREAS PRIORITÁRIAS) e pelo Fogarty International Center do National Institutes of Health (R21TW011944). O conteúdo deste guia é de responsabilidade exclusiva dos autores e não representa necessariamente as opiniões oficiais dos financiadores.

Este guia validado poderá ser integrado aos currículos dos profissionais de saúde em todos os níveis, a fim de melhorar a qualidade da atenção nos envenenamentos ofídicos e aumentar seu conhecimento e competência clínica na rotina. Finalmente, nossos achados apoiam o uso deste guia validado em vários contextos epidemiológicos e culturais no Brasil.

Fan Hui Wen

Jacqueline Sachett

Wuelton Monteiro

toxins MDPI

SUMÁRIO

AO1	Primeiros socorros	8
AO2	Diagnóstico e classificação clínica do envenenamento	12
AO3	Realização do exame do tempo de coagulação	17
AO4	Preparo do antiveneno para administração	19
AO5	Tratamento antiveneno específico	23
AO6	Cuidados com o local da picada	29
AO7	Seguimento do paciente	33
AO8	Encaminhamento do paciente para unidade hospitalar	36
AO9	Avaliação das complicações do acidente ofídico	38
AO10	Alta hospitalar e acompanhamento ambulatorial	41
AO11	Alta da unidade básica e acompanhamento	42
AO12	Preenchimento da ficha de notificação	43
AO13	Recebimento e armazenamento de antiveneno	46

AO1 – Primeiros socorros

Quando suspeitar ou critérios de inclusão:

Em qualquer suspeita de contato com serpente.

Conduta:

1. Avaliar a responsividade do paciente (chamar o paciente);
- Se não responsivo, pedir ajuda (SAMU) e checar o pulso;
- Na falta de pulso, iniciar manobras conforme os protocolos de suporte básico de vida da *American Heart Association*;
- Durante os ciclos de manobras, checar o pulso a cada 2 minutos;
- Se responsivo, prosseguir a avaliação.

2. Avaliar o ambiente onde ocorreu o acidente e onde o paciente se encontra;
- Verificar se a serpente ainda está próxima ao paciente e afastá-la do local;
- Não estimular a captura da serpente causadora do acidente, pois há risco de nova picada. Contudo, caso o paciente já tenha executado a serpente, ele poderá levá-la (de preferência em frasco fechado com álcool) ou tirar uma foto nítida para reconhecimento na unidade de saúde, pois a sua identificação correta permite um diagnóstico preciso do acidente.
- Avaliar se existe risco de outras lesões no ambiente com outros animais e/ou objetos.

3. Avaliar o local da picada e manifestações do acidente
- Verificar a presença de lesão puntiforme ou escoriação causada pela picada da serpente na pele do paciente;
- Lavar a região da picada com água limpa e sabão, quando possível;
- Acompanhar os sinais e sintomas locais e sistêmicos durante o transporte, como: edema, intensidade da dor, coloração da pele, presença de sangramentos, dentre outros;
- Se possível, evitar que o paciente se movimente; manter o membro acometido em posição confortável, de preferência, em posição elevada até a chegada no serviço de saúde.

4. Manter o paciente hidratado
- Se o paciente estiver responsivo, deixar que a ingestão de água seja à vontade, com cuidado para evitar que vomite.

5. Transportar, o mais rápido possível, o paciente para uma unidade de saúde.

Observação:
- Nunca utilizar torniquete ou realizar qualquer outro procedimento no local da picada como sucção, incisão, aplicação de substâncias e produtos caseiros, pois, além de não haver evidência de sua eficácia, podem causar complicações no local da lesão;

- Nunca indicar que o paciente ingira misturas caseiras: chás, garrafadas ou outras preparações como "Específico Pessoa", pois também não existe nenhuma evidência para neutralização do veneno ou outro benefício para o paciente.

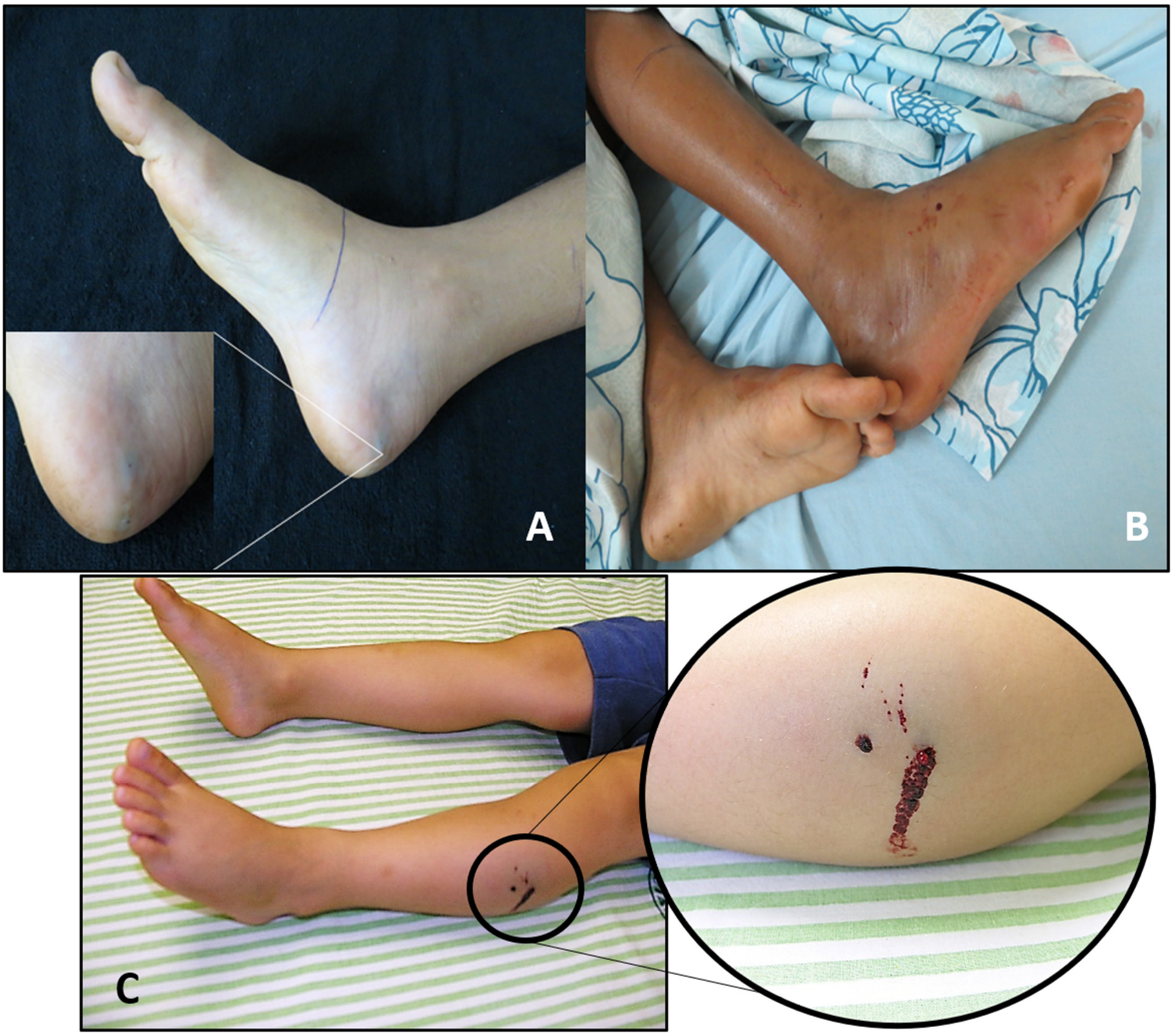

Figura 1 – (A) Lesão puntiforme no calcanhar direito; (B) sinais locais com o dorso do pé edemaciado e avermelhado, com sangramento no local da picada; (C) Lesão puntiforme na panturrilha esquerda. *Imagens do acervo do CEPCLAM/FMT-HVD.*

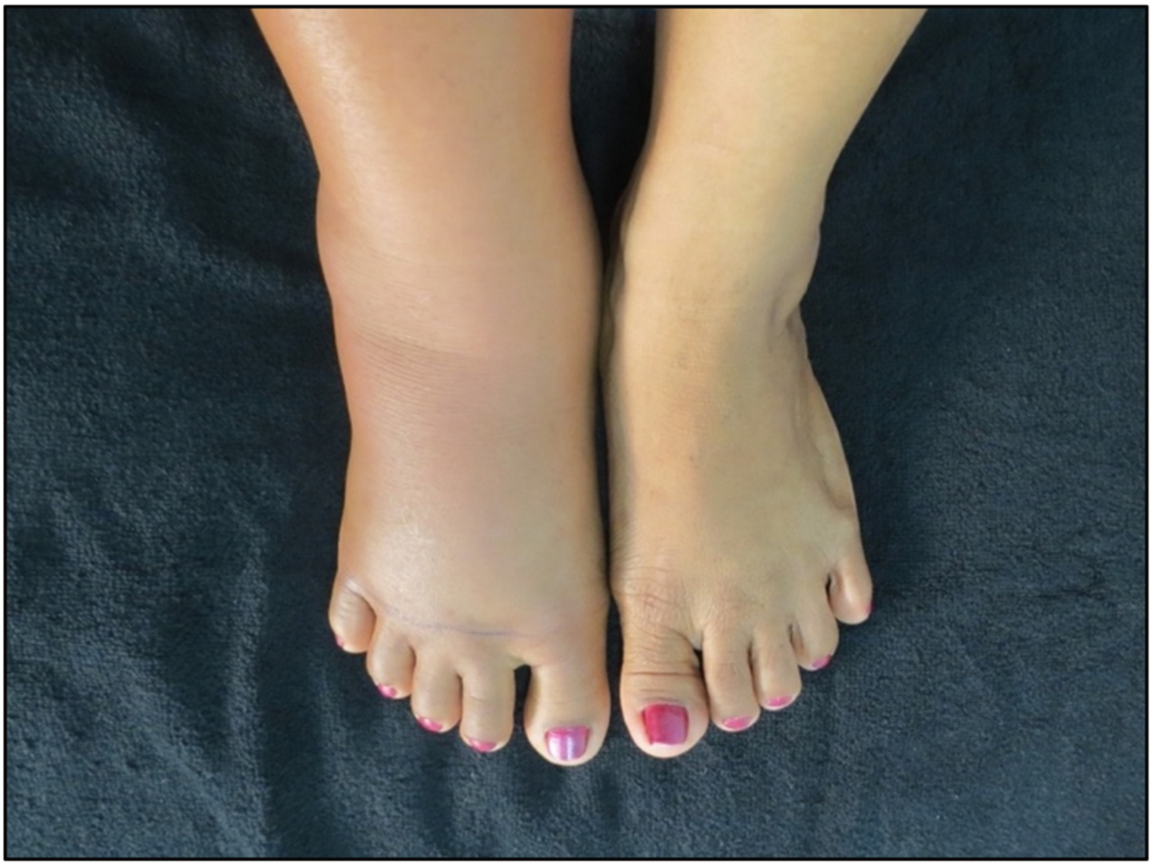

Figura 2 – Edema evidente no pé e perna, com pele levemente avermelhada, sinais clássicos do envenenamento botrópico. *Imagens do acervo do CEPCLAM/FMT-HVD*

PRIMEIROS SOCORROS

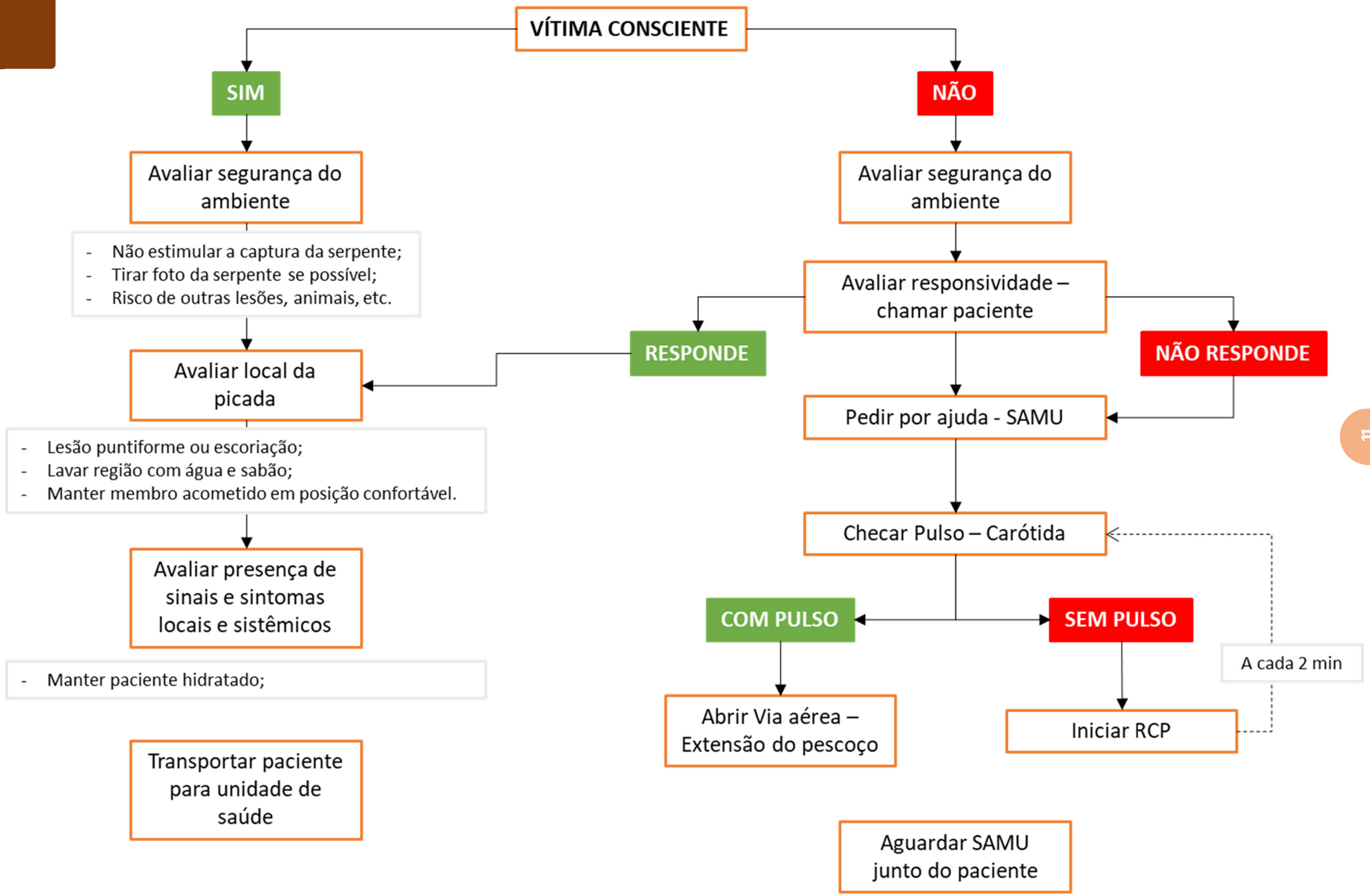

AO2 - Diagnóstico e classificação clínica do envenenamento

O paciente deve ser avaliado quanto há presença de sinais e sintomas clínicos característicos de envenenamento, considerando a epidemiologia do caso e a descrição ou a identificação do agente causador.

Conduta:

- Avaliar sinais e sintomas de envenenamento consoante as características clínicas e epidemiológicas, como descrito a seguir:

---

i. ***Bothrops* (jararaca ou surucucu):** corresponde ao acidente ofídico de maior dimensão epidemiológica no país, sendo responsável pela quase totalidade dos casos no Amazonas.

O envenenamento botrópico é caracterizado pela **dor** imediata no local da picada, e posterior aparecimento de edema de intensidade variável, em geral, de instalação precoce. O edema pode se restringir ao local da picada, mas é comum progredir ao longo do membro acometido. **Sangramentos** pelos pontos da picada são frequentes. Equimoses podem aparecer ao redor do local da picada ou em outras partes do membro.

Podem aparecer sangramentos em ferimentos cutâneos preexistentes, ou de aparecimento espontâneo, como **gengivorragia, hematúria, sangramento conjuntival, epistaxes, hemoptise e hematêmese**.

O sangue do paciente pode estar incoagulável, podendo ser detectado pelo Tempo de Coagulação (vide AO3). O TC não é empregado na avaliação da gravidade do envenenamento, mas é importante que o teste seja realizado na admissão para o seguimento do paciente e acompanhamento pós-administração do antiveneno.

Podem ocorrer **náuseas, vômitos, sudorese e cefaleia.**

Manifestações menos comuns, observadas em pacientes que demoram para receber tratamento, são: bolhas no membro acometido com conteúdo líquido que pode variar de coloração, podendo chegar a sanguinolento; síndrome compartimental, quando ocorre compressão do feixe vásculo-nervoso devido à intensidade do edema; infecção secundária por contaminação por bactérias presentes na boca da serpente ou bactérias presentes na pele do paciente e até por aplicação de substâncias caseiras e necrose de pele e subcutâneo. Pode haver também **oligúria.**

a) **Leve:**	Modo mais comum do envenenamento, caracterizada por **dor** e **edema local** pouco intenso ou ausente, manifestações **hemorrágicas discretas ou ausentes. TC normal ou alterado.**
b) **Moderado:**	Caracterizado por **dor** e **edema evidente que ultrapassa o segmento anatômico picado. Envenenamentos moderados podem ser acompanhados (ou não) de alterações hemorrágicas locais, ou sistêmicas. TC normal ou**

	**alterado.**
c) **Grave:**	Caracterizado por **edema local intenso e extenso**, podendo atingir todo o membro picado, geralmente acompanhado de **dor intensa e, eventualmente, com presença de bolhas**. Em decorrência do edema, podem aparecer sinais de **síndrome compartimental**. Manifestações sistêmicas como **hipotensão arterial, choque, oligoanúria ou hemorragias intensas** definem o caso como grave, independentemente do quadro local. **TC normal ou alterado.**

ii. ***Lachesis* (surucucu-pico-de-jaca):** por se tratar de serpentes encontradas em áreas florestais fechadas, onde sua densidade populacional é baixa, os acidentes por *Lachesis* são muitos raros.

O quadro clínico é muito semelhante ao descrito para os envenenamentos botrópicos, exceto pela possibilidade de ocorrência de **síndrome vagal (hipotensão arterial, tonturas, escurecimento da visão, bradicardia, cólicas abdominais e diarreia).**

a) **Moderado:**	Quadro local presente, pode haver sangramento sem manifestações vagais. **TC normal ou alterado.**
b) **Grave:**	Quadro local intenso, hemorragia intensa e/ou com manifestações vagais. **TC normal ou alterado.**

iii. ***Crotalus* (cascavel):** não ocorre com abrangência geográfica no estado do Amazonas, exceto, predominantemente, no município de Humaitá. As cascaveis só habitam em regiões de campos abertos, pedregosos e em áreas de cerrado.

As manifestações locais são pouco importantes, diferindo dos acidentes botrópico e laquético. Nesta, **não há dor**, ou pode ser de pequena intensidade. Há **parestesia local ou regional**, que pode persistir por tempo variável, podendo ser acompanhada de **edema discreto** ou eritema no ponto da picada.

Manifestações neurológicas surgem nas primeiras horas após a picada, evidenciada por paralisia de grupos musculares, com progressão crânio-caudal, iniciando-se, em geral, com **ptose palpebral** uni ou bilateral**, alteração do diâmetro pupilar, incapacidade de movimentação do globo ocular** (oftalmoplegia), podendo existir dificuldade de acomodação (**visão turva**) e/ou **visão dupla** (diplopia). **A flacidez da musculatura da face pode provocar sialorreia e dificuldade de deglutição, podendo apresentar ainda: alteração de olfato e paladar.** Possíveis **dores musculares generalizadas** (mialgias) podem aparecer por lesão de músculos esqueléticos. Além disso, conseguinte liberação do pigmento de mioglobina no sangue e na urina podem aparecer juntamente com urina de cor avermelhada ou de tonalidade mais escura, podendo chegar até o marrom (mioglobinúria).

A progressão da fraqueza muscular pode comprometer os músculos da caixa torácica e levar o paciente a insuficiência respiratória aguda.

Alteração na coagulação sanguínea, com ou sem sangramentos, ocorre em menor frequência.

a) **Leve:**	Caracteriza-se pela presença de sinais e **sintomas neuroparalíticos discretos**, **sem mialgia, alteração da cor da urina ou oligúria. TC normal ou alterado.**
b) **Moderado:**	Caracteriza-se pela presença de **sinais e sintomas neuroparalíticos evidentes**, **mialgia discreta e a urina pode apresentar coloração alterada. TC normal ou alterado.**
c) **Grave:**	Os **sinais e sintomas neurotóxicos são evidentes e intensos**, a **mialgia é intensa**, a **urina é escura, podendo haver oligúria ou anúria. TC normal ou alterado.**

iv. ***Micrurus* (coral verdadeira):** raramente registrados.

Há **discreta dor local**, geralmente acompanhada de **parestesia**. Caracteriza-se pelo quadro **de paralisia muscular progressiva**, ocorrendo **ptose palpebral**, e outros sinais neurológicos, como **turvação visual, oftalmoplegia,** e **paralisia de músculos da face**. Associadas a estas manifestações, podem surgir dificuldades para manutenção da posição ereta, **mialgia localizada ou generalizada** e **dificuldade para deglutir**. A **paralisia flácida da musculatura respiratória** compromete a ventilação, podendo haver evolução para **insuficiência respiratória aguda e apneia.**

Todos ***os casos de acidente por coral*** com manifestações clínicas **devem ser considerados como potencialmente graves pelo risco de insuficiência respiratória.**

- Picadas secas: ocorre quando uma serpente peçonhenta não injeta veneno no momento do ataque. Nestes casos, pode ser observada as **marcas das presas**, porém **sem sinal local ou sistêmico de envenenamento**, e os **PACIENTES NÃO NECESSITAM ANTIVENENO**.
- Caso o paciente chegue ao atendimento sem sinais de envenenamento, deve permanecer sob observação por, no mínimo, 6 horas após o acidente. Recomenda-se realizar o TC assim que o paciente é admitido e ao final do período de observação. Não havendo manifestações clínicas ou alteração do TC, o paciente pode receber alta com o diagnóstico de picada seca.

Observação:

- Os envenenamentos apresentam quadros locais e sistêmicos, sendo que, a classificação clínica final será baseada no pior quadro entre os dois. Exemplo: paciente de acidente botrópico com edema leve (**quadro local leve**) e hematúria (**quadro sistêmico moderado**) deverá ter sua classificação final como **MODERADO**.

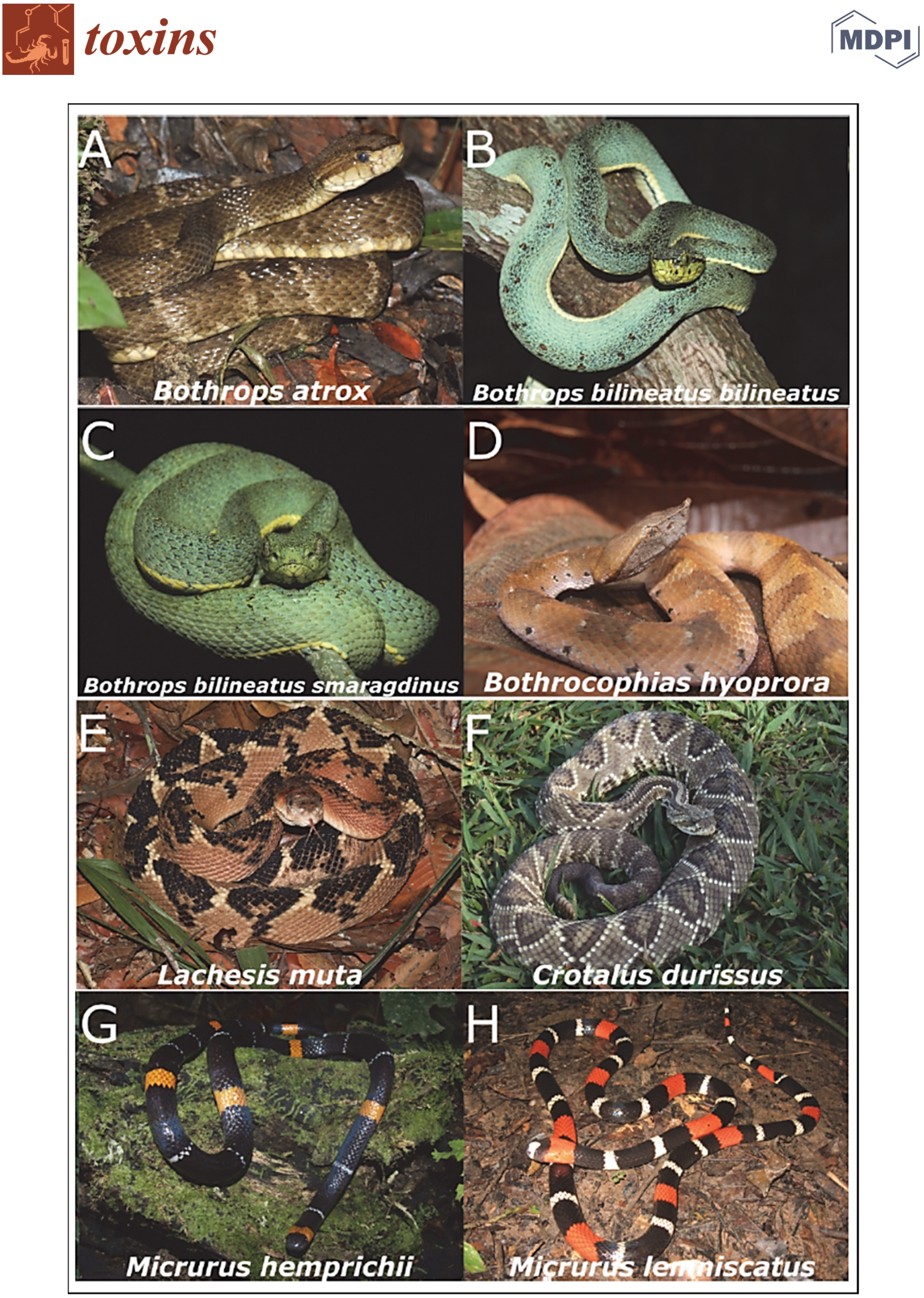

Figura 3 – Espécies de serpentes envolvidas em acidentes ofídicos na Amazônia brasileira. As imagens mostram as oito principais espécies de serpentes responsáveis por picadas na região amazônica brasileira (A – H). *Bothrops atrox* (A) é responsável pela maioria das picadas de cobras registradas na região Amazônia brasileira (80-90%), seguido por *Lachesis muta* (E). *Fonte: Hui Wen F,*

Monteiro WM, Moura da Silva AM, et al. (2015) Snakebites and Scorpion Stings in the Brazilian Amazon: Identifying Research Priorities for a Largely Neglected Problem. PLoS Negl Trop Dis 9(5): e0003701. doi:10.1371/journal.pntd.0003701

DIAGNÓSTICO E CLASSIFICAÇÃO

SINAIS E SINTOMAS

Bothrops
Jararaca / Surucucu

- Dor;
- Edema;
- Hemorragia discreta;
- TC normal ou alterado.

Leve

- Dor;
- Edema evidente; (ultrapassa segmento picado);
- Hemorragia local ou sistêmica;
- TC normal ou alterado.

Moderado

- Dor intensa;
- Edema local intenso ou extenso;
- Bolhas;
- Síndroma compartimental;
- Hipotensão arterial / Choque;
- Oligoanúria;
- Hemorragias intensa;
- TC normal ou alterado.

Grave

Lachesis
Surucucu Pico de Jaca

- Quadro local presente;
- Sangramento sem manifestações vagais;
- TC normal ou alterado.

Moderado

- Quadro local intenso;
- Hemorragias intensas;
- Manifestações vagais.

Grave

Crotalus
Cascavel

- Sinais e sintomas neuroparalíticos leves;
- Sem mialgia;
- Oligúria;
- TC normal ou alterado.

Leve

- Sinais e sintomas neuroparalíticos evidentes;
- Mialgia discreta;
- Urina com coloração alterada;
- TC normal ou alterado.

Moderado

- Sinais e sintomas neurotóxicos intensos e evidentes;
- Mialgia intensa;
- Urina escura (oligúria ou não);
- TC normal ou alterado.

Grave

Micrurus
Coral verdadeira

- Dor;
- Parestesia;
- Paralisia muscular progressiva;
- Ptose palpebral;
- Turvação visual;
- Oftalmoplegia;
- Paralisia dos músculos faciais.

Grave

AO3 – Realização do exame do tempo de coagulação

O paciente deve ser avaliado quanto à presença de alteração no tempo de coagulação diante da presença ou ausência dos sinais e sintomas clínicos característicos de envenenamento, considerando a epidemiologia do caso e a descrição ou a identificação do agente causador. Pois, como exemplo, em casos leves de acidentes botrópicos, apenas o Tempo de Coagulação pode estar alterado.

Conduta:

1. Realizar uma punção venosa com seringa de plástico e coletar 4 ml de sangue do paciente;
2. Adicionar 2 ml de sangue em cada um de dois tubos de vidro 13x100 mm de diâmetro iguais, escorrendo pela parede do tubo;
3. Nesse momento, acionar o cronômetro;
4. Levar imediatamente os tubos de vidro para o banho-maria a 37 ºC;
5. A partir do 5º minuto, e a cada 1 minuto, fazer a leitura sempre em um dos tubos de vidro, inclinando-o suavemente a 90º para observar a formação de coágulo de sangue. Manter o segundo tubo de vidro em repouso no banho-maria, o qual será utilizado como controle;
6. Para este método, os valores são:

Tempo para formação do coágulo	Interpretação do Resultado
até 9 minutos	tempo de coagulação **normal**
de 10 a 30 minutos	tempo de coagulação **prolongado**
acima de 30 minutos	tempo de coagulação **incoagulável**

7. Após a administração do antiveneno, o exame de TC deve ser realizado após 12 horas.

Observação: O tempo de coagulação **NÃO** é um exame para confirmar envenenamento e sim um exame para auxiliar na evolução da anormalidade da coagulação que o veneno da botrópico e laquético podem causar. O paciente com tempo de coagulação normal ou alterado terá a confirmação do envenenamento sempre pelo critério clínico.

- Exames Laboratoriais Complementares

Quando viável, alguns exames laboratoriais devem ser realizados no momento da entrada do paciente na unidade de saúde e durante o seguimento atendimento (acompanhamento do caso), para auxílio da conduta bem como identificação de possíveis agravos provenientes do acidente:
- Hemograma completo;
- Ureia e creatinina;
- Creatina quinase (CK);
- Exame de EAS (urina);
- Proteína C reativa.

REALIZAÇÃO DO EXAME DE TEMPO DE COAGULAÇÃO - TC

PROCEDIMENTOS

↓

Punção venosa

↓

2 tubos de vidro
2 ml de sangue em cada vidro

↓

Acionar o cronômetro

↓

Colocar em banho maria;
E a partir do 5º minuto, avaliar de 1 em 1 minuto

↓

RESULTADO
Tempo para formação de coágulo

Até 9 minutos	10 a 30minutos	acima de 30 minutos
**NORMAL**	**PROLONGADO**	**INCOAGULÁVEL**

AO4 – Preparo do antiveneno para administração

Quando suspeitar ou critérios de inclusão:

O preparo do Antiveneno deve ser realizado no momento da administração, ou seja, após 30 min da pré-soroterapia, conforme descrição no AO5.

Conduta:

1. Verificar se o antiveneno está conservado sob refrigeração entre 2 ºC e 8 ºC ou siga as orientações da bula, onde contém a temperatura ideal de conservação do soro.

2. O antiveneno não deve estar ou ter sido congelado. Veja figura 4 o frasco do antiveneno.

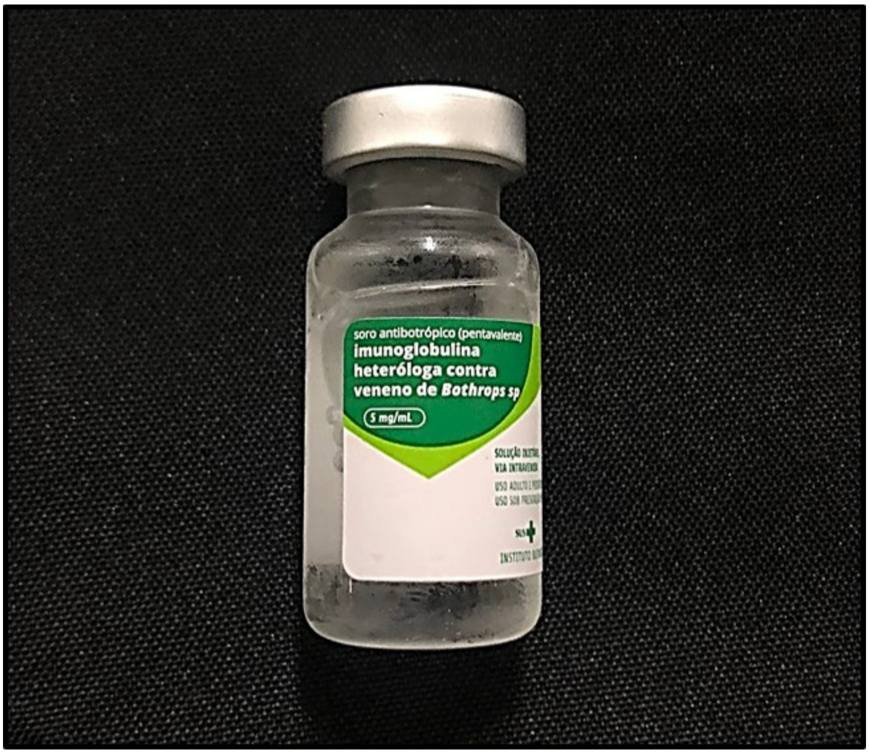

Figura 4 - Soro antiveneno. *Imagens do acervo do CEPCLAM/FMT-HVD.*

3. Lavar as mãos e calçar as luvas de procedimentos.

4. Reúna o material necessário para o preparo do soro antiveneno: seringa, agulha, bola de algodão e antisséptico (álcool a 70º).

5. O número do lote, data de fabricação, nome do fabricante e validade devem ser verificados no recipiente (rótulo e ampola) e devem ser anotados no registro do paciente. Não use medicamentos com prazo de validade vencido.

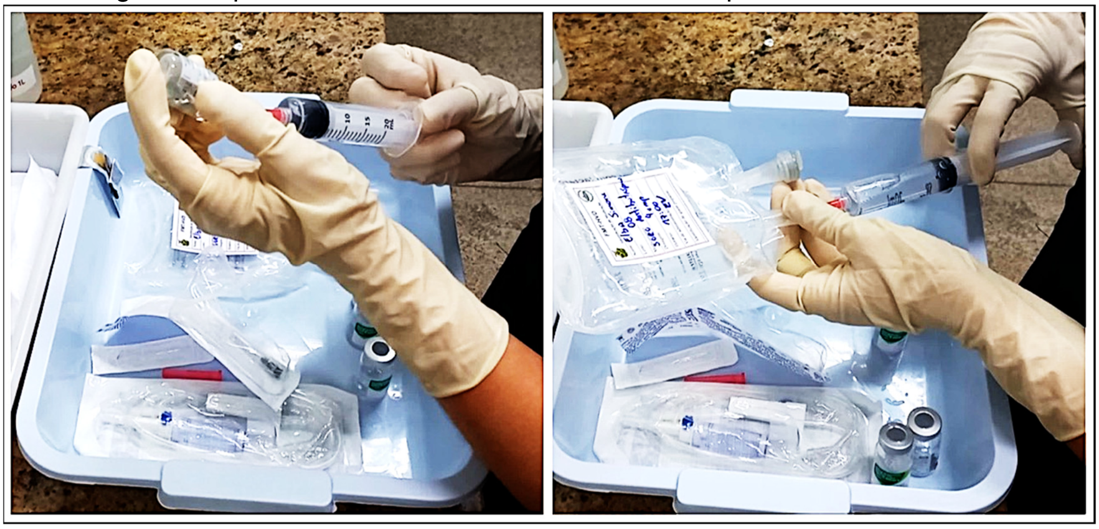

Figura 5 – Procedimento de preenchimento de frasco vazio com soro antiveneno para administração. *Imagens do acervo do CEPCLAM/FMT-HVD.*

6. Abrir um frasco de soro glicosado ou fisiológico e esvaziar para que fique em seu interior apenas 100 ml de solução; abrir todos os frascos do soro antiofídico prescritos e com ajuda de uma seringa, aspirar e colocar no interior de um frasco de soro contendo os 100ml de soro glicosado ou fisiológico (Figura 5), conforme o volume final do antiveneno com a solução. Observe, na **Figura 6,** os materiais que serão utilizados.

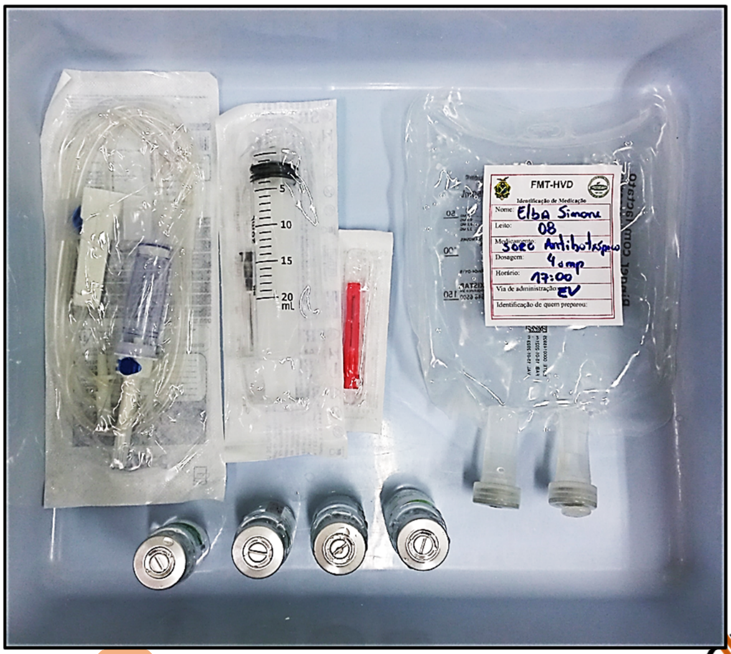

Figura 6 – Materiais utilizados para a administração do soro antiveneno. *Imagens do acervo do CEPCLAM/FMT-HVD.*

7. Após abertura dos frascos, o antiveneno deve ser administrado imediatamente. O antiveneno é uma solução límpida, incolor ou ligeiramente amarelada que não deve apresentar grumos ou partículas. Não deve ser usado se houver turvação (diminuição da transparência) ou presença de material estranho. Veja, na **Figura 7,** o aspecto correto do soro**.**

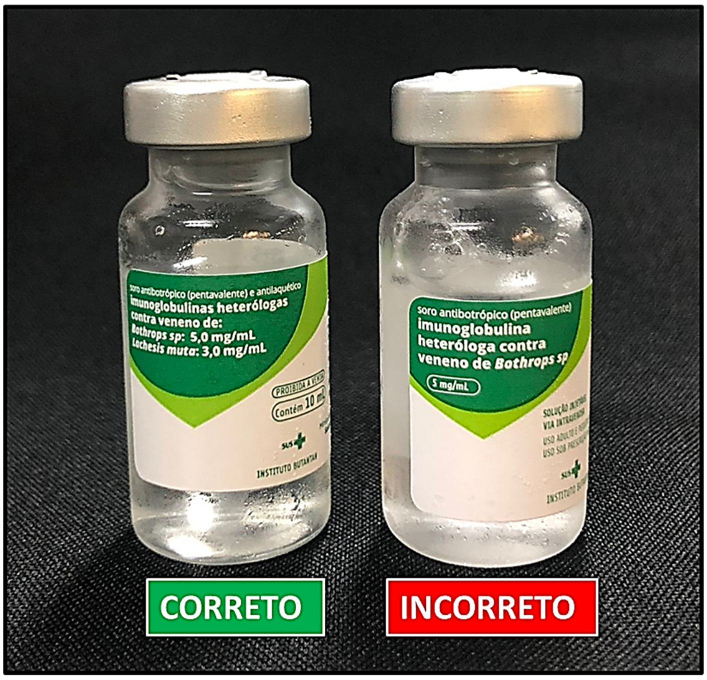

Figura 7 – Aspecto do soro antiveneno.
Imagens do acervo do CEPCLAM/FMT-HVD.

8. Caso o antiveneno esteja no prazo de validade e você observe alguma mudança no aspecto, consulte o farmacêutico para saber se poderá utilizá-lo.

9. O antiveneno será administrado seguindo as doses indicadas pelo médico, <u>**em infusão lenta, entre 20 e 60 minutos na via intravenosa**</u> (IV) e o antiveneno, **diluído ou não em solução fisiológica**.

10. O antiveneno pode ser administrado a qualquer momento, mesmo após refeições ou ingestão de bebidas alcoólicas, mas exige cuidado mais rigoroso desses pacientes pelo risco de complicações relacionadas a vômitos (aspirações).

11. O uso de outros medicamentos concomitantes com o antiveneno não compromete o tratamento.

Observação:

Em caso de medicação vencida ou duvidosa seguir o protocolo do Programa Naci[illegible]
Imunização da Secretaria de Saúde.

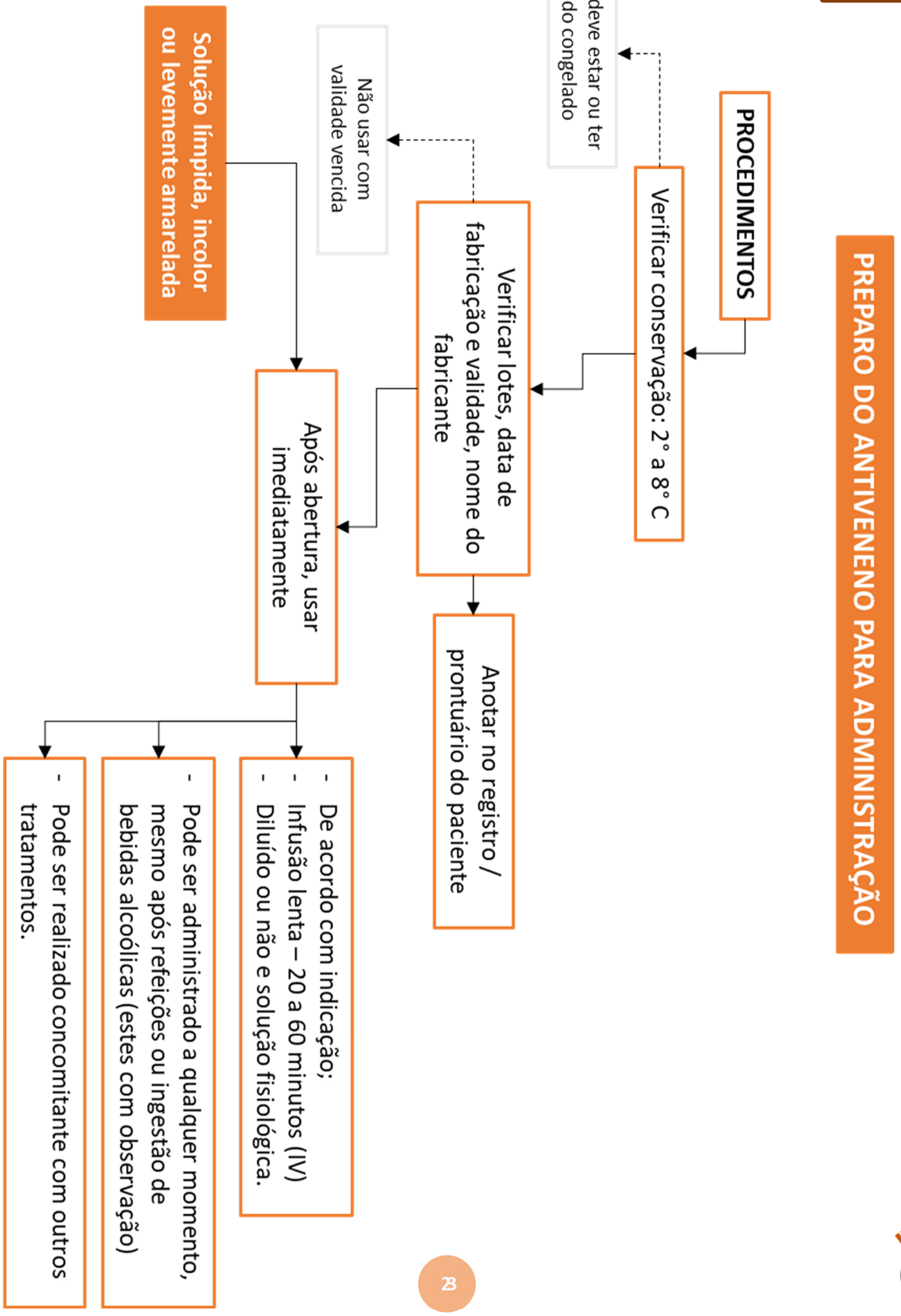

AO5 - Tratamento antiveneno específico

A escolha do tipo e da posologia do antiveneno é feita segundo o diagnóstico e gravidade do quadro clínico, conforme descrito anteriormente no AO2.

Conduta:

1. **Pré-soroterapia**

- Obter a história pregressa do paciente quanto a quadros alérgicos de qualquer natureza. Em caso positivo, a maior atenção deve ser necessária no momento da administração do antiveneno.
- A medicação pré-soroterapia pode ser realizada com algum antagonista dos receptores H1, como a dexclorfeniramina, loratadina e difenidramina.
- No caso da dexclorfeniramina e da loratadina, drogas usadas por via oral, deve-se realizar a pré-soroterapia 30 minutos antes da administração do soro.
- No caso da difenidramina via intravenosa, pode-se realizar a pré-soroterapia de 5 a 10 minutos antes da administração do soro.

Classe	Medicação	Dose
Antagonistas de receptores H1	Dexclorfeniramina	0,05 mg/kg por VO (crianças e adultos)
	Loratadina	• Adulto - 0,05 mg/kg por VO;<br>• Crianças de 2 a 6 anos – 5 mg por VO (dose única);<br>• Crianças acima de 6 anos – 10 mg por VO (dose única);<br>• Crianças abaixo de 2 anos – Fazer uso da dexclorfeniramina
	Difenidramina	• Adulto - 25 a 50 mg em adultos (ampola com 1 mL);<br>• Crianças acima de 2 anos - 5 mg/kg/24horas

- Apesar da ausência atual de evidência científica para a pré-soroterapia, alguns serviços ainda combinam os antagonistas de receptores H1 com corticoides, como a hidrocortisona.

Classe	Medicação	Dose
Corticoide	Hidrocortisona	• Adultos 300 mg;<br>• Crianças (ou abaixo de 50 kg) 5 mg/kg

1

PRÉ-SOROTERAPIA

História – alergias → **SIM** → **Maior atenção**

Medicação pré-soroterapia – 30 minutos antes da soroterapia

- **Difenidramina;**
- **Loratadina**
- **Dexclorfeniramina** (5-10 min. Antes da soroterapia;

+

- **Hidrocortisona**

OBS: Verificar recomendação de dosagens

2. **Soroterapia**

A seguir, estão descritas as doses de antivenenos que devem ser administradas para cada tipo de envenenamento.

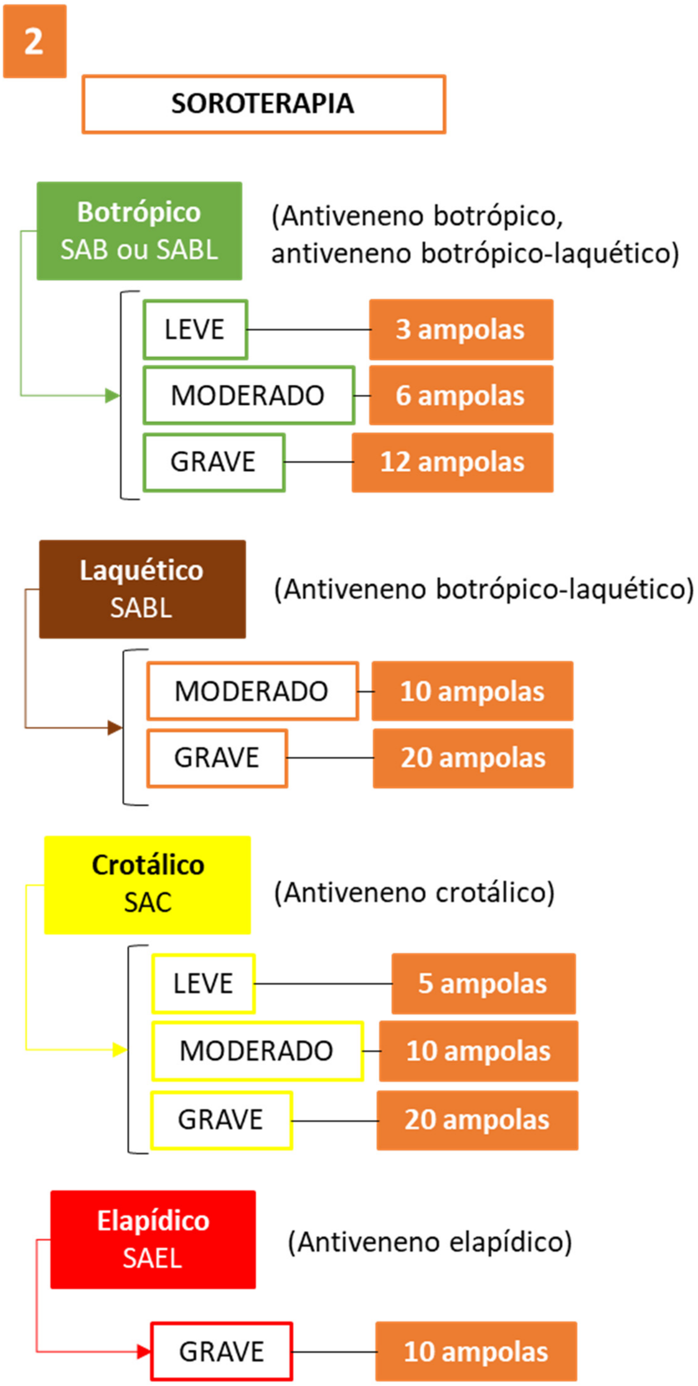

Observações:

- A definição do número de ampolas de soro antiofídico a ser administrado se dá unicamente pela gravidade do envenenamento, e é independente do local da picada, sexo, peso ou idade do paciente.

3. Administração do soro antiofídico

Para prevenção de reações adversas, alguns cuidados específicos são adotados. Os procedimentos básicos para realizar estas atividades são os seguintes:

a. Lavar as mãos com água e sabão.
b. Organizar todo o material: seringa, agulha e outros materiais necessários ao tratamento preventivo da anafilaxia: adrenalina 1:1.000, anti-histamínico, corticoide, oxigênio, soro glicosado e fisiológico, equipo de soro e material para assistência ventilatória.
c. Cateterizar uma veia de grosso calibre no antebraço e instalar o soro fisiológico ou glicosado a 5% no paciente, garantindo adequado acesso venoso e a manutenção do gotejamento, mesmo depois da administração do soro heterólogo.
d. Controlar os sinais vitais antes de iniciar a administração do soro.
e. Antes da administração do soro antiveneno, fazer a verificação: soro antiveneno certo; dose certa; via certa; horário certo; paciente certo.
g. Iniciar o gotejamento lentamente (15 a 20 gotas por minuto) do soro antiofídico prescrito em "y" com o soro fisiológico ou glicosado a 5% previamente instalado no antebraço do paciente.
h. A supervisão do enfermeiro e da equipe de saúde, deve ser permanente durante a aplicação do soro antiofídico com aferição de sinais vitais a cada 15 minutos, pelo menos durante a primeira hora.
i. O paciente deve ter acesso calibroso. No momento da administração, necessita de repouso no leito com constante monitoramento de sinais para reações adversas (prurido, rouquidão, rash cutâneo, relato de sufocamento, aumento da temperatura corporal), devendo ser avaliado a cada 15 minutos, pelo menos durante a primeira hora.
j. Observar continuamente o paciente, por aproximadamente duas horas após a administração do soro. Deve-se manter o acesso venoso com soro fisiológico instalado, controlar o gotejamento, monitorar sinais vitais e observar a coloração e temperatura das extremidades (lábios, dedos). Observar possíveis sinais de inquietação, presença de prurido, urticária, congestão conjuntival, rouquidão, estridor laríngeo, tosse seca, ruídos respiratórios, palidez, sudorese, dificuldade respiratória, queda de pressão arterial, pulso fraco, dentre outros.
k. Em caso de alguma reação adversa, parar imediatamente o gotejamento do soro antiofídico. Não desprezar o volume remanescente, a não ser que haja suspeita de contaminação. Continuar o gotejamento do soro fisiológico ou glicosado a 5% e iniciar o tratamento com as drogas indicadas:

1) Adrenalina: dose inicial de 0,5 mg IM para adultos, e 0,01 mg/kg de peso para crianças, podendo ser repetida a cada 5-10 minutos se a reação persistir ou os sintomas piorarem. É a medicação de primeira escolha para tratamento da reação alérgica.

2) Broncodilatador: no caso de haver sinais de sibilos, administrar agonista β2 de curta ação inalatório, como salbutamol ou terbutalina, idealmente por nebulização.

3) Anti-histamínico (bloqueador anti-H1): maleato de dextroclorfeniramina (adultos 10 mg; crianças 0,2 mg/kg por injeção IV lenta).

4) Corticosteroides Hidrocortisona IV (adultos 100 mg, crianças 2 mg/kg).

l. Assim que o paciente melhorar, reiniciar o gotejamento do soro antiofídico.

m. O paciente deve ser mantido sob observação do profissional de saúde após a aplicação do soro por, pelo menos, 24 horas.

n. Registre a administração do soro antiveneno no prontuário do paciente, registrando: a quantidade e a categoria de antiveneno, hora da administração, a aparência do local, a duração da administração e a tolerância do paciente ao procedimento. Anote qualquer reação adversa.

Administração de tratamentos concomitantes

Analgesia

- Avaliar a intensidade da dor com escala numérica.
- O analgésico deve ser prescrito imediatamente à admissão do paciente. Nas primeiras 24 horas, normalmente, é sistemático e posteriormente deve ser avaliado para administração no caso de relato de dor.
- Dipirona oral ou parenteral, conforme a escala de dor e repouso com membro elevado em posição confortável são medidas que aliviam o quadro doloroso, até que o edema regrida, exceção à síndrome compartimental (nas primeiras horas) ou infecção secundária (mais tardiamente, geralmente após 48 horas após o acidente). Na ausência de dipirona, outros analgésicos, como paracetamol ou ibuprofeno, podem ser utilizados.
- No caso de dor refratária aos tratamentos analgésicos sugeridos acima, indica-se a utilização de tramadol.

Observações:

- Anti-inflamatórios não esteroides, semelhante à aspirina e diclofenaco, não são indicados, pelo risco de lesão renal.
- Lembre-se que quanto mais rapidamente o paciente receber o soro antiofídico, menor as hipóteses de complicações e sequelas pelo envenenamento.
- O tratamento concomitante poderá ser adicionado conforme a necessidade do paciente e a avaliação do profissional de saúde assistente.
- Não existe evidência de que o uso de antibiótico como terapia precocemente possa prevenir o surgimento de infecção secundária.
- No caso da administração do antiveneno em serviço de atenção primária que não possua estrutura para acompanhamento do paciente, encaminhar o paciente para a unidade hospitalar mais próximo após 1 hora do término da administração do antiveneno.

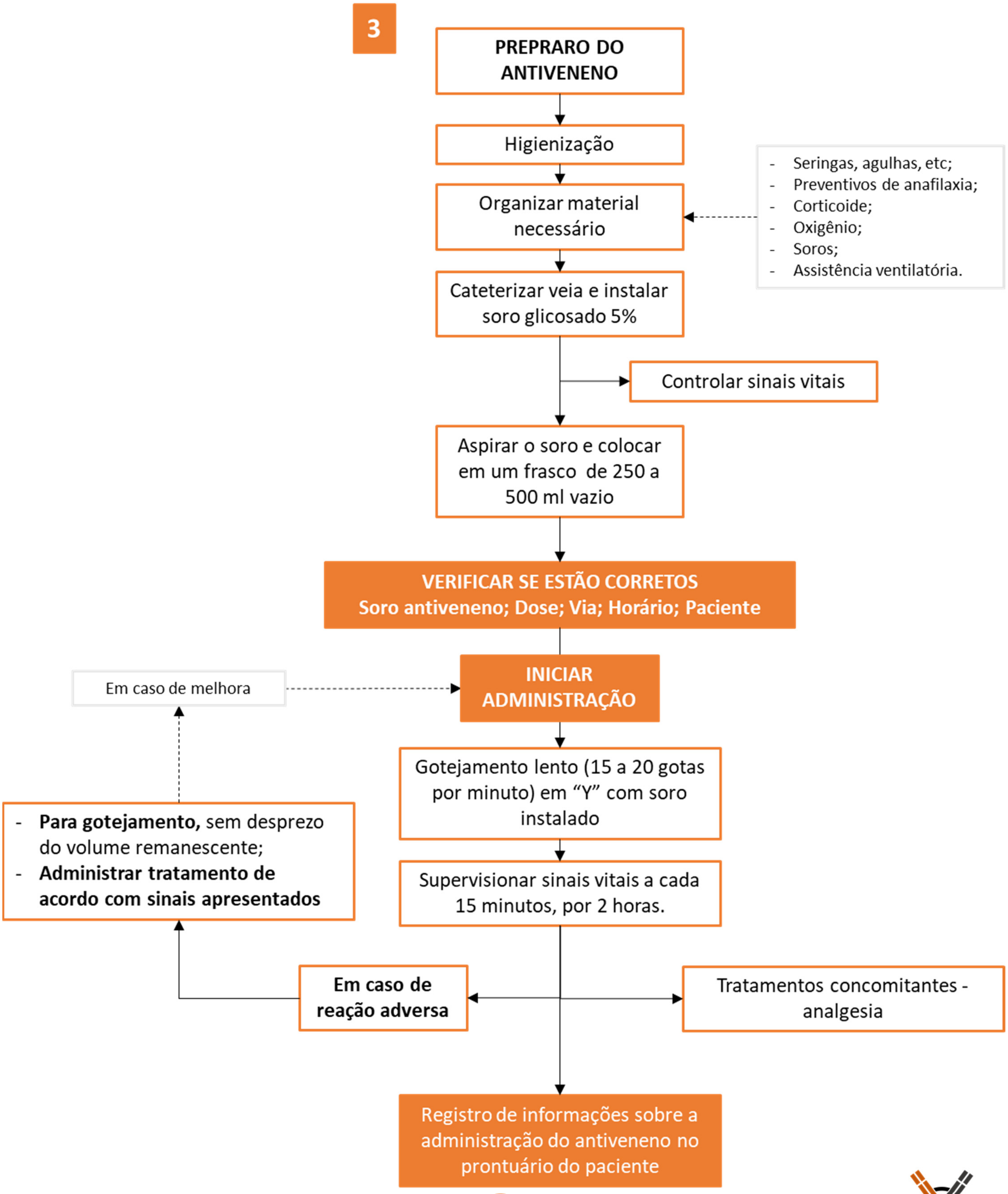
3
PREPRARO DO ANTIVENENO
Higienização
Organizar material necessário
- Seringas, agulhas, etc;
- Preventivos de anafilaxia;
- Corticoide;
- Oxigênio;
- Soros;
- Assistência ventilatória.
Cateterizar veia e instalar soro glicosado 5%
Controlar sinais vitais
Aspirar o soro e colocar em um frasco de 250 a 500 ml vazio
VERIFICAR SE ESTÃO CORRETOS
Soro antiveneno; Dose; Via; Horário; Paciente
Em caso de melhora
INICIAR ADMINISTRAÇÃO
Gotejamento lento (15 a 20 gotas por minuto) em "Y" com soro instalado
- Para gotejamento, sem desprezo do volume remanescente;
- Administrar tratamento de acordo com sinais apresentados
Supervisionar sinais vitais a cada 15 minutos, por 2 horas.
Em caso de reação adversa
Tratamentos concomitantes - analgesia
Registro de informações sobre a administração do antiveneno no prontuário do paciente

AO6 - Cuidados com o local da picada

O local da picada deve ser avaliado diariamente para escolher a melhor conduta.

Conduta:

1- Realizar a limpeza do local da lesão com soro fisiológico e, em casos de sangramento, realizar cobertura com gaze estéril e atadura frouxa. Caso a lesão não tenha sangramento ou exsudato, não necessita uso de cobertura;

2- Avaliar a lesão puntiforme e áreas adjacentes para a intensidade dos sinais flogísticos (calor, rubor e dor);

3- Observar a evolução do edema nas primeiras horas para avaliar a necessidade de reclassificação da gravidade;

4- Avaliar a extensão do edema contando por segmento no membro afetado (segmentos dos membros superiores: mão, ½ distal do antebraço, ½ proximal do antebraço, ½ distal do braço, ½ proximal do braço-total de 5 segmentos; segmentos dos membros inferiores: pé, ½ distal da perna, ½ proximal da perna, ½ distal da coxa, ½ proximal da coxa). Em casos de picada em outros locais, esta classificação não se aplica;

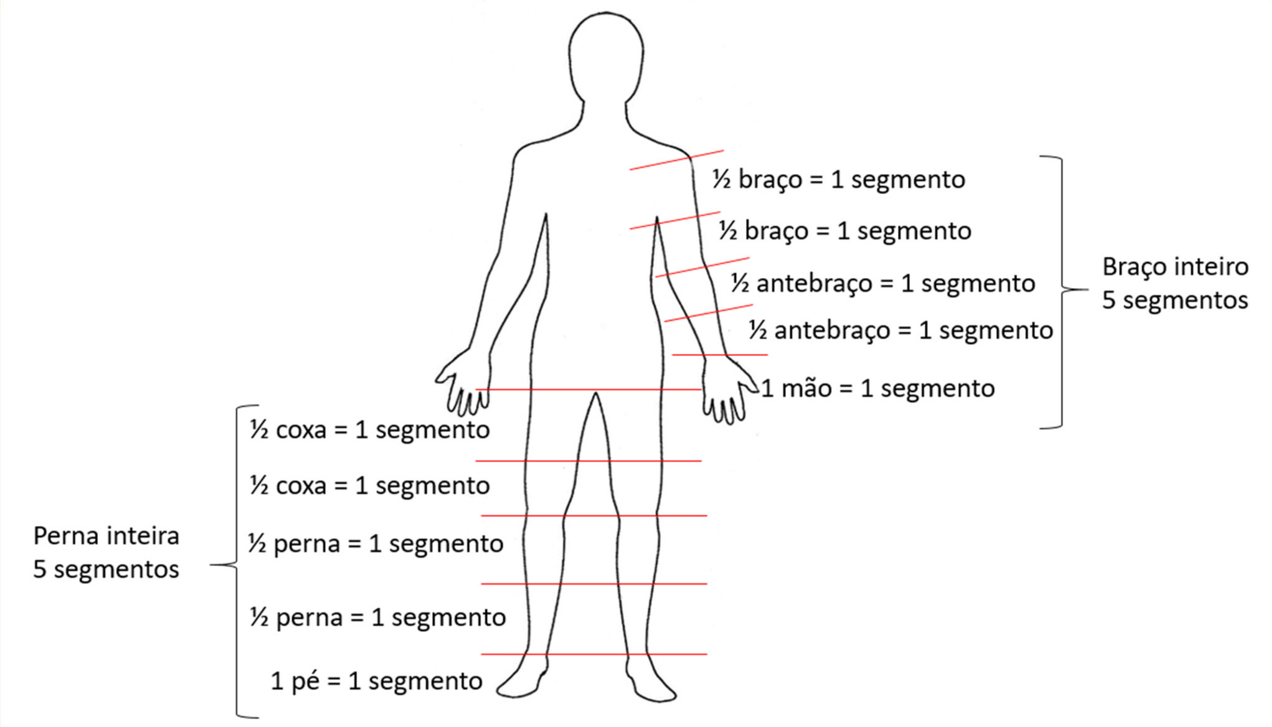

5- Nos casos de acidentes ocorridos em locais diferentes dos membros inferiores e superiores, por exemplo, no tronco ou na cabeça, deve-se fazer a medida da circunferência no local e comparar a evolução com as medidas realizadas nos dias anteriores para observar melhora ou piora do edema pelas diferenças dos centímetros;

6- Verificar diariamente o aparecimento de **sinais de gravidade local** como **aparecimento de bolhas** (Figuras 8A e 8B). Nestes casos, o conteúdo das bolhas do envenenamento pode ainda conter veneno que continua atuando de forma proteolítica (provocando lesão tecidual). Devem ser aspiradas para remover o conteúdo. Após o esvaziamento da bolha, cobrir o local com gaze e fazer curativos diários até a cicatrização completa da lesão;

7- Verificar diariamente o aparecimento de **sinais de hemorragia local** como em casos de equimose no membro afetado ou em regiões mais distantes da picada. Atentar para a formação de bolhas hemorrágicas acentuadas sob a pele que podem formar coágulos sanguíneos e até infectar. Nestes casos, estas bolhas hemorrágicas localizadas devem ser aspiradas ou debridadas. Cobrir o local com gaze e fazer curativos diários até a cicatrização completa da lesão (Figuras 8E e 8F);

8- Verificar diariamente o aparecimento de **sinais de síndrome compartimental** (Figura 3H) como edema endurecido em todo o membro, apresentando dor intensa, persistente e desproporcional. Não sendo responsiva ao uso de analgésicos, ocasionando diminuição ou ausência de pulsos distais, coloração cianótica ou azulada da pele. Nestes casos, tem-se a necessidade de encaminhar o paciente rapidamente para realizar fasciotomia, pois a compressão da circulação sanguínea no membro pode levar a morte do tecido e, consequente amputação;

9- Verificar diariamente o aparecimento de **sinais de infecção** como dor persistente localizada, rubor localizado, flutuação de tecido (tecido amolecido) e secreção purulenta (Figuras 8C e 8D). O aparecimento de febre pode ser um indicativo de infecção secundária; a realização da ultrassonografia pode ser útil, quando possível, para diferenciar o envenenamento da infecção secundária;

10- Verificar diariamente o aparecimento de **sinais de necrose** como coloração escurecida no local da picada ou região próxima, diminuição da perfusão sanguínea no local (isquemia), odor fétido leve a intenso, dor intensa persistente localizada (Figura 8G)

Observação

- Os sinais de complicações no local da lesão devem ser identificados precocemente na tentativa de evitar, ou reduzir o risco de perda funcional do

membro afetado ou repercussões sistêmicas graves que podem levar o paciente a

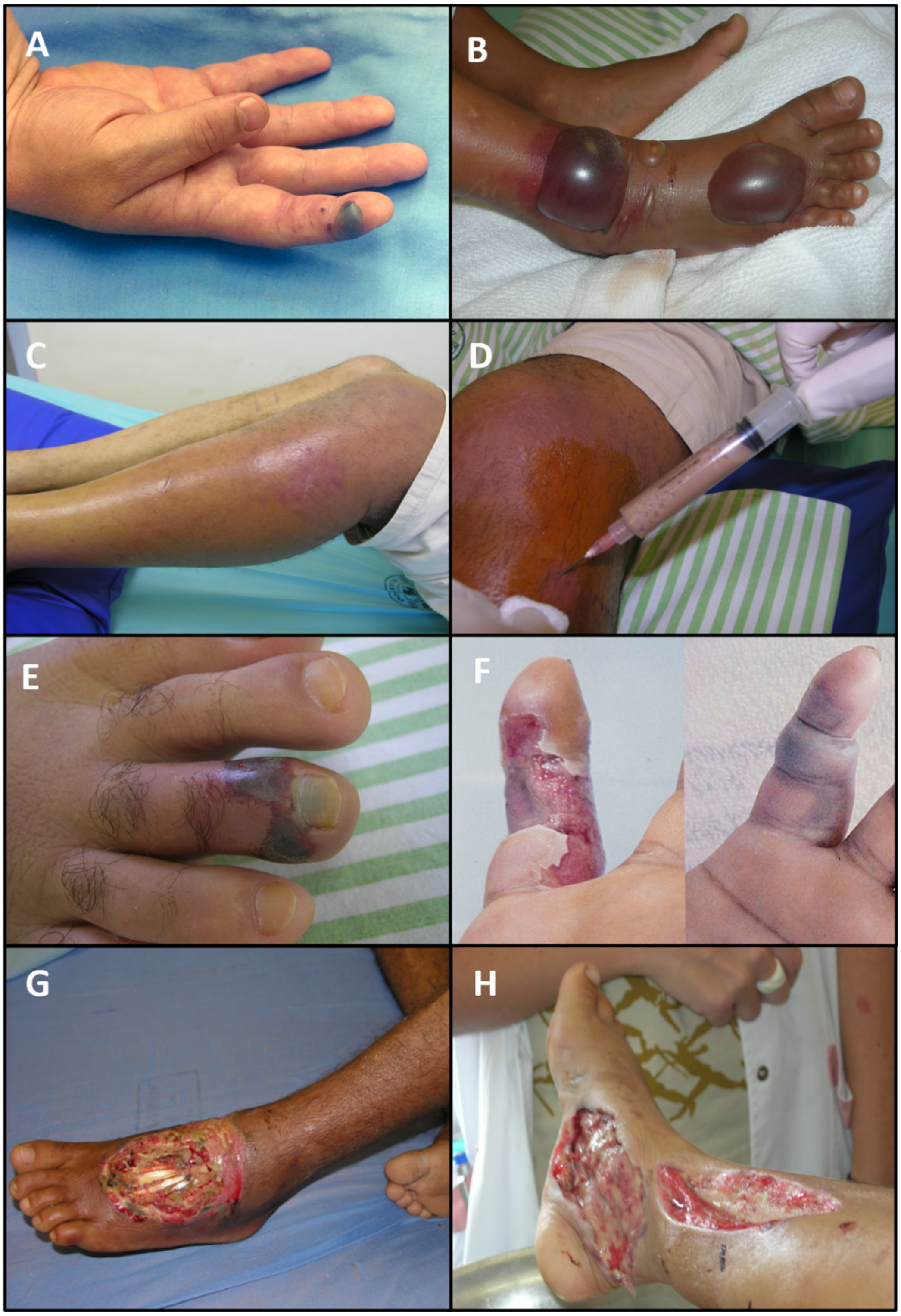

óbito.

Figura 8 – (A) Bolha sanguinolenta no dedo indicador; (B) Bolha sero sanguinolenta no dorso do pé e tornozelo direitos; (C)(D) Infecção secundária com formação de celulite e abscesso. (E)Bolha sanguinolenta em segundo pododáctilo direito; (F) Debridamento em dedo mínimo direito com área de granulação e início de formação de necrose; (G)(H) Formação de úlcera com fibrina e exposição de musculatura após infecção secundária. *Imagens do acervo do CEPCLAM/FMT-HVD*

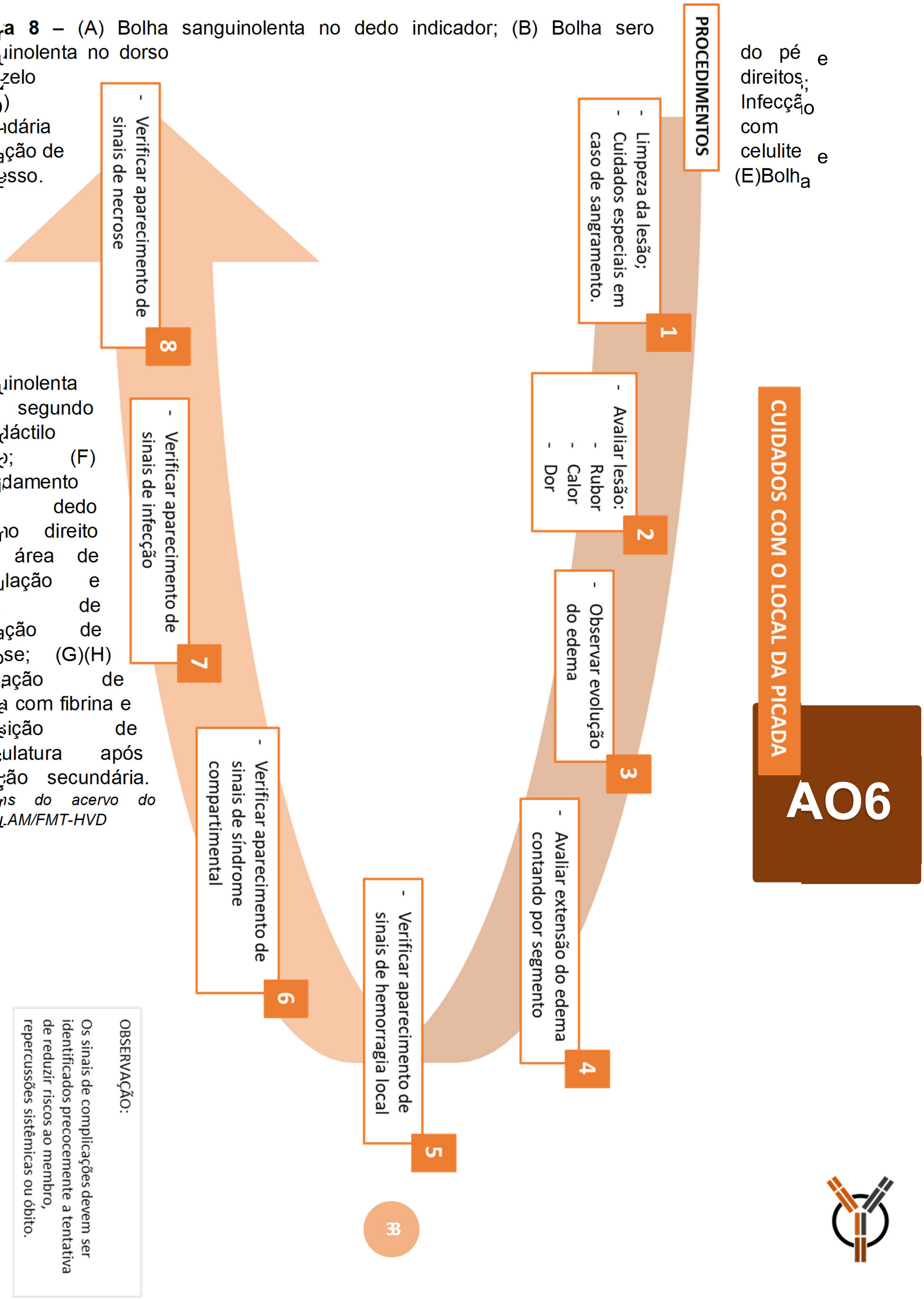

AO7 - Seguimento do paciente

O paciente deve ser avaliado diariamente para identificação de melhora do quadro ou discussão de novas condutas.

Conduta:

1. Avaliação inicial (primeiras 12 horas)
   - Após a administração do soro, a avaliação deve ser direcionada para a melhora da hemorragia e da coagulopatia e, eventualmente, o surgimento de reação adversa precoce que é usualmente observada até as primeiras 2 horas.
   - Realizar o exame de tempo de coagulação (TC) para acompanhar a evolução de coagulopatia a cada 12 horas após a soroterapia até a normalização, que deve ocorrer em até 24 horas após a administração do antiveneno;
   - Avaliar os sinais clínicos locais conforme o protocolo AO1;
   - Avaliar continuamente os sinais sistêmicos de gravidade para sangramento, como hematêmese, enterorragia, otorragia, hematúria macroscópica e, com uso da escala de Glasgow, possível hemorragia cerebral. Espera-se que após a administração do antiveneno, as manifestações hemorrágicas cessem em algumas horas.

2. Reclassificação do acidente ou adição de soroterapia
   - Critérios para reclassificação:
     - Paciente com piora clínica da sintomatologia local (progressão rápida do edema) ou sistêmica (persistência ou surgimento da hemorragia, hipotensão, choque) nas primeiras 12 horas após a administração do antiveneno;
     - Paciente com TC incoagulável em 12 horas. O paciente que apresenta em 12 horas o TC prolongado, significa que está em processo de melhora clínica e deve ser acompanhado com novo exame de TC em 24 para verificar normalização do mesmo, ou, em caso negativo, ser reclassificado.

   Nesses casos, o paciente deverá ser reclassificado quanto a gravidade do acidente ofídico e receber nova dosagem de soro antiofídico nas seguintes situações:

   - O paciente inicialmente diagnosticado como **LEVE** poderá ser reclassificado para **MODERADO** e receber mais 3 (três) ampolas de soro antiofídico (total de 6 ampolas). Espera-se que o paciente tenha melhora do quadro de coagulopatia nas primeiras 24 horas após a primeira administração do soro antiofídico.
   - O paciente inicialmente diagnosticado como **LEVE** poderá ser reclassificado para **GRAVE** e receber mais 9 (nove) ampolas de soro antiofídico (total de 12

ampolas) e ser encaminhado para a unidade de referência o mais rapidamente possível.

- Da mesma forma, casos inicialmente **MODERADOS** poderão ser reclassificados como **GRAVES** e receber mais 6 (seis) ampolas de soro antiofídico (total de 12 ampolas) e ser encaminhado para a unidade de referência o mais rapidamente possível;

- No caso do paciente inicialmente classificado como **GRAVE**, este deverá receber a dose preconizada conforme a gravidade e ser imediatamente encaminhado para a unidade de referência, acompanhamento por profissional de saúde.

Observação:

- A cada administração adicional de soro antiofídico, deve ser avaliada a necessidade de o paciente receber 30 minutos antes a pré-soroterapia conforme o protocolo AO5. Em casos onde a soroterapia inicial foi realizada poucas horas antes ou quando o risco decorrente da gravidade suplantar o risco de reação, deve ser considerada a possibilidade de administrar o soro mesmo sem nova pré-medicação. Por outro lado, se o paciente tiver apresentado reação alérgica moderada ou grave na soroterapia inicial, a administração adicional deve ser considerada com cautela e a pré-medicação instituída.

SEGMENTO DO PACIENTE

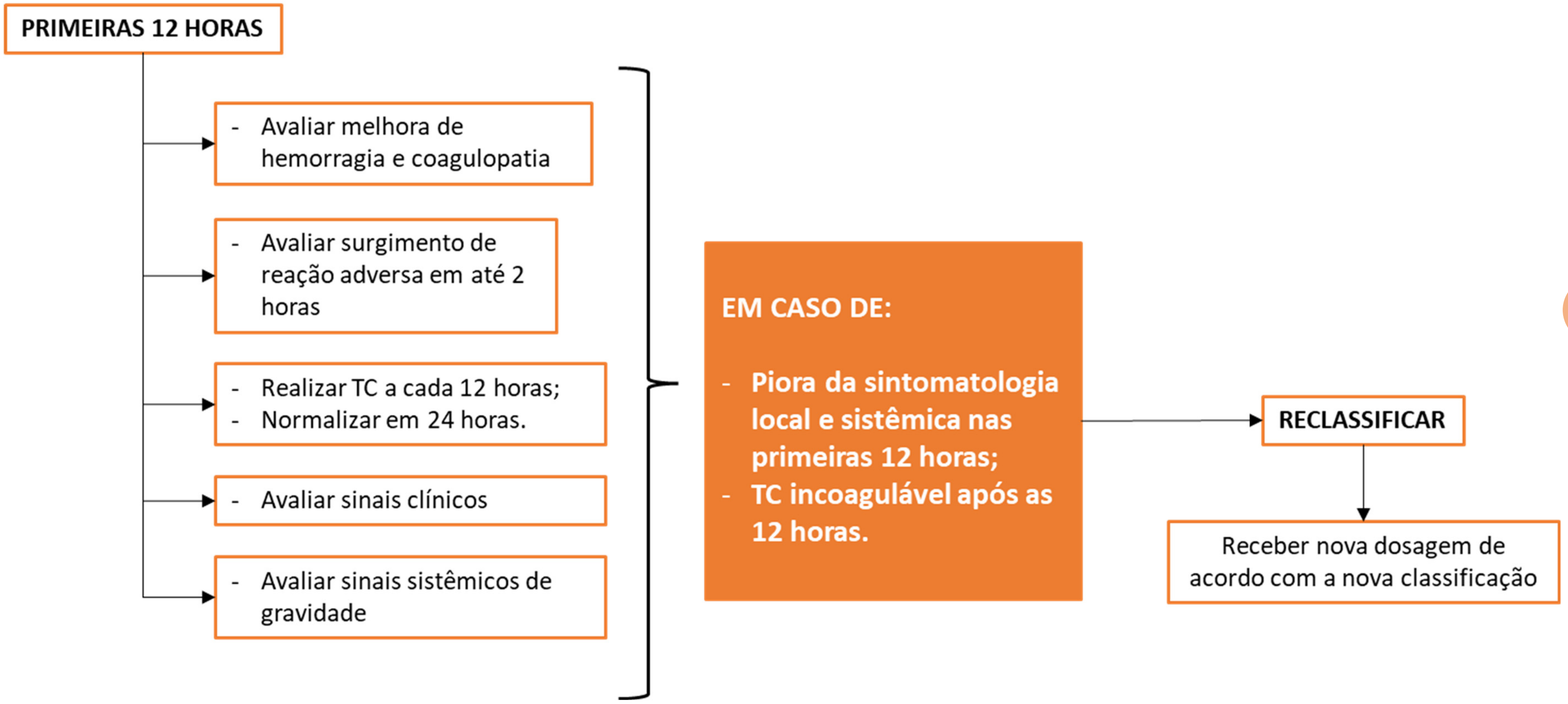

AO8 – Encaminhamento do paciente para a unidade hospitalar

O paciente deve ser avaliado constantemente para identificação de piora do quadro ou discussão de novas condutas. Diante de qualquer sinal de agravamento o paciente deverá ser encaminhado imediatamente a unidade hospitalar mais próxima.

Conduta:

Os Critérios de encaminhamento para encaminhamento as unidades hospitalares estão listados no quadro abaixo:

**Acidente classificado ou reclassificado como grave** (ver AO2 e AO7)	- Quando apresentar edema intenso sugestivo de síndrome compartimental;<br>- Quando houver indícios de hemorragia sistêmica que irá comprometer o volume corporal e regulação dos sistemas;<br>- Evolução para a insuficiência renal aguda;<br>- Evolução para situação de coma com suspeita de acidente vascular encefálico hemorrágico.
**Desenvolvimento de sinais de gravidade local** (ver AO9)	- Sinais de necrose que necessite amputação e debridamento;<br>- Formação de abscesso com baixa resposta antibiótica para tratamento de infecção secundária.
**Reação Adversa ao soro** (ver AO4 e AO5)	- Reação adversa precoce: quando ocorrer qualquer sinal de reação imediatamente à administração do soro como prurido, rouquidão, rash cutâneo, relato de sufocamento, aumento da temperatura corporal;<br>- Parar a infusão imediatamente e administrar as drogas indicadas no AO4 (adrenalina, broncodilatador, anti-histamínico e corticosteroides).

Observação:

Os encaminhamentos **DEVEM** seguir o Sistema de Regulação (SISTER ou SISREG).

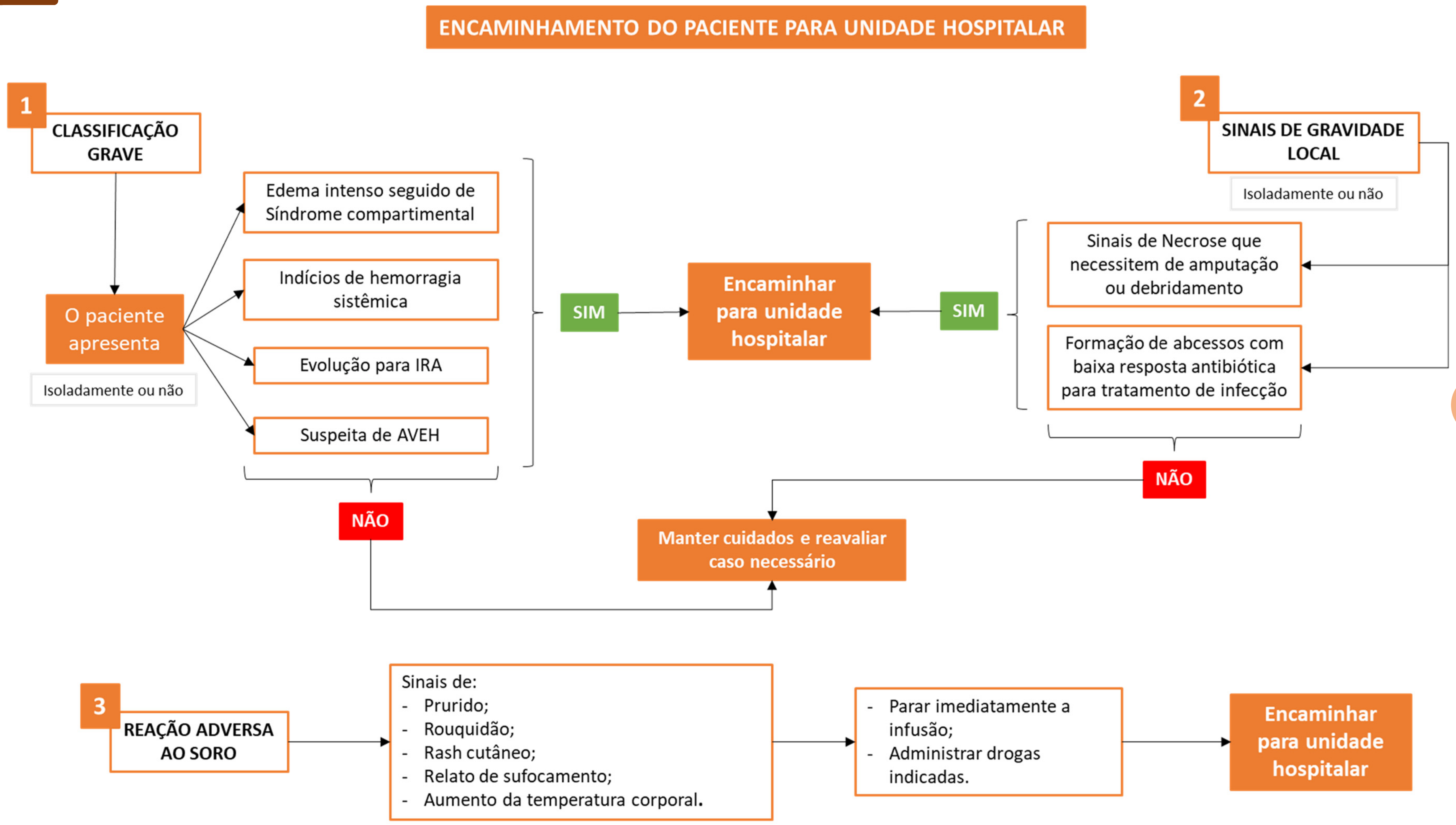
ENCAMINHAMENTO DO PACIENTE PARA UNIDADE HOSPITALAR
1
CLASSIFICAÇÃO GRAVE
O paciente apresenta
Isoladamente ou não
Edema intenso seguido de Síndrome compartimental
Indícios de hemorragia sistêmica
Evolução para IRA
Suspeita de AVEH
SIM
NÃO
Encaminhar para unidade hospitalar
2
SINAIS DE GRAVIDADE LOCAL
Isoladamente ou não
Sinais de Necrose que necessitem de amputação ou debridamento
Formação de abcessos com baixa resposta antibiótica para tratamento de infecção
SIM
NÃO
Manter cuidados e reavaliar caso necessário
3
REAÇÃO ADVERSA AO SORO
Sinais de:
- Prurido;
- Rouquidão;
- Rash cutâneo;
- Relato de sufocamento;
- Aumento da temperatura corporal.
- Parar imediatamente a infusão;
- Administrar drogas indicadas.
Encaminhar para unidade hospitalar

AO9 – Avaliação das complicações do acidente ofídico

O paciente deve ser avaliado diariamente para identificação de melhora do quadro ou discussão de novas condutas. As complicações apresentam sinais e sintomas geralmente após 48 horas do envenenamento.

Conduta:

1. O paciente deve ser avaliado diariamente após o momento crítico (primeiras 48 horas) com o foco no aparecimento de complicações decorrentes do acidente ofídico com envenenamento.

2. Avaliação das complicações do envenenamento mais prevalentes, especialmente infeção secundária, risco da perda funcional do membro acometido devido a necrose extensa e insuficiência renal aguda (IRA).

3. A avaliação clínica e laboratorial é primordial para detecção precoce das complicações e que podem reduzir a o dano ao paciente.

4. Avaliação Clínica:

   - Insuficiência Renal: considerar o critério de AKIN para diagnóstico e classificação da IRA:

Estágio	Creatinina Sérica	Diurese
Estágio 1	Aumento de 0,3 mg/dl ou aumento de 150-200% do valor basal (1,5 a 2 vezes)	< 0,5 ml/Kg/h por 6 horas
Estágio 2	Aumento > 200-300% do valor basal (> 2- 3 vezes)	< 0,5 ml/Kg/h por > 12 horas
Estágio 3	Aumento > 300% do valor basal (> 3 vezes ou Cr sérica ≥ 4,0 mg/dl com aumento agudo de pelo menos 0,5 mg/dl)	< 0,3 ml/Kg/h por 24 horas ou anúria por 12 horas

Somente um dos critérios (Cr ou diurese) pode ser utilizado para inclusão no estágio. Pacientes que necessitem de diálise são considerados estágio 3, independente do estágio em que se encontravam no início da terapia dialítica.

- **Infecção Secundária:** sinais locais de dor intensa e persistente, vermelhidão localizada, celulite e abscesso. Nos abscessos, devem ser realizados prescrição de antibiótico e drenagem da secreção. Para sinais sistêmicos, atentar para febre, náusea, vômito, rebaixamento de nível de consciência e sepse. Para antibioticoterapia, são indicados algumas das opções:

Droga	Dosagem
Clindamicina	IV ou VO
Amoxicilina + ácido clavulânico	VO
Ceftriaxona + metronidazol	IV

5. Avaliação Laboratorial:

Complicação	Exame laboratorial
Insuficiência Renal Aguda	Creatinina<br>Uréia
Infecção Secundária	Leucograma com diferencial

- Os casos com diagnóstico de infecção secundária, necrose extensa que necessite procedimento cirúrgico ou insuficiência renal aguda devem ser encaminhados para a unidade de referência.

AVALIAÇÃO DAS COMPLICAÇÕES DO ACIDENTE OFÍDICO

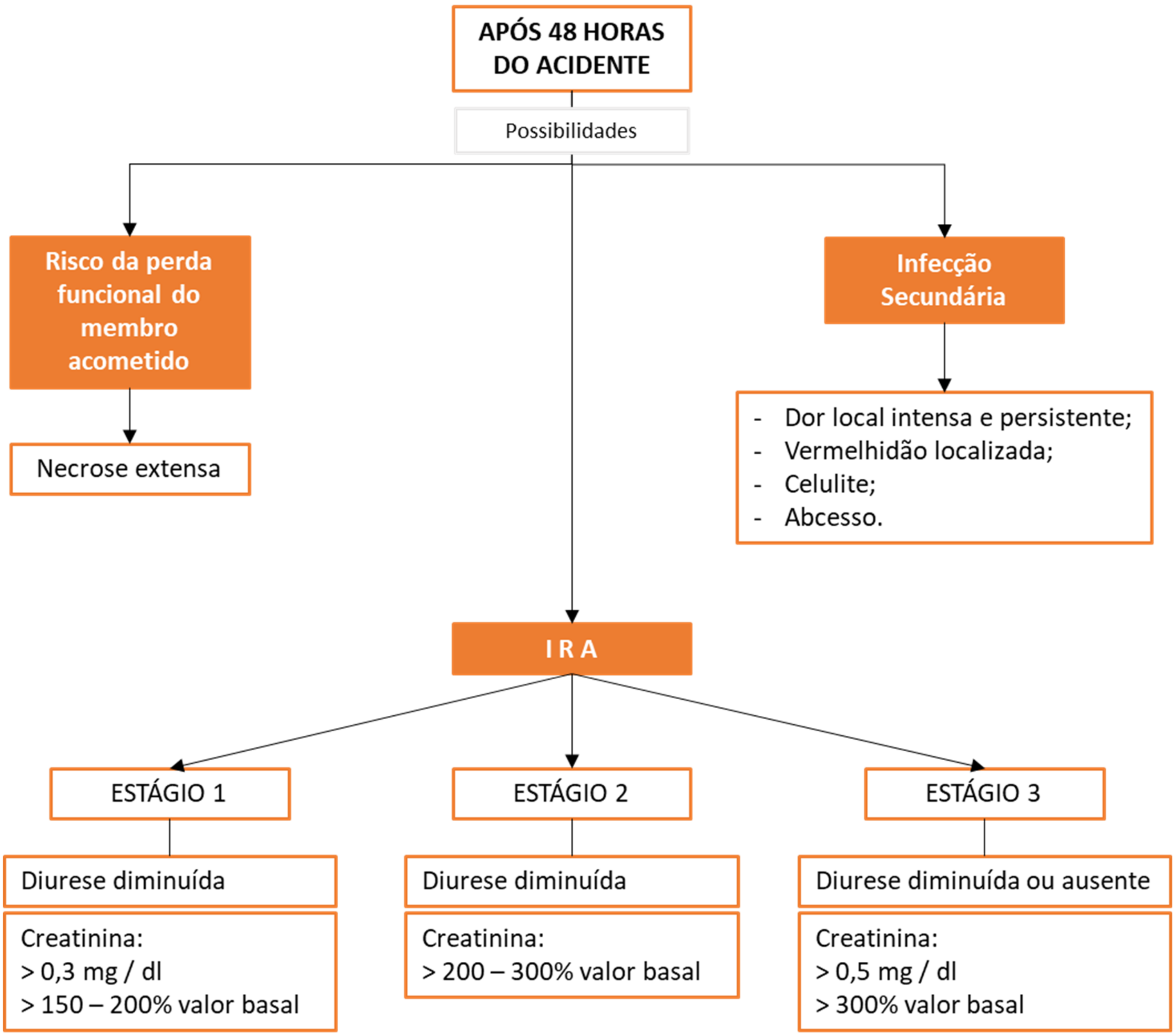

AO10 – Alta hospitalar e acompanhamento ambulatorial

O paciente deve ser avaliado diariamente para identificação de melhora do quadro e definição da alta hospitalar.

Conduta:

1. Acompanhamento ambulatorial pós-alta: pelo menos 1 avaliação após a alta hospitalar deverá ser agendada para um intervalo de 7 (sete) dias e deverá avaliar sinais e sintomas no local da picada como necrose, úlcera e infeção, reação tardia do soro antiofídico (nódulos, prurido), relato de diminuição de volume urinário, sangramentos em pele e mucosa.

2. Se necessário, realizar acompanhamento fisioterápico para melhor restabelecimento da mobilidade no membro afetado.

3. No caso de a lesão ofídica estar aberta no momento da alta o paciente deverá ser orientado a procurar a unidade básica de saúde mais próxima para curativos e avaliações diariamente.

AO11 – Alta da unidade básica e acompanhamento

O paciente deve ser avaliado diariamente para identificação de melhora do quadro e definição da alta da unidade básica.

Conduta:

1. Critérios para alta da unidade básica:
    a. Ausência de sinais inflamatórios (dor, calor, rubor e edema), que permitam o paciente deambular sozinho.
    b. Não apresentar suspeita de infecção secundária observando instalação de celulite ou abscesso (ver AO9).
    c. Não necessitar mais de medicação contínua como analgésico, antibiótico.

toxins

MDPI

AO12 – Preenchimento da ficha de notificação

A ficha de notificação de acidentes por animais peçonhentos deve ser preenchida em todos os casos de acidentes por serpentes, peçonhentas ou não, com ou sem utilização de antiveneno.

Conduta:

1. Parte 1 – Dados sociodemográficos: preencher com o maior número de informações possíveis para identificação das características dos pacientes

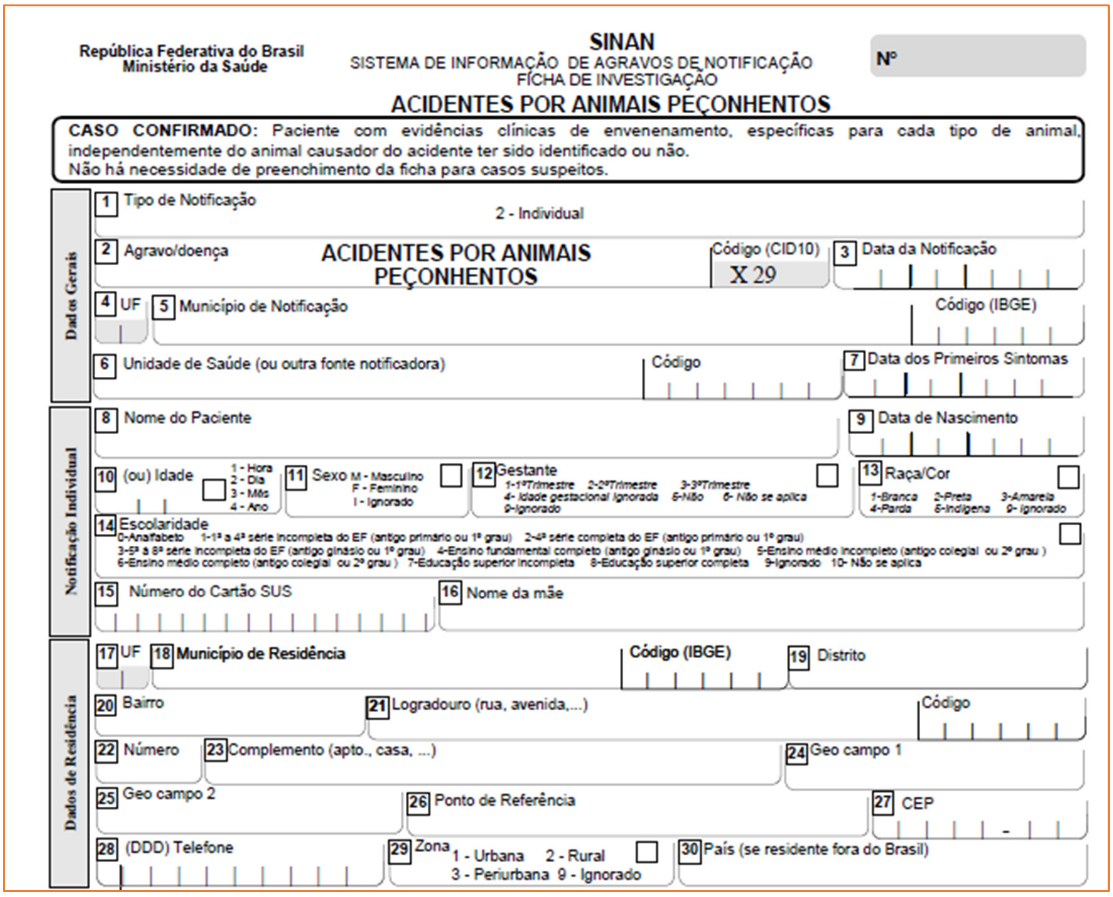

República Federativa do Brasil
Ministério da Saúde

SINAN
SISTEMA DE INFORMAÇÃO DE AGRAVOS DE NOTIFICAÇÃO
FICHA DE INVESTIGAÇÃO
ACIDENTES POR ANIMAIS PEÇONHENTOS

Nº

CASO CONFIRMADO: Paciente com evidências clínicas de envenenamento, específicas para cada tipo de animal, independentemente do animal causador do acidente ter sido identificado ou não.
Não há necessidade de preenchimento da ficha para casos suspeitos.

Dados Gerais

1 Tipo de Notificação — 2 - Individual

2 Agravo/doença — ACIDENTES POR ANIMAIS PEÇONHENTOS — Código (CID10) X 29

3 Data da Notificação

4 UF

5 Município de Notificação — Código (IBGE)

6 Unidade de Saúde (ou outra fonte notificadora) — Código

7 Data dos Primeiros Sintomas

Notificação Individual

8 Nome do Paciente

9 Data de Nascimento

10 (ou) Idade — 1 - Hora 2 - Dia 3 - Mês 4 - Ano

11 Sexo M - Masculino F - Feminino I - Ignorado

12 Gestante 1-1ºTrimestre 2-2ºTrimestre 3-3ºTrimestre 4- Idade gestacional Ignorada 5-Não 6- Não se aplica 9-Ignorado

13 Raça/Cor 1-Branca 2-Preta 3-Amarela 4-Parda 5-Indígena 9- Ignorado

14 Escolaridade
0-Analfabeto 1-1ª a 4ª série incompleta do EF (antigo primário ou 1º grau) 2-4ª série completa do EF (antigo primário ou 1º grau) 3-5ª à 8ª série incompleta do EF (antigo ginásio ou 1º grau) 4-Ensino fundamental completo (antigo ginásio ou 1º grau) 5-Ensino médio incompleto (antigo colegial ou 2º grau) 6-Ensino médio completo (antigo colegial ou 2º grau) 7-Educação superior incompleta 8-Educação superior completa 9-Ignorado 10- Não se aplica

15 Número do Cartão SUS

16 Nome da mãe

Dados de Residência

17 UF

18 Município de Residência — Código (IBGE)

19 Distrito

20 Bairro

21 Logradouro (rua, avenida,...) — Código

22 Número

23 Complemento (apto., casa, ...)

24 Geo campo 1

25 Geo campo 2

26 Ponto de Referência

27 CEP

28 (DDD) Telefone

29 Zona 1 - Urbana 2 - Rural 3 - Periurbana 9 - Ignorado

30 País (se residente fora do Brasil)

2. Parte 2 – Identificação do acidente: verificar no prontuário do paciente todas as informações registradas para o preenchimento desta parte. Nos casos que o profissional tiver contato com o paciente, as informações da ficha podem ser completadas por entrevista e complementação do registro no prontuário. O

campo número 46 deve ser registrado caso a serpente seja identificada ou os sinais e sintomas sejam compatíveis com o tipo da serpente.

Dados Complementares do Caso

Antecedentes Epidemiológicos

31 Data da Investigação
32 Ocupação
33 Data do Acidente
34 UF
35 Município de Ocorrência do Acidente:
Código (IBGE)
36 Localidade de Ocorrência do Acidente:
37 Zona de Ocorrência
1 - Urbana 2 - Rural 3 - Periurbana 9 - Ignorado
38 Tempo Decorrido Picada/Atendimento
1) 0⊢1h 2) 1⊢3h 3) 3⊢6h 4) 6⊢12h 5) 12⊢24 h 6) 24 e + h 9) Ignorado
39 Local da Picada
01 - Cabeça 02 - Braço 03 - Ante-Braço 04 - Mão 05 - Dedo da Mão 06 - Tronco
07 - Coxa 08 - Perna 09 - Pé 10 - Dedo do Pé 99 - Ignorado

Dados Clínicos

40 Manifestações Locais
1 - Sim 2 - Não 9 - Ignorado
41 Se Manifestações Locais Sim, especificar: 1 - Sim 2 - Não 9 - Ignorado
Dor Edema Equimose Necrose Outras (Espec.)
42 Manifestações Sistêmicas
1 - Sim
2 - Não
9 - Ignorado
43 Se Manifestações Sistêmicas Sim, especificar: 1 - Sim 2 - Não 9 - Ignorado
neuroparalíticas (ptose palpebral, turvação visual)
hemorrágicas (gengivorragia, outros sangramentos)
vagais (vômitos, diarréias)
miolíticas/hemolíticas (mialgia, anemia, urina escura)
renais (oligúria/anúria)
Outras (Espec.)
44 Tempo de Coagulação
1 - Normal
2 - Alterado
9 - Não realizado

Dados do Acidente

45 Tipo de Acidente
1 - Serpente 2 - Aranha 3 - Escorpião 4 - Lagarta
5 - Abelha 6 - Outros 9 - Ignorado
46 Serpente - Tipo de Acidente
1 - Botrópico 2 - Crotálico 3 - Elapídico 4 - Laquético
5 - Serpente Não Peçonhenta 9 - Ignorado
47 Aranha - Tipo de Acidente
1 - Foneutrismo 2 - Loxoscelismo 3 - Latrodectismo
4 - Outra Aranha 9 - Ignorado
48 Lagarta - Tipo de Acidente
1 - Lonomia 2 - Outra lagarta 9 - Ignorado

Animais Peçonhentos Sinan Net SVS 19/01/2006

3. Parte 3 – Completar com o maior número de informações possíveis e atentar para o registro do tipo de antiveneno e o número de ampolas utilizadas. A evolução do caso deve ser registrada, bem como as complicações ocorridas com o paciente.

Tratamento

49 Classificação do Caso
1 - Leve 2 - Moderado 3 - Grave 9 - Ignorado
50 Soroterapia
1 - Sim 2 - Não 9 - Ignorado
51 Se Soroterapia Sim, especificar número de ampolas de soro:
Antibotrópico (SAB) Anticrotálico (SAC) Antiaracnídico (SAAr)
Antibotrópico-laquético (SABL) Antielapídico (SAE) Antiloxoscélico (SALox)
Antilbotrópico-crotálico (SABC) Antiescorpiônico (SAEs) Antilonômico (SALon)
52 Complicações Locais
1 - Sim 2 - Não 9 - Ignorado
53 Se Complicações Locais Sim, especificar: 1 - Sim 2 - Não 9 - Ignorado
Infecção Secundária Necrose Extensa Síndrome Compartimental Déficit Funcional Amputação
54 Complicações Sistêmicas
1 - Sim 2 - Não 9 - Ignorado
55 Se Complicações Sistêmicas Sim, especificar: 1 - Sim 2 - Não 9 - Ignorado
Insuficiência Renal Insuficiência Respiratória / Edema Pulmonar Agudo Septicemia Choque

Conclusão

56 Acidente Relacionado ao Trabalho
1 - Sim
2 - Não
9 - Ignorado
57 Evolução do Caso
1-Cura 2-Óbito por acidentes por animais peçonhentos
3-Óbito por outras causas 9-Ignorado
58 Data do Óbito
59 Data do Encerramento

4. Parte 4 – utilizar esta parte da ficha para consulta rápida dos efeitos do veneno referentes aos sinais locais e sistêmicos.

toxins

MDPI

Acidentes com animais peçonhentos: manifestações clínicas, classificação e soroterapia

	Tipo	Manifestações Clínicas	Tipo Soro	Nº ampolas
OFIDISMO	**Botrópico** *jararaca jararacuçu urutu caiçaca*	Leve: dor, edema local e equimose discreto	SAB	2 - 4
		Moderado: dor, edema e equimose evidentes, manifestações hemorrágicas discretas		4 - 8
		Grave: dor e edema intenso e extenso, bolhas, hemorragia intensa, oligoanúria, hipotensão		12
	**Crotálico** *cascavel boicininga*	Leve: ptose palpebral, turvação visual discretos de aparecimento tardio, sem alteração da cor da urina, mialgia discreta ou ausente	SAC	5
		Moderado: ptose palpebral, turvação visual discretos de inicio precoce, mialgia discreta, urina escura		10
		Grave: ptose palpebral, turvação visual evidentes e intensos, mialgia intensa e generalizada, urina escura, oligúria ou anúria		20
	**Laquético** *surucuru pico-de-jaca*	Moderado: dor, edema, bolhas e hemorragia discreta	SABL	10
		Grave: dor, edema, bolhas, hemorragia, cólicas abdominais, diarréia, bradicardia, hipotensão arterial		20
	**Elapídico** *coral verdadeira*	Grave: dor ou parestesia discreta, ptose palpebral, turvação visual	SAEL	10
ESCORPIONISMO	**Escorpiônico** *escorpião*	Leve: dor, eritema e parestesia local	SAEsc ou SAA	—
		Moderado: sudorese, náuseas, vômitos ocasionais, taquicardia, agitação e hipertensão arterial leve		2 - 3
		Grave: vômitos profusos e incoercíveis, sudorese profusa, prostração, bradicardia, edema pulmonar agudo e choque		4 - 6
ARANEÍSMO	**Loxoscélico** *aranha-marrom*	Leve: lesão incaracterística sem aranha identificada	SAA ou SALox	—
		Moderado: lesão sugestiva com equimose, palidez, eritema e edema endurado local, cefaléia, febre, exantema		5
		Grave: lesão característica, hemólise intravascular		10
	**Foneutrismo** *aranha-armadeira aranha-da-banana*	Leve: dor local	SAA	—
		Moderado: sudorese ocasional, vômitos ocasionais, agitação, hipertensão arterial		2 - 4
		Grave: sudorese profusa, vômitos freqüentes, priapismo, edema pulmonar agudo, hipotensão arterial		5 - 10
LONOMIA	*taturana oruga*	Leve: dor, eritema, adenomegalia regional, coagulação normal, sem hemorragia	SALon	—
		Moderado: alteração na coagulação, hemorragia em pele e/ou mucosas		5
		Grave: alteração na coagulação, hemorragia em vísceras, insuficiência renal		10

Informações complementares e observações

Anotar todas as informações consideradas importantes e que não estão na ficha (ex: outros dados clínicos, dados laboratoriais, laudos de outros exames e necrópsia, etc.)

Investigador

Município/Unidade de Saúde | Cód. da Unid. de Saúde

Nome | Função | Assinatura

Animais Peçonhentos | Sinan Net | SVS 19/01/2006

Observações:

Mesmo os acidentes que não necessitaram de antiveneno, devem ter o preenchimento da ficha de notificação para auxiliar no banco de dados nacional e caracterização dos tipos de serpentes que ocasionam acidentes no território brasileiro.

AO13 – Recebimento e armazenamento de antiveneno

O recebimento e armazenamento de antiveneno devem ser minuciosamente realizados e registrados para garantir o tratamento adequado nos locais que possuam rede de frios, ou seja, nos estabelecimentos de saúde que dispensam imunobiológicos como os antivenenos.

Conduta:

1. Receber e verificar a integridade das embalagens e ampolas de antivenenos, bem como registrar o número recebido e a condição física (integridade do frasco e líquido interno sem precipitados/grumos) do antiveneno;
2. Realizar a verificação da temperatura utilizada no transporte e a temperatura do local onde será armazenada (2 a 8◦C);
3. Planejar o pedido periódico dos antivenenos conforme a demanda de utilização local;
4. Remanejar os antivenenos para outros serviços quando a quantidade armazenada ultrapassa a necessidade local ou quando a data de validade está próxima do vencimento sem perspectiva de utilização prévia;
5. Controlar o acesso às áreas de armazenamento para pessoas não autorizadas para não interferir na segurança do armazenamento;
6. Realizar uma rotina diária de registro e controle de temperatura e/ou umidade nas salas de distribuição, de recepção e de inspeção e sala de armazenagem e controle, bem como internamente nos equipamentos refrigeradores de armazenamento;
7. Ter um plano de contingência para todos os equipamentos de refrigeração nos casos de falta de energia elétrica ou situações que possam apresentar risco potencial;
8. Realizar uma rotina de capacitação e treinamento dos recursos humanos para controle e manipulação dos antivenenos;
9. Realizar corretamente o descarte de resíduos dos antivenenos conforme a orientação da sua central de distribuição.

RECEBIMENTO E ARMAZENAMENTO DE ANTIVENENO

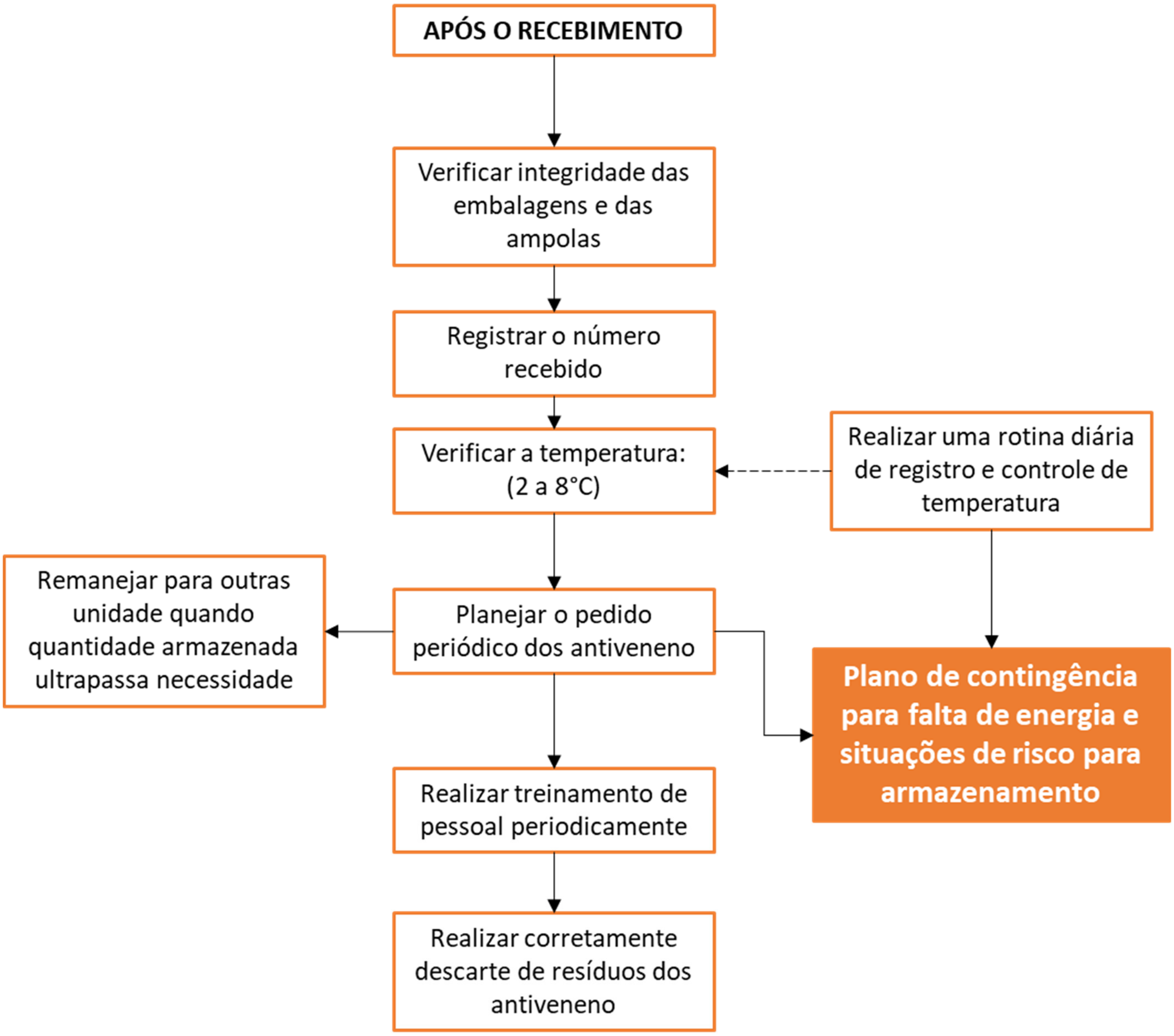

Referências consultadas

AFONSO C; AZEVEDO GAM; ALVES P. Prevenção e tratamento de feridas: da evidência à prática. 1ª ed. Prior Velho: HARTMANN Portugal, 2014.

AHA. Adult Basic Life Support. 2020 International Consensus on Cardiopulmonary Resuscitation and Emergency Cardiovascular Care Science With Treatment Recommendations. Circulation. 2020;142:S41-S91.

ALBUQUERQUE PLMM, PAIVA JHHGL, MARTINS AMC, MENESES GC, SILVA GB, BUCKLEY N, ET AL. Clinical assessment and pathophysiology of *Bothrops* venom-related acute kidney injury: a scoping review. J Venom Anim Toxins Incl Trop Dis. 2020;26:e20190076.

BRASIL. MINISTÉRIO DA SAÚDE. Secretaria de Vigilância em Saúde. Coordenação-Geral de Desenvolvimento da Epidemiologia em Serviços. Capítulo 11 - Animais Peçonhentos. Ministério da Saúde: Brasília. Brasília. 5º ed. 2021.

BRASIL. MINISTÉRIO DA SAÚDE. Secretaria de Vigilância em Saúde. Departamento de Vigilância das Doenças Transmissíveis. Manual de Normas e Procedimentos para Vacinação. Ministério da Saúde: Brasília. Brasília, 2014.

BRASIL. MINISTÉRIO DA SAÚDE. Fundação Nacional de Saúde. Manual de Rede de Frio. 3. ed. Ministério da Saúde: Brasília. Brasília, 2001.

CARDOSO JLC; FRANÇA FOS; FAN HW; MALAQUE CMS; HADDAD JRV. Animais Peçonhentos no Brasil. Biologia, Clínica e Terapêutica dos Acidentes. 1. ed. São Paulo: Sarvier/Fapesp, 2003. v. 1. 468p .

CRISTINO JS; SALAZAR GM; MACHADO VA; HONORATO E; FARIAS AS; VISSOCI JRN; et al. A painful journey to antivenom: The therapeutic itinerary of snakebite patients in the Brazilian Amazon (The QUALISnake Study). PLoS Negl Trop Dis. 2021;15:e0009245.

FAN HW, MARCOPITO LF, CARDOSO JL, FRANÇA FO, MALAQUE CM, FERRARI RA, THEAKSTON RD, WARRELL DA. Sequential randomised and double blind trial of promethazine prophylaxis against early anaphylactic reactions to antivenom for *Bothrops* snake bites. BMJ. 1999;318:1451-2.

GIMENES SNC; SACHETT JAG; COLOMBINI M; FREITAS-DE-SOUSA LA; IBIAPINA HNS; COSTA AG; et al. Observation of *Bothrops atrox* Snake Envenoming Blister Formation from Five Patients: Pathophysiological Insights. Toxins. 2021;13:800.

INSTITUTO BUTANTAN. Soro antibotrópico (pentavalente) e anticrotálico. 2017. Disponível: http://butantan.gov.br/assets/pdf/soros_vacinas/soros/Bula-Soro-Antibotropico-Crotalico-Instituto-Butantan-Paciente-Consulta-Remedios.pdf.

JORGE MT; CARDOSO JL; CASTRO SC; RIBEIRO L; FRANÇA FO; ALMEIDA ME, et al. A randomized 'blinded' comparison of two doses of antivenom in the treatment of *Bothrops* envenoming in São Paulo, Brazil. Trans R Soc Trop Med Hyg. 1995;89:111-4.

JORGE MT; MALAQUE C; RIBEIRO LA; FAN HW; CARDOSO JL; NISHIOKA SA; et al. Failure of chloramphenicol prophylaxis to reduce the frequency of abscess formation as a complication of envenoming by *Bothrops* snakes in Brazil: a double-blind randomized controlled trial. Trans R Soc Trop Med Hyg. 2004;98:529-34.

JORGE MT, RIBEIRO LA, O'CONNELL JL. Prognostic factors for amputation in the case of envenoming by snakes of the *Bothrops* genus (Viperidae). Ann Trop Med Parasitol. 1999;93:401-8.

KELLUM JA; LAMEIRE N; ASPELIN P; BARSOUM RS; BURDMANN EA; GOLDSTEIN SL; et al. KDIGO Clinical Practice Guideline for Acute Kidney Injury. Kidney Int Suppl. 2012;2:124-38.

MONTEIRO WM; CONTRERAS-BERNAL JC; BISNETO PF; SACHETT J; MENDONÇA DA SILVA I; LACERDA M; et al. *Bothrops atrox*, the most important snake involved in human envenomings in the amazon: How venomics contributes to the knowledge of snake biology and clinical toxinology. Toxicon: X. 2020;1:100037.

OLIVEIRA SS; ALVES EC; SANTOS AS; NASCIMENTO EF; PEREIRA JPT; DA SILVA IM; et al. Snakebites in the Amazon: recovery from hemostatic disorders after Brazilian antivenom therapy. Clin Toxicol. 2019; 58:266-74.

OLIVEIRA S; ALVES E; SANTOS A; PEREIRA J; SARRAFF L; NASCIMENTO E; et al. Factors Associated with Systemic Bleeding in *Bothrops* Envenomation in a Tertiary Hospital in the Brazilian Amazon. Toxins. 2019;11:22.

PARDAL PP; SOUZA SM; MONTEIRO MR; FAN HW; CARDOSO JL; FRANÇA FO; et al. Clinical trial of two antivenoms for the treatment of *Bothrops* and *Lachesis* bites in the north eastern Amazon region of Brazil. Trans R Soc Trop Med Hyg. 2004;98:28-42.

POTTER PA; PERRY AG; ELKIN MK. Procedimentos e intervenções de enfermagem. 5ª ed. Rio de Janeiro: Elsevier, 2013.

PUCCA MB; KNUDSEN C; OLIVEIRA IS; RIMBAULT C; CERNI F; WEN FH; et al. Current Knowledge on Snake Dry Bites. Toxins. 2020;12:668.

RIBEIRO LA; JORGE MT; LEBRÃO ML. Prognostic factors for local necrosis in *Bothrops jararaca* (Brazilian pit viper) bites. Trans R Soc Trop Med Hyg. 2001;95:630-4.

SACHETT JAG; SILVA IM; ALVES EC; OLIVEIRA SS; SAMPAIO VS; VALE FF; et al. Poor efficacy of preemptive amoxicillin clavulanate for preventing secondary infection from *Bothrops* snakebites in the Brazilian Amazon: A randomized controlled clinical trial. PLoS Neg Trop Dis. 2017;11:e0005745.

SOUSA JDB; MARQUES HO; FAN HW; OLIVEIRA SS; LACERDA MVG; SACHETT JAG; et al. Accuracy of the Lee-White Clotting Time Performed in the Hospital Routine to Detect Coagulopathy in *Bothrops atrox* Envenomation. Am J Trop Med Hyg. 2018; 81:1547.

SOUZA AS; SACHETT JAG; ALCÂNTARA JA; FREIRE M; ALECRIM MGC; LACERDA M; et al. Snakebites as cause of deaths in the Western Brazilian Amazon: Why and who dies? Toxicon. 2018; 145:15-24.

STEVENS DL; BISNO AL; CHAMBERS HF; DELLINGER EP; GOLDSTEIN EJC; GORBACH SL; et al. Practice guidelines for the diagnosis and management of skin and soft tissue infections: 2014 update by the infectious diseases society of America. Clin Infect Dis. 2014;59:1-43.

toxins

MDPI

SAVING

Snake Antivenom Immunoglobulins

NEED TO BE GUARANTEED

UEA UNIVERSIDADE DO ESTADO DO AMAZONAS

MINISTÉRIO DA SAÚDE

Instituto de Pesquisa Clínica Carlos Borborema

CEPCLAM Centro de Pesquisa Clínica em Envenenamento por Animais

INSTITUTO BUTANTAN A serviço da vida

FVS FUNDAÇÃO DE VIGILÂNCIA EM SAÚDE DO AMAZONAS
