# Supplementary material for: Validation of a Culturally Relevant Snakebite Envenomation Clinical Practice Guideline in Brazil
Source: Toxins (Basel). 2022 May 28;14(6):376. doi: 10.3390/toxins14060376 (PMC9229773; doi:10.3390/toxins14060376)
Supplement: Supplementary file 1 [file toxins-14-00376-s001.zip › Table S1.pdf]

toxins

MDPI

Supplementary material

Table S1. Semi-structured interview guide used in the focal group for care practice guideline validation by target users.

Questions	Areas expected to be covered during the discussion
1. Who here has treated someone that has been bitten by a snake? Can you tell us about your experience?	a) Place of treatment (at the hospital or elsewhere)<br>b) Severity of the cases<br>c) Complications/outcomes<br>d) Use of antivenom<br>e) Traditional remedies and self-care procedures observed<br>f) Where the knowledge that was used in the treatment of these cases came from
2. What subjects most interest you in this training course?	a) Specific topics/classes<br>b) Added knowledge<br>c) Conduct in practice<br>d) Subjects that are more difficult to understand
3. How can this care practice guideline (CPG) contribute to the medical assistance you will provide in the future?	a) Feel more prepared/confident<br>b) Possibility of applying the CPG in the municipality where I work
4. What suggestions would you give for improving the CPG?	a) Contents to be added, deleted or improved<br>b) Need for structure and presentation improvements
